# Supplementary material for: Identification and disruption of a neural mechanism for accumulating prospective metacognitive information prior to decision-making
Source: Neuron. 2021 Apr 21;109(8):1396–1408.e7. doi: 10.1016/j.neuron.2021.02.024 (PMC8063717; doi:10.1016/j.neuron.2021.02.024)
Supplement: Document S1. Figures S1–S8 [file mmc1.pdf]

Neuron, Volume *109*

Supplemental information

Identification and disruption of a neural mechanism for accumulating prospective metacognitive information prior to decision-making

Kentaro Miyamoto, Nadescha Trudel, Kevin Kamermans, Michele C. Lim, Alberto Lazari, Lennart Verhagen, Marco K. Wittmann, and Matthew F.S. Rushworth

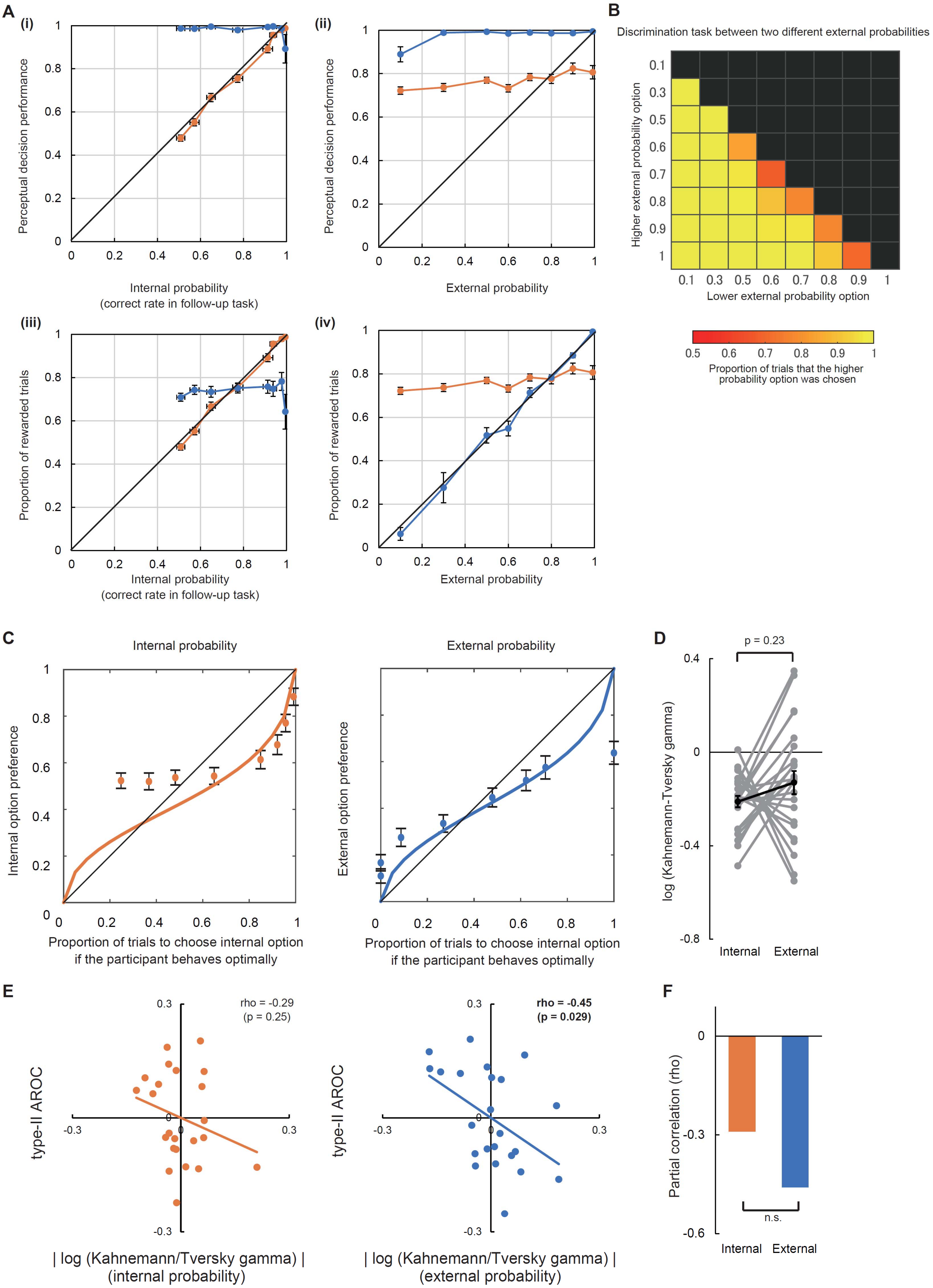
A
(i)
Perceptual decision performance
Internal probability
(correct rate in follow-up task)
(ii)
Perceptual decision performance
External probability
(iii)
Proportion of rewarded trials
Internal probability
(correct rate in follow-up task)
(iv)
Proportion of rewarded trials
External probability
B
Discrimination task between two different external probabilities
Higher external probability option
Lower external probability option
Proportion of trials that the higher probability option was chosen
C
Internal probability
Internal option preference
Proportion of trials to choose internal option if the participant behaves optimally
External probability
External option preference
Proportion of trials to choose internal option if the participant behaves optimally
D
p = 0.23
log (Kahnemann-Tversky gamma)
Internal
External
E
type-II AROC
rho = -0.29
(p = 0.25)
| log (Kahnemann/Tversky gamma) |
(internal probability)
type-II AROC
rho = -0.45
(p = 0.029)
| log (Kahnemann/Tversky gamma) |
(external probability)
F
Partial correlation (rho)
n.s.
Internal
External

Supplemental Figure 1. Comparative subjective utility functions for internal and external probabilities (related to Figure 2)

(A) (i) Proportion of correct trials (perceptual decision performance in the main task) in the second decision stage as a function of internal probability (based on follow-up task performance at each coherence level) offered in the first metacognitive judgment stage. The trials on which the internal probability option was chosen (orange) and on which the alternative, the external probability option was chosen (blue) are summarized independently. Note that the probability of correctly discerning the motion direction tightly scaled with the internal probability estimated from the follow-up task. By contrast, discerning the correct motion direction was trivially easy when opting for the external probability task and hence performance was at ceiling independent of what had been offered as the internal probability task. **(ii)** Proportion of correct trials in the second decision stage as a function of external probability. The trials on which the external probability option was chosen (blue) and unchosen (orange, i.e., internal probability option was chosen) are summarized independently. Note, again, that the quantity plotted on the y-axis related to the correct estimation of the dot direction, and that, after choosing the internal task, reward would always deterministically ensue after a correct estimation, whereas after choosing the external task and estimating the dot direction correctly, reward would only ensue with a given probability, i.e. with the external probability. **(iii– iv)** Same convention with as (i–ii) but summarized for proportion of rewarded trials instead of correct trials. Note that here, when comparing (iii) and (iv), it becomes apparent that the actual payoff from choosing each task tightly scales with internal and external probability, respectively, while the value of the alternative option is held constant. Together, this figure illustrates how the overall payoff was thus finely balanced between tasks, but that they were critically different with respect to how difficult it was to perform the tasks correctly in order to arrive at this payoff. On the top (i–ii) and bottom (iii–iv) panels, the lines for the trials on which the internal probability option was chosen (orange) are exactly the same because external probability was always set at 100% for internal probability options. **(B)** Performance on an additional control task in which participants compared two different levels of external probability. The colour within each cell indicates the probability with which participants chose the optimal external task. The optimal external task is simply the external task with a higher probability of reward, i.e., higher number of dots. The participants (n=4) could reliably choose the option with higher external probability (i.e. larger number of dots) (92.5±1.5

[mean±s.e.m.]%). (**C**) Subjective utility functions for internal (left) [or, external (right)] probability. Subjective probability (y-axis) is the proportion of trials that on which the internal [or, external] option was chosen in the metacognitive judgment stage. Objective probability (x-axis) is the proportion of the trials that the internal [or, external] probability option should have been chosen if participants behaved optimally (i.e., if they always picked the better probability option in the metacognitive judgment stage). Each dot is a given level of internal/external probability and increases from left to right (Note that the two dots on the y axis in the right panel indicate the data for 10% [lower] and 30% [higher] external probability, respectively). The data are fitted by typical subjective utility functions based on Prospect Theory (Kahneman and Tversky, 1979), where skewness is defined by a single parameter: gamma. (**D**) Comparative gamma between internal and external probabilities ($p = 0.23$) suggests that there is nothing fundamentally different about the way in which objective probabilities are translated into subjective estimates. It is noted that smaller gamma indicates larger distortion and, if the more the participant has a perfect undistorted utility function, the closer gamma comes close to 1 (log gamma comes close to 0). **(E)** Multiple regression analysis reveals that if a participant has a gamma closer to 1 for either internal (left) or external (right) probability then this predicts higher type-II A_{ROC} in the same participant. **(F)** Comparable sizes of gamma for both internal and external probabilities suggests that both probabilities contribute similarly to type-II A_{ROC}.

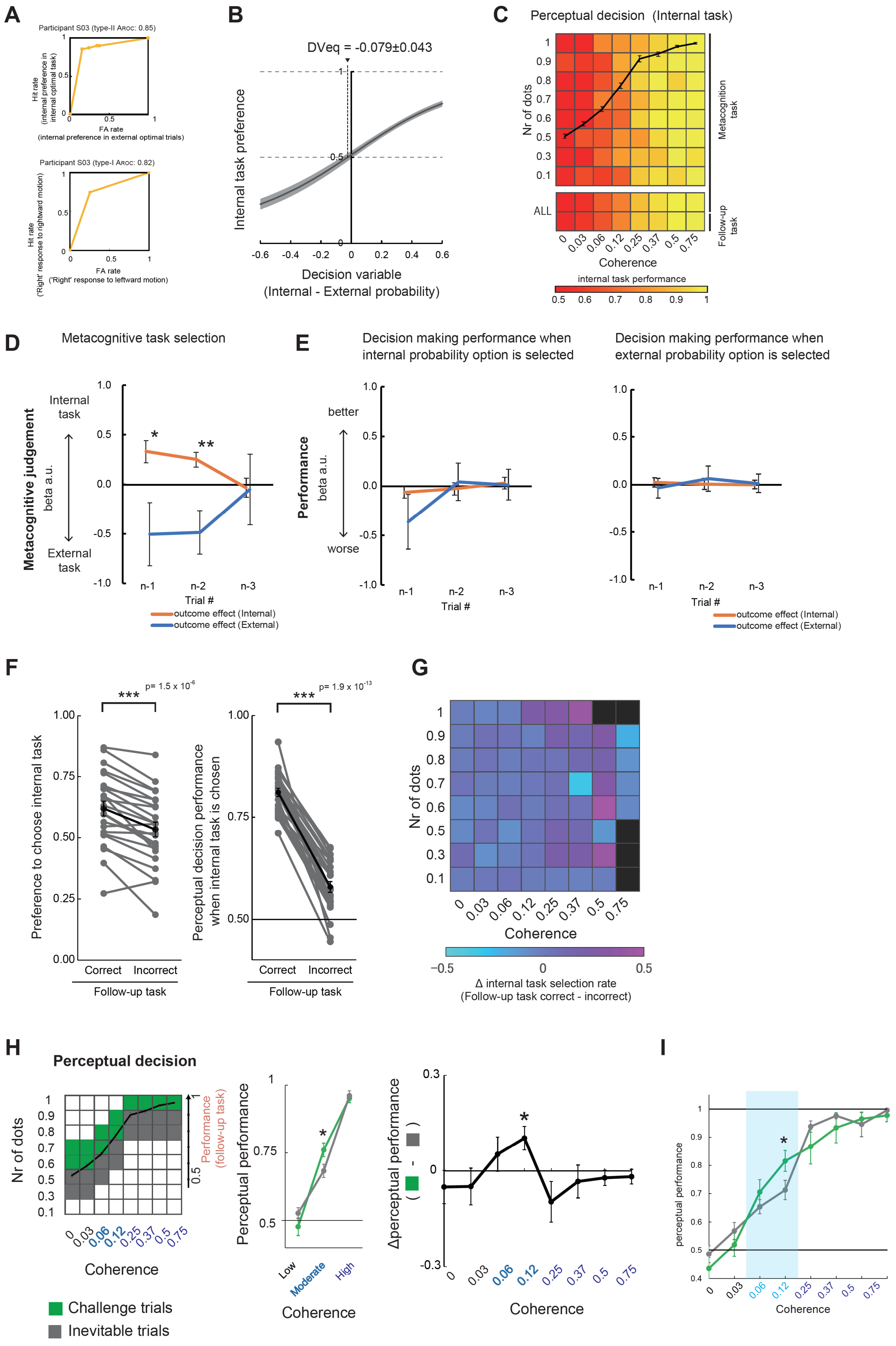
A
Participant S03 (type-II Aroc: 0.85)
Hit rate (internal preference in internal optimal task)
FA rate (internal preference in external optimal trials)
Participant S03 (type-I Aroc: 0.82)
Hit rate ('Right' response to rightward motion)
FA rate ('Right' response to leftward motion)
B
DVeq = -0.079±0.043
Internal task preference
Decision variable
(Internal - External probability)
C
Perceptual decision (Internal task)
Nr of dots
Metacognition task
ALL
Follow-up task
Coherence
internal task performance
D
Metacognitive task selection
Metacognitive judgement
beta a.u.
Internal task
External task
Trial #
outcome effect (Internal)
outcome effect (External)
E
Decision making performance when internal probability option is selected
Decision making performance when external probability option is selected
Performance
beta a.u.
better
worse
Trial #
outcome effect (Internal)
outcome effect (External)
F
p= 1.5 x 10^-6
p= 1.9 x 10^-13
Preference to choose internal task
Perceptual decision performance when internal task is chosen
Correct
Incorrect
Follow-up task
G
Nr of dots
Coherence
Δ internal task selection rate
(Follow-up task correct - incorrect)
H
Perceptual decision
Nr of dots
Performance (follow-up task)
Coherence
Challenge trials
Inevitable trials
Perceptual performance
Low
Moderate
High
Δperceptual performance
I
perceptual performance
Coherence

Supplemental Figure 2. Behavioral performance during prospective metacognition task (related to Figure 3).

(A) The type-II and type-I ROC curves of a representative participant (indicated with a black circle in the upper plot). The dots on the line represent the proportion of Hit over FA trials for specific coherence levels (see STAR Methods). **(B)** Preference for choosing the internal probability task as a function of decision variable (DV – the difference between the internal and external probabilities). **(C)** Performance of the internal probability task during the perceptual decision stage in the main metacognition task (where each perceptual decision was preceded by a metacognitive judgment). Performance in the follow-up task (which lacked any metacognitive judgment phase) is indicated by the black line in the square plot and color-coded in the rectangular plot below. Average performance across all probability levels is shown for the metacognitive judgment stage (upper bar) and follow up-task (lower bar) at the bottom of the panel. **(D–E)** There was a significant influence of outcome (reward/no-reward) received during the past three trials on the choice made in the metacognitive judgment stage (D). There was no influence of outcome (reward/no-reward) received during the past three trials on the performance of the internal task (E, left) and external task (E, right). Metacognitive judgments were also influenced by the outcomes of decisions made on previous trials. Whether or not reward was received for taking an internal probability option and for taking an external probability option had impacts on subsequent metacognitive judgments. The impacts were in opposite directions (two-way repeated ANOVA with the main effects of evidence type and time constant; main effect of internal/external evidence, $F_{1,22}$=7.65, p=0.011; main effect of time constant, $F_{2,44}$=0.06, p=0.94; interaction, $F_{2,44}$=2.46, p=0.096). The receipt of reward outcomes when internal probability options had been chosen recently increased the likelihood of selecting the internal probability in a subsequent metacognitive decision (one-way repeated ANOVA with the main effects of time constant, $F_{2,44}$=6.27, p=0.0040; paired t-test: $trial_{n-1}$ > $trial_{n-3}$, p=0.001, $trial_{n-2}$ > $trial_{n-3}$, p=0.011 with Bonferroni correction. $trial_{n-1}$, t_{22}=2.95, p=0.022; $trial_{n-2}$, t_{22}=3.36, p=0.0085, t-test against zero with Bonferroni correction; note that participants chose internal probability options on approximately 50% of trials). However, external probability options chosen more recently did not change the likelihood of selecting the external probability option significantly (one-way repeated ANOVA with the main effects of time constant, $F_{2,44}$=0.81, p=0.45; $trial_{n-1}$, t_{22}=-1.58, p=0.38; $trial_{n-2}$, t_{22}=-2.21, p=0.11, t-test against zero with Bonferroni correction). This difference also suggested that there are

independent mechanisms for evaluating and learning about internal and external probabilities. **(F)** Preference, in the metacognitive judgment stage of the main task, for choosing to perform the internal task trials as a function of whether the same trial would be performed correctly in the follow-up task. Left panel: First, we categorized metacognitive trials into correct and incorrect trials according to performance on the follow-up task. Second, we calculated the proportion of occasions the participant chose the internal vs external probability task option in the correct trial group and the incorrect trial group. Here, we plot the proportion of internal task choices when follow-up task performance was correct (left side of the x-axis) or incorrect trials (right side on the x-axis). Participants more often chose the internal probability option in the metacognitive judgment phase in the main experiment when they would subsequently classify motion direction correctly in the follow-up task (the average across participants, indicated as dots, is above 50% which represents a preference for the internal task) (t_{22}=6.49, p=1.5×10^{-6}) Right panel: The performance of the chosen internal probability task was also better in the perceptual decision stage in the main experiment for options that participants would subsequently classify correctly when they encountered them in the follow-up task (t_{22}=15.7, p=1.9×10^{-13}). **(G)** The difference in preference for choosing the internal probability option for options that were classified correctly in the follow-up task as opposed to those that were not classified correctly in the follow-up task. **(H)** It was confirmed that perceptual decision performance on the internal task at moderate coherence levels (0.06, 0.12) was higher when the internal task had been paired with an external probability option that was, on average, slightly more likely to yield reward (Challenge trials) than it was when the internal task was paired with an external probability option slightly less likely to yield reward on average (Inevitable trials) by defining Challenge and Inevitable trials as the trials two cells above and below the curve (two-way ANOVA, trial-type [Challenge, Inevitable] × coherence level [Low, Moderate, High]. Interaction: p=0.0166. Simple main effect for Challenge vs. Inevitable trials at moderate coherence: p=0.027). Again, this suggests that participants are able to estimate their likelihood of success if they perform the internal probability task. The black line in the square plot indicates performance in the follow-up task. **(I)** Perceptual decision performance on the internal task for Challenge trials (green) and Inevitable trials (grey) for each coherence level. For the two levels of moderate coherence (0.06 and 0.12) (blue shade), a two-way ANOVA (coherence [0.06 or 0.12] × trial type [Challenge or Inevitable]) revealed a significant main effect of Challenge/Inevitable trial type (p = 0.023) and a main effect of coherence (p = 0.037)

without any interaction ($p = 0.45$). (N=23 participants; *$p<0.05$, **$p<0.01$, ***$p<0.001$, t-test against chance-level, Bonferroni correction if required; error bars are SEM across participants).

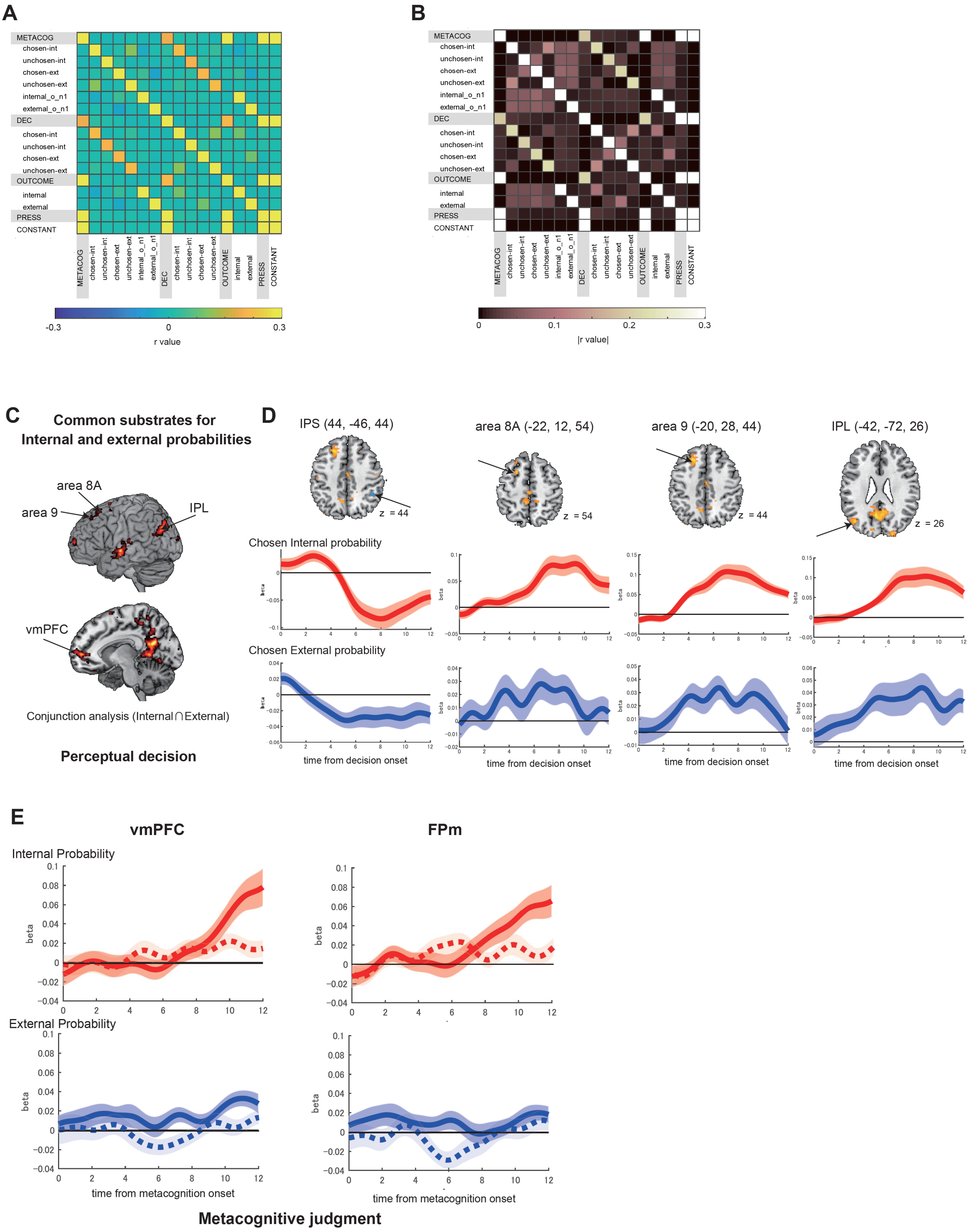

Supplemental Figure 3. Perceptual decision-making: common substrates for internal and external evidence accumulation in well-known areas for motion vision (related to Figure 4)

(A–B) Correlation between regressors included in the whole-brain fMRI and ROI analyses. Predictors included in fMRI-GLM1 and ROI-GLM1 (we included the same regressors in both analyses) between the metacognitive judgment stage and perceptual decision stage had a reasonable low correlation (maximum r value of 0.2). Although we used the same variables in both stages, the correlation between regressors was decreased by including a temporal jitter (see STAR Methods for details). The mean of the correlation coefficients (A) and the mean of the absolute correlation coefficients (B) are shown. **(C)** Activity in several frontal and parietal areas reflected the probability of reward associated with the chosen action – the evidence for making the chosen action – regardless of whether that was derived from consideration of internal or external probabilities at a liberal statistical criterion for display purpose (whole-brain effects family-wise error cluster corrected with $z>2.3$ and $p<0.05$). **(D)** The evolution of the regression weights across time indexing the impact of chosen internal probability (red at top) and chosen external probability (blue at bottom) on neural activity are illustrated for four example areas (IPS, area 8A, area 9, IPL). In each case the effects are seen after a short delay reflecting the hemodynamic response function. **(E)** The evolution of the regression weights across time in the metacognitive judgment stage indexing the impact of internal probability (red at upper panel of each area. solid line: chosen internal probability. dotted line: unchosen internal probability) and chosen external probability (blue at lower panel of each area. solid line: chosen external probability. dotted line: unchosen external probability) on neural activity are illustrated for two example areas: vmPFC and FPm.

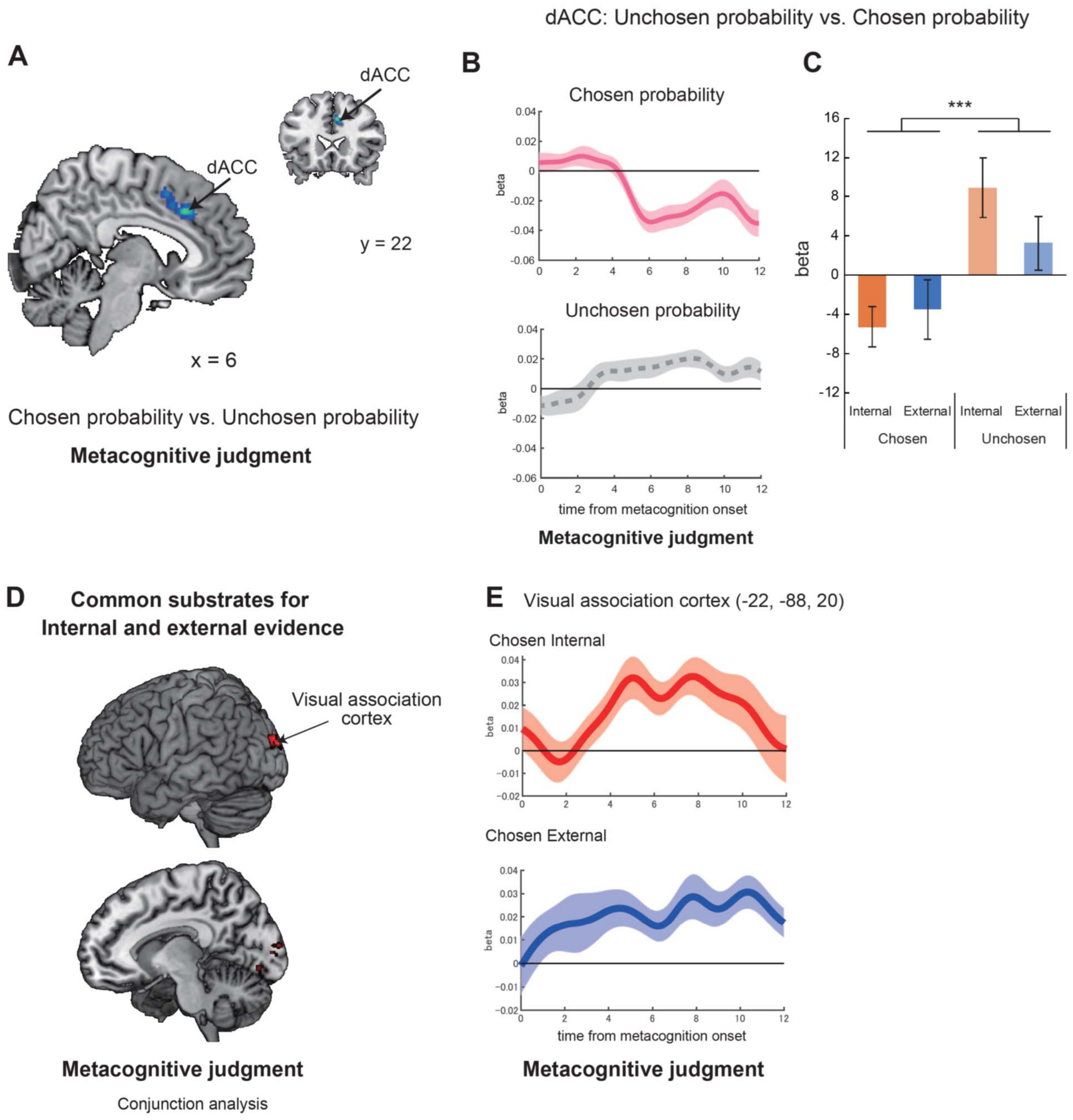

Supplemental Figure 4. Metacognitive judgment: common substrates for internal and external evidence accumulation in the anterior cingulate cortex and visual association cortex (related to Figure 5).

(A) Activity in dACC reflected both internal and external probabilities during the initial metacognitive decision on each trial. **(B)** Activity in dACC was positively modulated by probabilities associated with the option that was rejected during the metacognitive judgment, while its activity was negatively modulated by probabilities associated with the option that was taken. Similar patterns of activity change in dACC have been linked previously to the

evidence for taking an alternative or counterfactual choice as opposed to the choice actually made (Fouragnan et al., 2019; Kolling et al., 2018; Kolling et al., 2016). **(C)** This pattern of activity increase and decrease in association, respectively, with choices rejected and choices taken was apparent during the assessment of both internal and external probabilities during metacognitive decisions (two-way repeated ANOVA with the main effects of evidence and chosen task; main effect of internal/external probability, $F_{1,22}=1.06$, $p=0.31$; main effect of chosen/unchosen task, $F_{1,22}=23.0$, $p=0.001$; interaction, $F_{1,22}=1.10$, $p=0.30$). **(D)** Visual association cortex is the only area where both the internal and external probabilities were coded in a similar manner during the metacognitive judgment stage. **(E)** The evolution of the regression weights across time indexing the impact of internal probability (red at top) and external probability (blue at bottom) on neural activity in visual association cortex. (N=23, whole-brain effects family-wise error cluster corrected with $z>3.1$ and $p<0.05$; shade and error bars indicate SEM across participants; *** $p<0.001$, main effect of ANOVA).

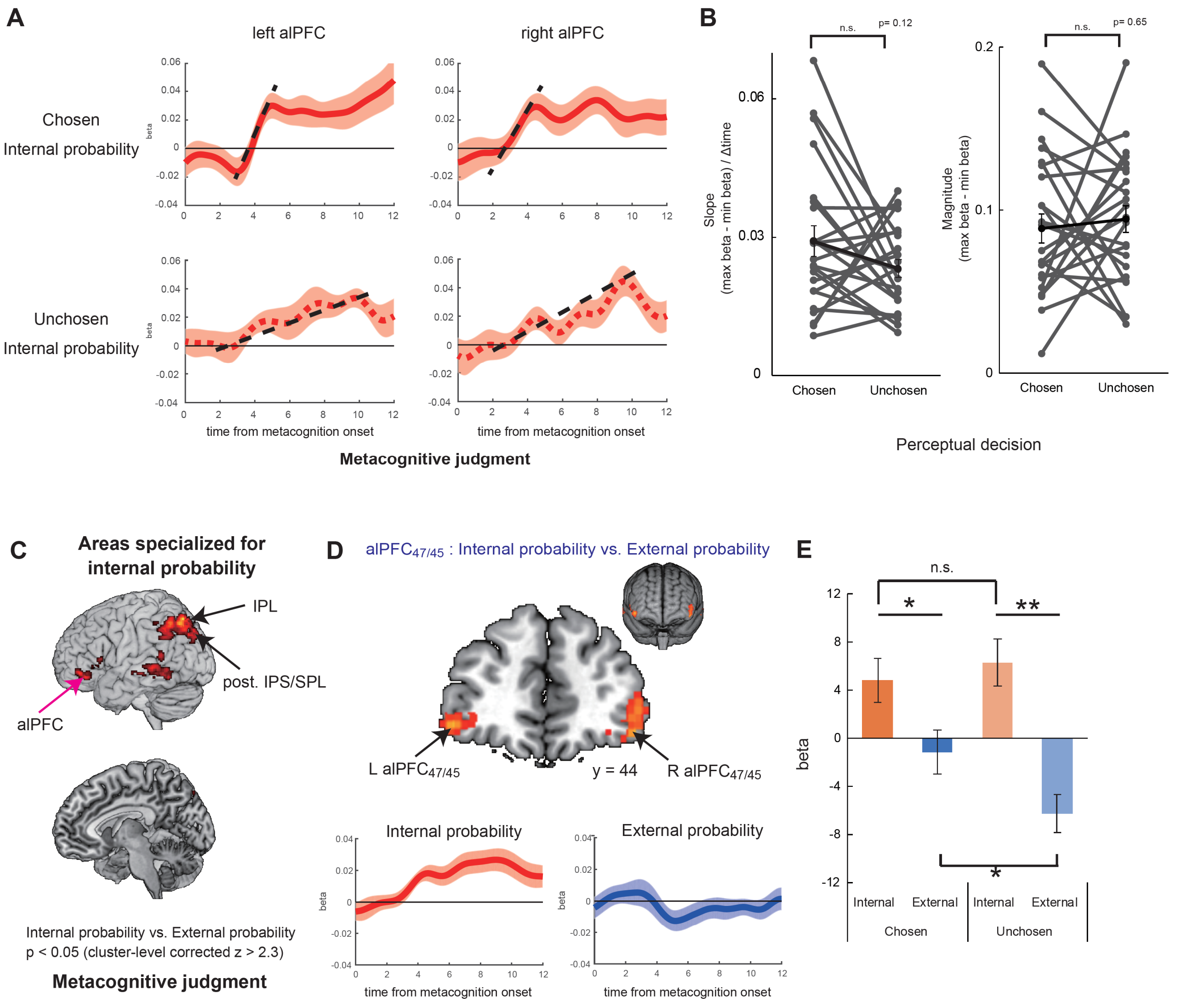

Supplemental Figure 5. Evidence accumulation of internal probability but not of external probability during metacognitive judgment in alPFC (related to Figure 5).

(A) Internal probability associated with both chosen (top) and rejected (bottom) options exerted a positive influence in a similar manner on both left and right $alPFC_{47}$. **(B)** Unlike in metacognitive judgments, there was no significant difference in slope (left) or the size of the peak signal (right) for activity related to the chosen and unchosen internal probability task options during perceptual decisions (see also Figure 5C). **(C)** Activity in $alPFC_{47/45}$ was significantly more strongly modulated by the internal probability than the external probability of chosen and unchosen options during the metacognitive judgment stage, illustrated at a liberal statistical criterion for display purpose (whole-brain effects family-wise error cluster corrected with z>2.3 and p<0.05). There was a clear region of difference within

alPFC that peaked adjacently ([x, y, z]=[-48, 44, -8], [48, 44, -12]) lying on the boundary between areas 45 and 47/12 (which we therefore refer as $alPFC_{47/45}$). **(D)** The $alPFC_{47/45}$ modulation in relation to internal as opposed to external probability is also evident in the time courses of the effects. **(E)** Unlike in other areas linked to decision making in fMRI investigations, activity modulation in $alPFC_{47/45}$ was positively modulated by the internal probability associated with both the chosen and the unchosen option. While the external probability associated with a chosen option had no impact on $alPFC_{47/45}$ activity, the external probability associated with an option that was rejected led to a decrease in $alPFC_{47/45}$ activity. This was apparent when the activity was analyzed with a two-way repeated measures ANOVA with factors of evidence type (internal/external probability) and option chosen (chosen or unchosen task); there was a significant main effect of internal/external probability ($F_{1,22}$=18.26, p=0.0003) but no main effect of chosen/unchosen task ($F_{1,22}$=0.56, p=0.46) or interaction ($F_{1,22}$= 3.30, p=0.082). (N=23, shade indicates SEM across participants).

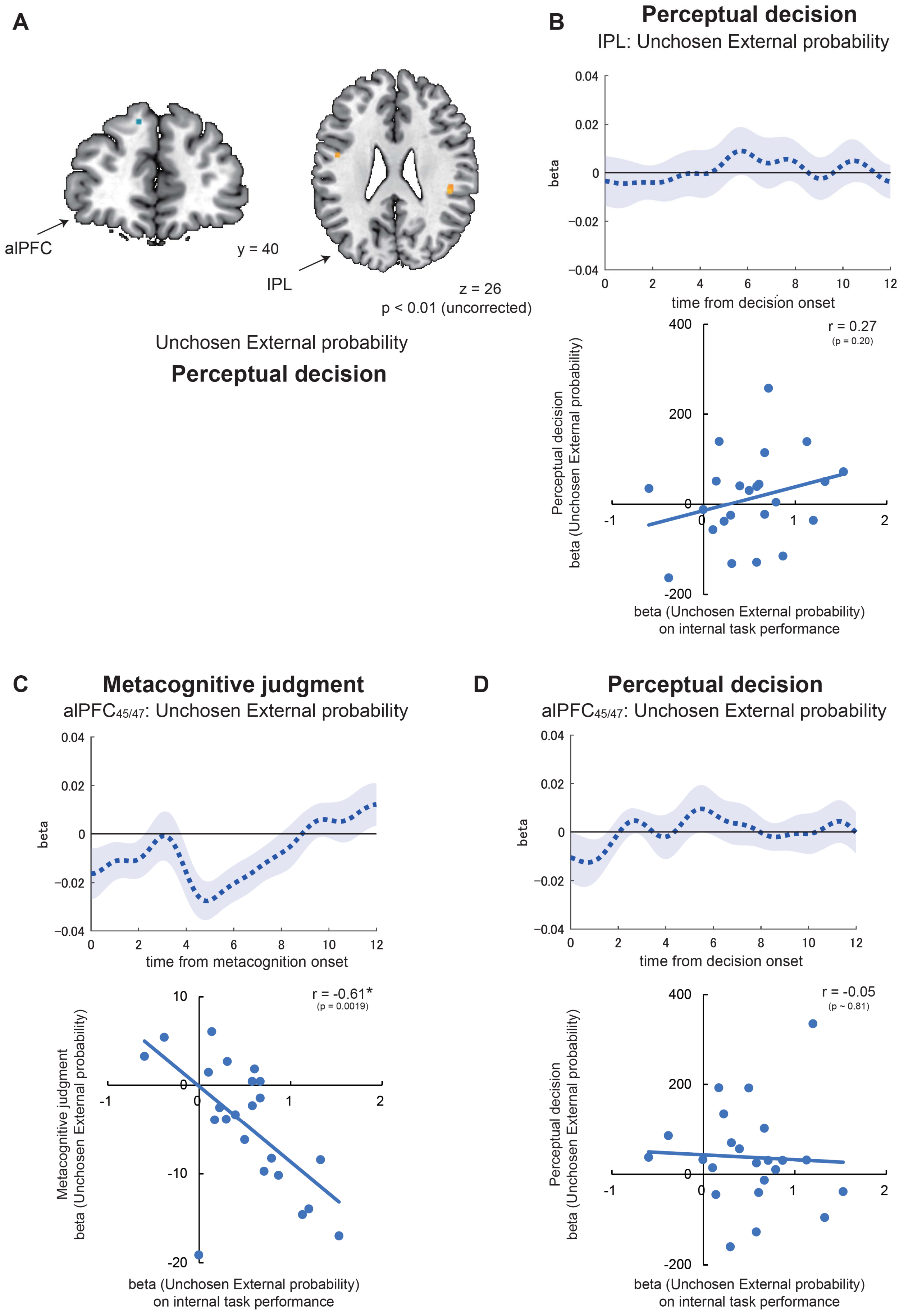
A
aIPFC
y = 40
IPL
z = 26
p < 0.01 (uncorrected)
Unchosen External probability
Perceptual decision
B
Perceptual decision
IPL: Unchosen External probability
beta
time from decision onset
r = 0.27
(p = 0.20)
Perceptual decision
beta (Unchosen External probability)
beta (Unchosen External probability)
on internal task performance
C
Metacognitive judgment
aIPFC45/47: Unchosen External probability
beta
time from metacognition onset
r = -0.61*
(p = 0.0019)
Metacognitive judgment
beta (Unchosen External probability)
beta (Unchosen External probability)
on internal task performance
D
Perceptual decision
aIPFC45/47: Unchosen External probability
beta
time from decision onset
r = -0.05
(p ~ 0.81)
Perceptual decision
beta (Unchosen External probability)
beta (Unchosen External probability)
on internal task performance

Supplemental Figure 6. Activity in alPFC during metacognitive judgment is not explained by attentional modulation as a function of the stakes of reward indicated by external probability (related to Figure 5)

(A) Activity in alPFC (Figure 5A) and IPL (attention area; Figure S3D) were not related to unchosen external probability options, which reflects the reward stakes, at the time of perceptual decision even at a very liberal statistical criterion ($p<0.01$, uncorrected). **(B)** (top) The evolution of the regression weights across time indexing the impact of unchosen external probability during perceptual decision at IPL. (bottom) The beta of IPL activity was not correlated with the influence of external probability on perceptual decision performance on the internal task (Figure 3C, left, blue) across participants ($r=0.27$, $p=0.20$). **(C)** (top) The evolution of the regression weights across time indexing the impact of unchosen external probability during metacognitive judgment at $alPFC_{47/45}$. (bottom) The beta of $alPFC_{47/45}$ activity was significantly correlated with the influence of external probability on perceptual decision performance on the internal task across participants ($r=-0.61$, $p=0.0019$). Note that the betas of BOLD and performance improvement are entirely independent. The former were measured during the metacognitive judgment stage but the latter were assessed during the perceptual decision stage. These observations suggest that alPFC also reflects external probability even if it is only in order to compare it with internal probability during prospective metacognitive judgment. **(D)** (top) The evolution of the regression weights across time indexing the impact of unchosen external probability during perceptual decision at $alPFC_{47/45}$. (bottom) The beta of $alPFC_{47/45}$ activity measured during metacognitive judgments was not significantly correlated with the influence of external probability on subsequent perceptual decisions involving the internal task across participants ($r=-0.05$, $p=0.81$).

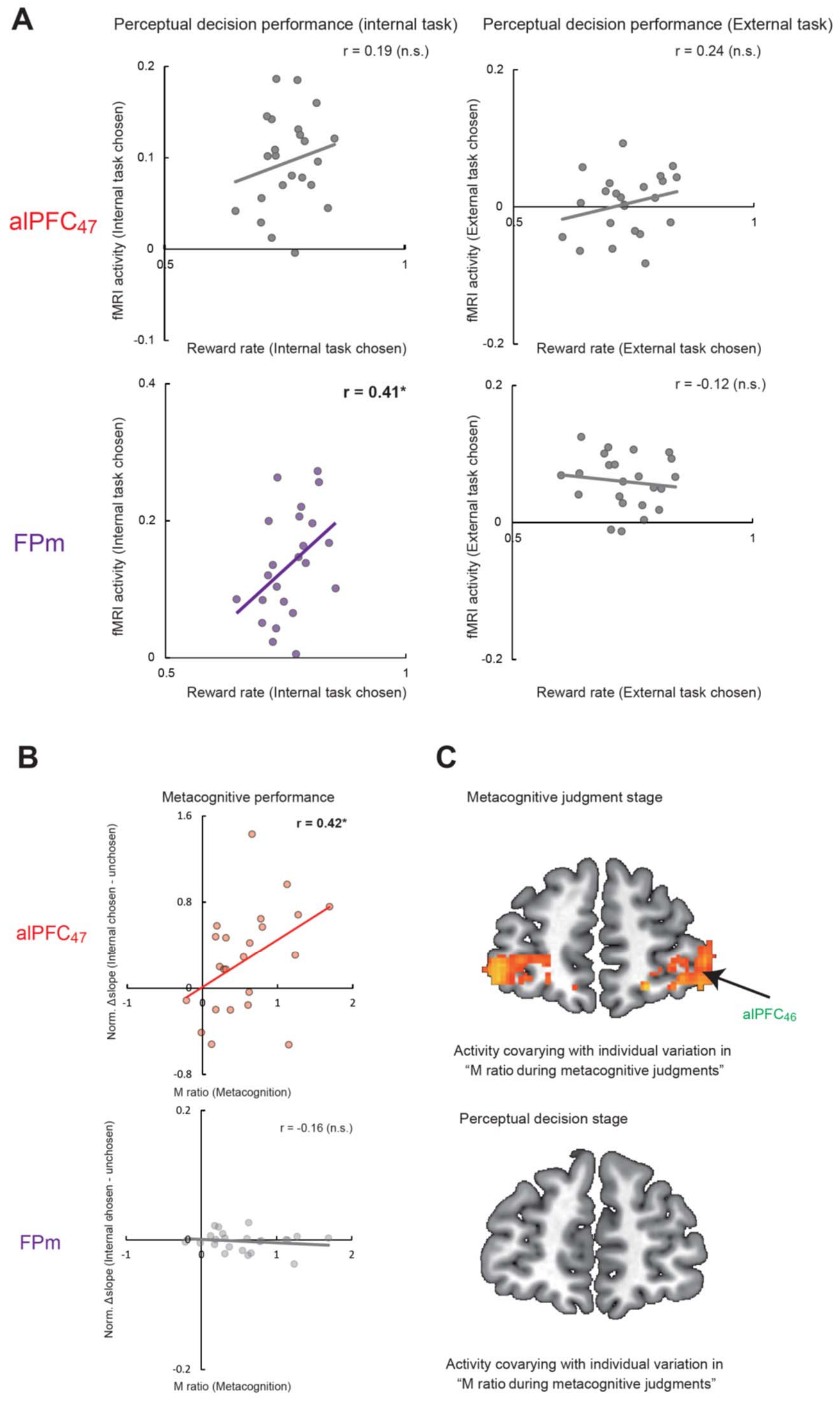

Supplemental Figure 7. Individual variation in FPm activity patterns was related to individual variation in perceptual decision making patterns in the internal probability task but not in the external probability task (related to Figure 6).

(A) FPm activity was significantly correlated with internal task performance during perceptual decision making but not with external task performance. alPFC$_{47}$ activity was correlated with neither internal nor external probability task

performance at the perceptual decision stage. **(B)** Because type-II A_{ROC} can be affected by type-I A_{ROC}, we also examined the neural correlates of metacognitive performance using the M-ratio index (meta-d' / d') (Maniscalco and Lau, 2012). M-ratio (meta-d' / d') was correlated with $alPFC_{47}$ activity ($r=0.43$, $p=0.04$) but not with FPm activity ($r=-0.17$, $p=0.43$). Once again, there was a significant difference in correlation coefficients indexing alPFC activity effects or FPm effects and individual variation in metacognitive performance (ΔFisher's $z=1.96$, $p=0.048$). **(C)** The difference in activity modulation associated with the chosen and rejected internal probability task options in $alPFC_{46}$ covaried with individual variation in metacognitive judgment accuracy as indexed by M-ratio during metacognitive judgment (top) but not during perceptual decision (bottom). (N=23; illustration shows whole-brain effects family-wise error cluster corrected with $z>2.3$ and $p<0.05$ for display purpose; *$p<0.05$).

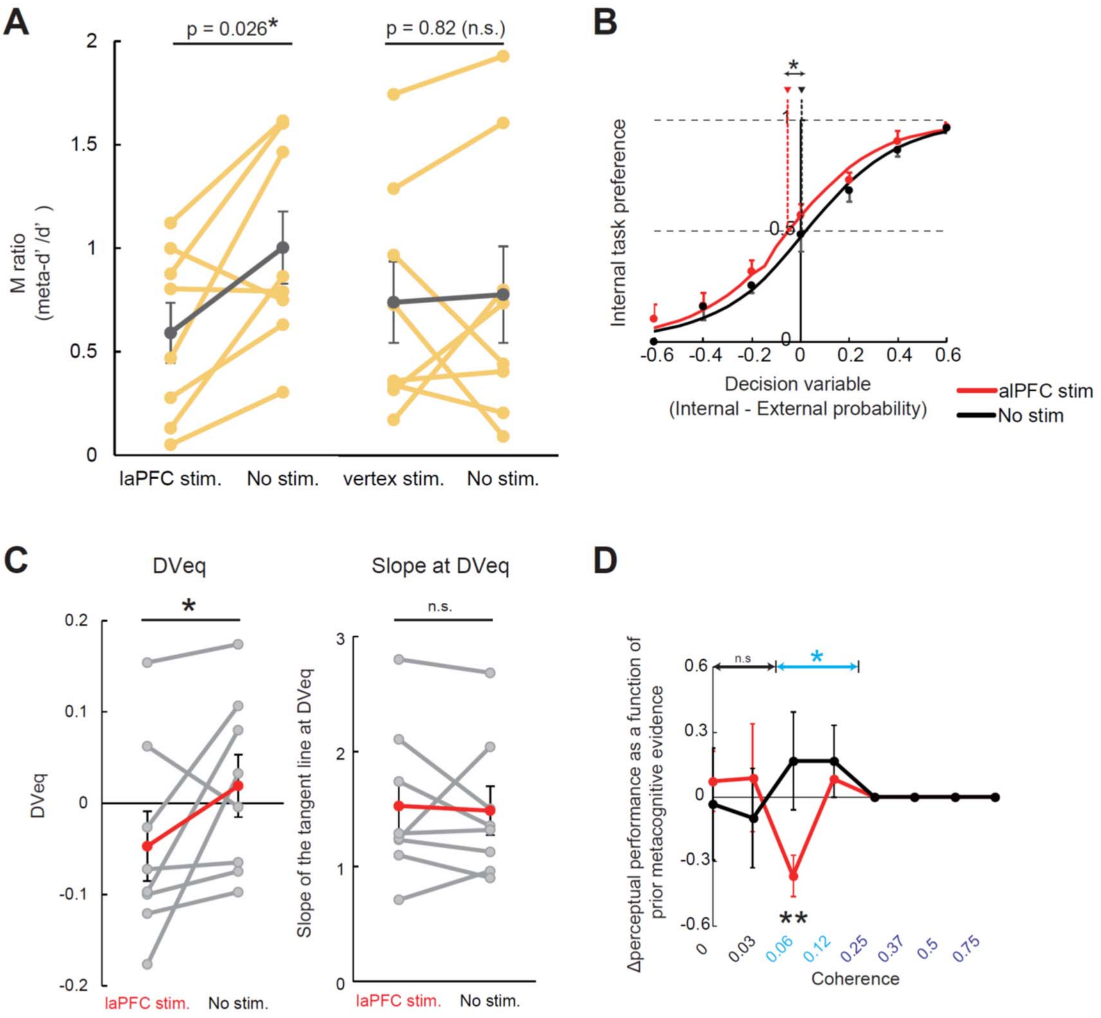

Supplemental Figure 8. Changes of preference in metacognitive choice after alPFC cTBS (related to Figure 7).

(A) Comparisons of M-ratio (meta-d'/d') between stimulation and no-stimulation for alPFC stimulation (left) and vertex stimulation (right). **(B)** Preference for choosing the internal probability task as a function of decision variable (DV). We tested whether participants' preferences for internal probability options during metacognitive judgments changed as a function of the difference between external and internal probability; this is the key decision variable (DV) that should guide metacognitive judgments (see also Figure S2B). $alPFC_{47}$ stimulation, compared to no-stimulation induced a significant bias towards choosing the internal task (change in the equilibrium point [DVeq], i.e. that is where the difference between internal and external probability is zero and the probability of choosing the internal task is 50%). **(C)** The equilibrium point (DVeq), at which the participants chose internal and external

probability options with equal frequency, significantly decreased when alPFC was stimulated; participants chose the internal option more often suggesting they overestimated their likely perceptual performance on internal options in the second perceptual decision stage ($alPFC_{47}$ stimulation, DVeq=-0.047±0.038; no stimulation, DVeq=0.019±0.034; signed-rank test between DVeq: p=0.040) (left panel). However, the sensitivity of metacognitive judgment to DV did not change (right panel; $alPFC_{47}$ stimulation, slope of the tangent line at DVeq=1.52±0.053; no stimulation, slope at DVeq=1.48±0.21; signed-rank test between DVeq: p=0.64). Red line indicates mean across participants. **(D)** Comparisons of perceptual performance improvement for Challenge trials against Inevitable trials between $alPFC_{47}$ stimulation (red) and no-stimulation (black). (N=8; error bars indicate SEM across participants; *p<0.05).
