# Supplementary material for: Identification and disruption of a neural mechanism for accumulating prospective metacognitive information prior to decision-making
Source: Neuron. 2021 Apr 21;109(8):1396–1408.e7. doi: 10.1016/j.neuron.2021.02.024 (PMC8063717; doi:10.1016/j.neuron.2021.02.024)
Supplement: Document S2. Article plus supplemental information [file mmc2.pdf]

Neuron

Article

Identification and disruption of a neural mechanism for accumulating prospective metacognitive information prior to decision-making

Highlights

- People prospectively estimate their “internal probability” of deciding correctly
- Prefrontal area, alPFC, accumulates internal probability information
- alPFC disruption impairs prospective metacognition but not perceptual decisions
- alPFC is crucial for estimating performance prior to tackling a perceptual decision

Authors

Kentaro Miyamoto, Nadescha Trudel, Kevin Kamermans, ..., Lennart Verhagen, Marco K. Wittmann, Matthew F.S. Rushworth

Correspondence

kentaro.miyamoto@psy.ox.ac.uk

In brief

Miyamoto et al. found that activity in the anterior lateral prefrontal cortex (alPFC; area 47) reflects accumulation of information for prospective metacognitive decisions and predicts prospective metacognitive performance. Disruption of the alPFC by transcranial magnetic stimulation impairs accurate estimation of the probability of success prior to tackling difficult decisions with perceptual ambiguity.

Miyamoto et al., 2021, Neuron *109*, 1396–1408
April 21, 2021 © 2021 The Author(s). Published by Elsevier Inc.
https://doi.org/10.1016/j.neuron.2021.02.024

CellPress

CellPress
OPEN ACCESS

Neuron

Article

Identification and disruption of a neural mechanism for accumulating prospective metacognitive information prior to decision-making

Kentaro Miyamoto,[1,3,4,*] Nadescha Trudel,[1,3] Kevin Kamermans,[1] Michele C. Lim,[1] Alberto Lazari,[2] Lennart Verhagen,[1] Marco K. Wittmann,[1] and Matthew F.S. Rushworth[1]

[1]Wellcome Centre for Integrative Neuroimaging (WIN), Department of Experimental Psychology, Tinsley Building, University of Oxford, Mansfield Road, Oxford OX1 3TA, UK

[2]Wellcome Centre for Integrative Neuroimaging (WIN), FMRIB, Nuffield Department of Clinical Neurosciences, University of Oxford, Oxford OX3 9DU, UK

[3]These authors contributed equally

[4]Lead contact

*Correspondence: kentaro.miyamoto@psy.ox.ac.uk

https://doi.org/10.1016/j.neuron.2021.02.024

SUMMARY

More than one type of probability must be considered when making decisions. It is as necessary to know one's chance of performing choices correctly as it is to know the chances that desired outcomes will follow choices. We refer to these two choice contingencies as internal and external probability. Neural activity across many frontal and parietal areas reflected internal and external probabilities in a similar manner during decision-making. However, neural recording and manipulation approaches suggest that one area, the anterior lateral prefrontal cortex (alPFC), is highly specialized for making prospective, metacognitive judgments on the basis of internal probability; it is essential for knowing which decisions to tackle, given its assessment of how well they will be performed. Its activity predicted prospective metacognitive judgments, and individual variation in activity predicted individual variation in metacognitive judgments. Its disruption altered metacognitive judgments, leading participants to tackle perceptual decisions they were likely to fail.

INTRODUCTION

To survive in an unpredictable world, humans and other animals monitor the potential benefits of the choices they might make. There are two factors to be considered when evaluating a choice. First, it is necessary to understand the chance that the choice will lead to the desired outcome. It is, however, equally important to know one's chances of making the choice correctly. We refer to these two probabilities as external and internal probability, respectively. For example, we might estimate our ability to drive to a new restaurant without a GPS (internal probability). This, together with the likelihood that the restaurant is open (external probability), determines our eagerness to try driving to the restaurant.

External probability reflects the fact that when a choice is taken in a given environment, the outcome may be delivered probabilistically irrespective of one's efforts. This results from indeterminacy inherent in the environment. The neural mechanisms mediating human and animal decision-making in the context of external probability have been the subject of considerable discussion (Murray and Rudebeck, 2018; Rushworth and Behrens, 2008; Tobler et al., 2009). In contrast, internal probability reflects indeterminacy relating to the ability to make a choice correctly. For example, an agent may realize that there is only a certain probability that they will make a particular choice effectively even if they are certain that a correctly made choice leads to a reward. Internal probability can be assessed by metacognitive processes, such as self-reflection, acting on representations linked to memory, perception, and cognitive performance (Fleming et al., 2010; Kiani and Shadlen, 2009; Miyamoto et al., 2017).

It is important to emphasize that internal and external probability estimates are subjective estimates or beliefs held by individual decision makers. In the case of external probability, it is well established that we might measure the frequency with which one event follows another in the environment and that we might also examine a person's or animal's subjective estimates of the likelihood. Typically, such estimates are distorted; low and high probabilities are subjectively overestimated and underestimated, respectively (Kahneman and Tversky, 1979). Arguably, the same is true for internal probability; one might similarly measure the objective frequency with which a person performs an action correctly in a given context and also the person's subjective estimate of that probability.

The aim of the current investigation was to examine subjective estimates of internal probability and to compare and contrast them with subjective estimates of external probability. On one hand, it would be natural for people to deal with these internal

 © 2021 The Author(s). Published by Elsevier Inc.
This is an open access article under the CC BY license (http://creativecommons.org/licenses/by/4.0/).

CellPress
OPEN ACCESS

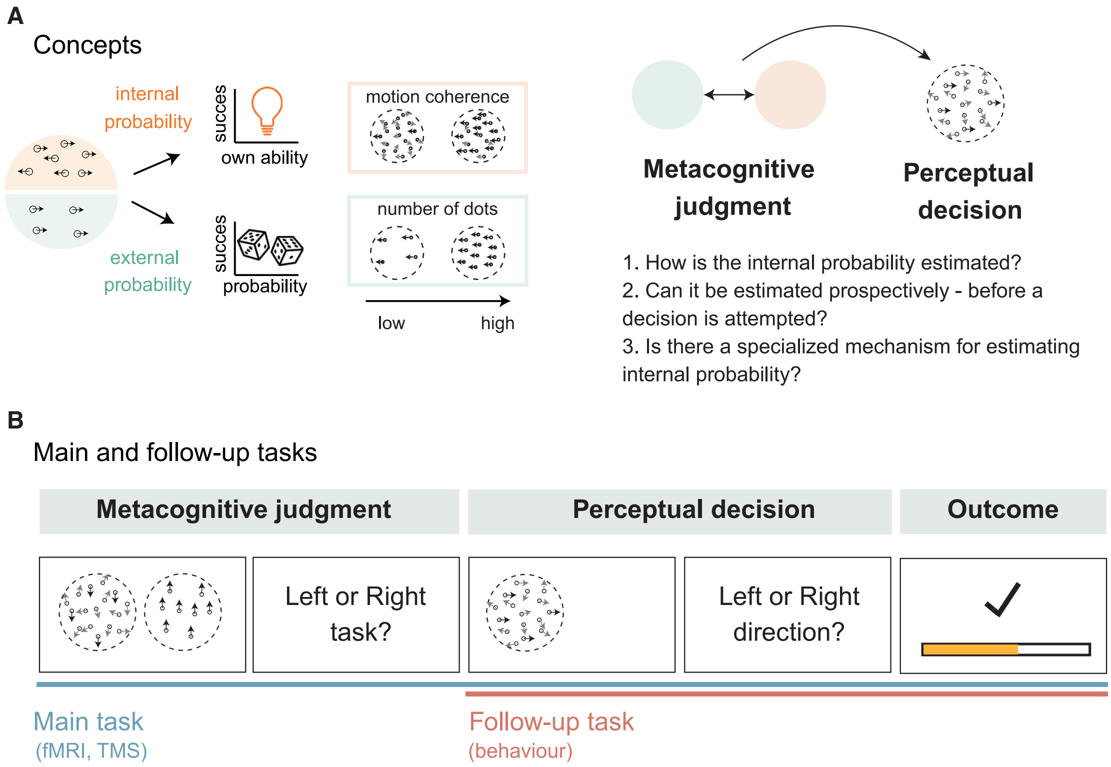

Figure 1. Prospective metacognitive comparison of internal and external probabilities: concept and task design

(A) For prospective decision-making, the internal probability—the probability that one will correctly perform a task (modulated by motion coherence of random-dot kinematogram [RDK])—and the external probability—the probability that a correctly performed task will lead to a reward (defined by the number of dots contained in the RDK)—must be considered. We examined how internal probability is estimated before the decision is attempted. We especially focused on whether there is a specialized mechanism for estimating internal probability.

(B) Example task sequence. In the main task, participants had to make a prospective decision at the metacognitive judgment stage; they decided whether to perform the internal (left RDK in the example) or external probability task (right RDK in the example) at the following perceptual decision stage. If they correctly classified the motion direction (here, left or right) of the chosen task (RDK) at the perceptual decision stage, then participants had a chance of receiving a reward. The probability of a reward was indicated by the number of dots in the RDK stimulus. The follow-up task contained no metacognitive judgment stages and only consisted of perceptual judgments made with the internal task stimuli. Importantly, the RDK stimuli in the follow-up task were the same as those that appeared during the metacognition task. The follow-up task therefore made it possible to estimate, for each participant, the probability that any given internal task option would be performed correctly in the main task. Accumulation of rewards was indicated by the yellow bar only in the main task.

and external probabilities in different ways because there is a qualitative difference in the contingencies at stake. In the case of internal probabilities, the critical contingency is between the decision maker's own action and the reward outcome. Success or failure is the result of the individual's skill. In contrast, in the case of external probability, success/failure depends on environmental stochasticity outside of the individual's control. On the other hand, despite fundamental differences in these probabilities (Figure 1A), it seems intuitive to expect people to be able to compare estimates of an internal probability of their own ability to make a choice effectively, with estimates of external probability regarding the links between choice and outcome. This seems plausible because many situations require both types of probability to be taken into consideration. Moreover, it seems likely that it is necessary to do this prospectively. Before attempting to perform the actual task, we make a judgment about whether it is wise to do so. This is the sense in which we use the word prospective. For example, even when a person ascertains that there is almost complete certainty that a choice leads to a desired outcome, it may not be advantageous to take the choice if, as a result of metacognitive inspection, the person estimates that they are unlikely to perform the choice correctly. However, despite its plausibility, whether this is possible and the mediating neural mechanism are currently unknown.

In addition to making it possible to identify activity related to a choice's internal and external probability, the task employed a two-stage design so that each trial consisted first of a prospective metacognitive judgment and then a perceptual decision. Our focus is on the initial metacognitive judgement, but to understand the specificity and generality of its mechanism, we also consider the complementary decision type—the perceptual decision.

RESULTS

Metacognitive judgments on internal and external probabilities

Participants (N = 23) performed a metacognitive probability matching task (Figure 1B) employing random dot kinematogram

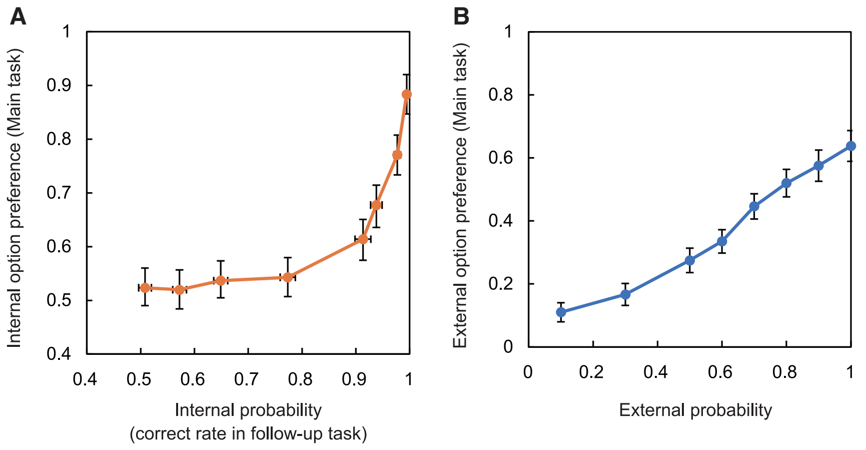

Figure 2. Independent contributions of internal and external probabilities to prospective metacognitive judgments

(A and B) The proportion of the trials where the participants chose internal (A) and external (B) probability options at the metacognitive judgment stage increased systematically with the internal and external probability, respectively (n = 23, error bars indicate SEM across participants).

(RDK) stimuli. Participants made left or right key presses depending on the direction in which most dots moved (the "coherent" dot direction). The number of dots indicated the "external probability" of receiving a reward when the correct response was made. The coherence of the synchronized dot motions determined the "internal probability" of whether a reward would be received (Figure 1A). Prior to making the perceptual decision, during a metacognitive judgment stage, participants had the opportunity to choose one of two decisions problems they wanted to attempt.

Each trial comprised a metacognitive judgment followed by a perceptual decision (Figure 1B). In the metacognitive judgment stage, participants chose one of two simultaneously presented RDK stimuli. Then, at the second stage, they performed a perceptual decision task with the RDK stimulus they had selected. The metacognitive judgment is therefore an opportunity for participants to select one of two decision tasks to perform in the second stage, and the participant's aim is to select the decision task through which they are most likely to obtain a reward. One stimulus, which might appear on the left or right of the screen in any trial, represented an internal probability decision task. It contained a full number of dots (indicating the highest external probability of reward), but the movements of the dots were ambiguous (varying between 0% and 75% coherence). This was referred to as the internal probability option because the participant had to estimate the probability that they would make the perceptual decision accurately when confronted with the same level of coherence during the second stage of the trial. Note that a reward outcome would always ensue after correct performance of the internal probability task (i.e., the external reward probability was 1).

The other stimulus represented the external probability task and contained a smaller number of dots (a number varying between 10 and 100, indicating external probabilities of reward between 0.1 and 1.0), but all dots moved in the same direction (100% coherence). It was referred to as the external probability option because participants estimated the probability that a stimulus comprising the given number of dots would lead to a reward. Judgment of the motion direction was simple. This was indicated by two observations (Figures S1A and S1B). First, an additional control experiment confirmed that participants could compare two external probability options and choose the option that would provide a reward reliably in 92.5% ± 1.5% (mean ± SEM) of trials. Second, the correct perceptual decision was made in 97.9% ± 0.7% of trials in the main task when participants chose to perform an external probability option. However, reward outcomes were still probabilistic.

In summary, at the metacognitive judgment stage, participants estimated their likely motion discrimination performance on the internal probability option and compared it with the probability of reward indicated by the external option. At the subsequent perceptual decision stage, the stimulus chosen in the metacognitive decision appeared again, but the direction of dot motion was rotated by ±90°. This change in dot motion, randomized across trials, ensured a meaningful link between the first-stage metacognitive judgment and the second-stage perceptual decision, but it prevented the participants from actually making the second-stage perceptual decision while still engaged in the prior metacognitive judgment (Figure 1B). At the second perceptual decision stage, if the participant detected the motion direction correctly, then they received reward with the probability indicated by the number of dots contained in the RDK stimulus.

At the metacognitive judgment stage, if the participant estimates their probability of successfully classifying the motion direction of the internal option at a level that exceeds the reward probability of the external probability option, then it is optimal for them to pick the internal probability option. Participants were indeed capable of making such metacognitive judgments; they changed their preferences as a function of their likely performance levels on the internal probability option. We assessed what these performance levels would be in a follow-up task that had a simpler trial structure; there were no initial metacognitive judgment stages on any trials; instead, each trial simply comprised internal probability decisions (Figure 1B). Performance on the internal decisions in the follow-up task, by which internal probability is estimated (Figure 2), is illustrated by the black curve superimposed on the summary of participants' choices of the internal probability task in the metacognitive judgment stage (Figure 3A).

For internal and external probability, the participants' preferences for a task option increased systematically with the probability that performing the task would yield a reward, suggesting that they formed beliefs regarding internal and external probabilities and used these to guide metacognitive judgments (Figure 2). The utility functions for internal and external probabilities are similar (Figures S1C and S1D; see STAR Methods for details).

CellPress
OPEN ACCESS

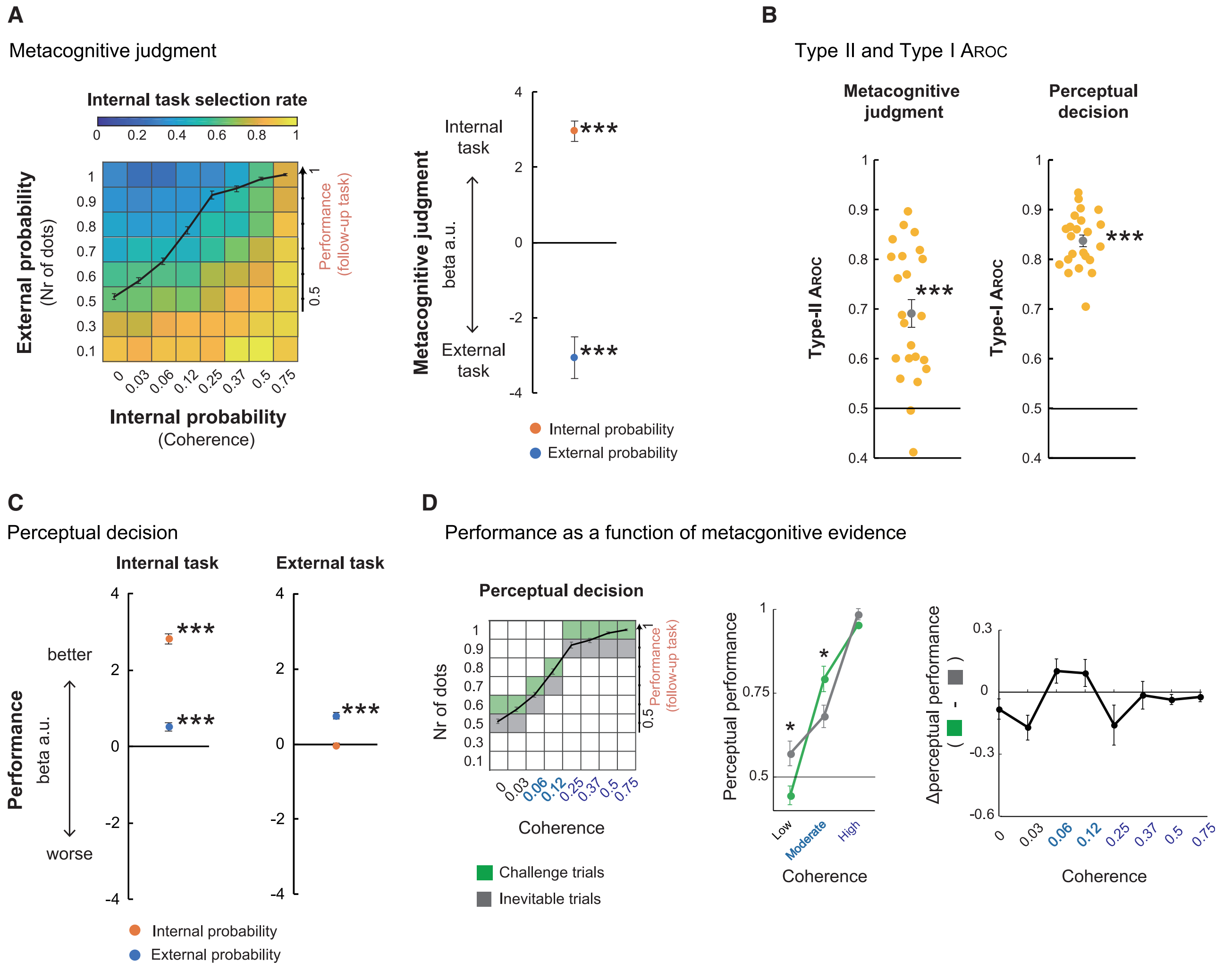

Figure 3. Prospective metacognitive performance and subsequent perceptual decision performance

(A) Left: participants' choices of the internal probability task at the metacognitive judgment stage in the main task increased when either the internal probability increased or the external probability decreased. The overlaid black line indicates performance levels in the internal probability trials during the follow-up task, binned by coherence. Right: participants selected the internal task more frequently as its coherence increased and as the probability of the external task decreased.

(B) Metacognitive judgment performance, evaluated by a type II ROC-based index (A_{ROC}), and performance of the internal task at the perceptual decision stage, evaluated by a type I A_{ROC}, were significantly greater than chance (0.5).

(C) Influence of internal and external probabilities on second-stage performance of the internal task (left) and external task (right). The performance in internal and external tasks improved with higher internal and external probability, respectively. Additionally, performance of the internal task increased when the external probability task that had been rejected was associated with a higher probability.

(D) Perceptual decision performance in the internal task at moderate coherence levels (0.06, 0.12) was higher when the internal task had been paired with an external probability option that was, on average, slightly more likely to yield a reward (challenge trials) than it was when the internal task was paired with an external probability option slightly less likely to yield a reward, on average (inevitable trials). The black line in the square plot indicates performance in the follow-up task.

N = 23 participants; *$p < 0.05$, ***$p < 0.001$, t test against chance level, Bonferroni correction when required; error bars indicate SEM across participants.

The accuracy of metacognitive decision-making is typically described by a type II receiver operating characteristic (ROC)-based index (A_{ROC}) (Maniscalco and Lau, 2012). This indicates how optimally participants selected the internal probability option (it indexes how often participants select the internal option when that is indeed the optimal choice to take and how often participants select the internal option when the external option would have been the optimal one to take). Type II A_{ROC} is partly a function of probability distortions that occur during subjective estimation; the subjective distortion of the utility functions for external and internal probabilities that are described predict type II A_{ROC} in similar ways (Figures S1E and S1F). Type II

A_{ROC} was significant across participants ($t_{22} = 6.08$, $p = 3.9 \times 10^{-6}$, t test against chance level; Figure 3B, left panel; Figure S2A).

Similarly, a type I A_{ROC} for perceptual decision performance can be constructed as an index of accuracy when participants take the internal option in the perceptual decision phase of each trial. It was also significant across participants ($t_{22} = 28.9$, $p = 5.3 \times 10^{-19}$, t test against chance level; Figure 3B, right panel). Participants' preferences for the internal option increased when the internal probability increased with respect to the external probability ($\beta_{slope} = 2.49 \pm 0.22$ [mean ± SEM], $t_{22} = 8.61$, $p = 1.6 \times 10^{-8}$, t test against zero; Figure S2B). Logistic multiple regression analyses revealed that participants' preferences for the internal option increased in proportion to its motion coherence (which, in turn, determined internal probability; see STAR Methods for conversion of coherence into internal probability; $\beta_{internal} = 2.96 \pm 0.26$ (mean ± SEM), $t_{22} = 11.03$, $p = 9.8 \times 10^{-11}$, t test against zero) and decreased in proportion to the external probability of the other option ($\beta_{external} = -3.07 \pm 0.55$, $t_{22} = -5.49$, $p = 1.5 \times 10^{-5}$; Figure 3A). The regression analysis shows the general effect of varying levels of external and internal probabilities on participants' preferences but does not allow claims to be made about whether the preferences were optimal. A further indication that internal evidence was treated in a different manner from external information was that feedback about the success or failure of internal probability options exerted an influence over subsequent choices not seen after external probability options were taken (Figures S2C and S2D).

In addition, we examined the relationship between initial metacognitive judgments and subsequent perceptual decisions (Figures 3C, S2C, and S2E). As predicted, choosing an internal probability task with high motion coherence during the metacognitive stage led to better performance during the perceptual stage with the same task ($\beta_{internal} = 2.81 \pm 0.13$, $t_{22} = 21.02$, $p = 4.6 \times 10^{-16}$, t test against zero; Figure 3C, left panel). Intriguingly, however, perceptual decisions for internal probability options also became better when the reward probability linked to the rejected external option in the earlier metacognitive judgment stage was higher ($\beta_{external} = 0.51 \pm 0.10$, $t_{22} = 4.79$, $p = 8.5 \times 10^{-15}$, t test against zero; Figure 3C, left panel). This suggests that participants make fine-grained metacognitive judgments about precisely which internal probability tasks they should tackle; they use their metacognitive judgment to opt for the internal probability task when they are likely to be able to perform it. This was supported by the finding that participants more often chose internal probability options when they correctly classified the motion direction in the follow-up task (Figures S2F and S2G). Such an effect will arise when metacognition allows participants to have a finely calibrated sense of which internal options they should attempt to tackle because the RDK stimulus on a given trial made it likely that subsequent decisions about such stimuli would be performed correctly (e.g., some variation in the distribution of coherently moving dots or some stochastic feature of the dots in a given trial that was repeated at the metacognitive and perceptual decision stages). In contrast, for external probability options, as participants almost perfectly classified the motion direction (proportion of trials performed correctly: 97.9% ± 0.7% [mean ± SEM]), by design, "performance" is a function of the probabilistic reward outcome indicated by the number of dots. Not surprisingly, the performance on external probability options at the second perceptual decision stage was not predicted by the coherence of the internal probability option that had been rejected in the immediately preceding metacognitive phase ($\beta_{internal} = -0.26 \pm 0.42$, $t_{22} = -0.60$, $p = 0.55$, t test against zero; Figure 3C, right panel). As expected, external task performance simply increased with higher external probability ($\beta_{external} = 0.75 \pm 0.078$, $t_{22} = 9.67$, $p = 2.2 \times 10^{-9}$, t test against zero; Figure 3C, right panel).

If metacognitive judgments gave participants the opportunity to select internal perceptual decisions they realized they were likely to succeed in performing, then this should be apparent if we compare the rate at which participants performed internal decisions on two types of trials we refer to as "challenge" and "inevitable" trials. Challenge trials were ones on which the external probability option was, on average, linked to a higher probability of reward than the internal probability option (green squares in Figure 3D). Inevitable trials were ones on which the external probability option was, on average, linked to a lower probability of reward than the internal probability option (gray squares in Figure 3D). In challenge trials, on average, there ought to be a higher probability of reward for taking the external option as opposed to the internal option. Participants, however, did indeed benefit from taking the internal option in such challenge trials when the internal option was associated with a moderate level of coherence, where the perceptual performance was significantly different from chance level (50%) but did not yet approach 100% (ceiling effect) (Figure 3D, center). Perceptual decision performance for moderate coherence (coherence levels: 0.06, 0.12) improved when participants purposely rejected higher external probability options in challenge trials compared with when they chose internal probability options in inevitable trials when a lower external probability was offered. This suggests that the participants used their metacognitive assessment of their likely performance levels in a prospective and adaptive manner. Floor and ceiling effects on performance prevent the same phenomenon appearing when the internal probabilities, or coherences, were very low or very high, respectively (Figure 2D, right; see also Figures S2H and S2I for a comparison between challenge and inevitable trials that uses a wider window of trials to calculate internal task performance).

Neural activity during metacognitive judgment and perceptual decision: hypotheses

In the behavioral analyses, we have demonstrated that participants evaluated internal and external probabilities. However, some features of the results (for example, the existence of challenge trials) suggest specialization in the mechanism for internal probability estimation. However, similarities in distortion patterns and the ability to compare internal and external probabilities suggest a common mechanism. We therefore turn to analyses of neural data to examine whether internal and external probabilities are encoded similarly in the brain. We tested whether (1) internal and external probabilities coactivate the same brain region or (2) internal probability has a particular neural substrate (Figure 4). Because we found evidence of specialized processing of internal probabilities, we examined an additional hypothesis: (3) does the brain area critical for

CellPress
OPEN ACCESS

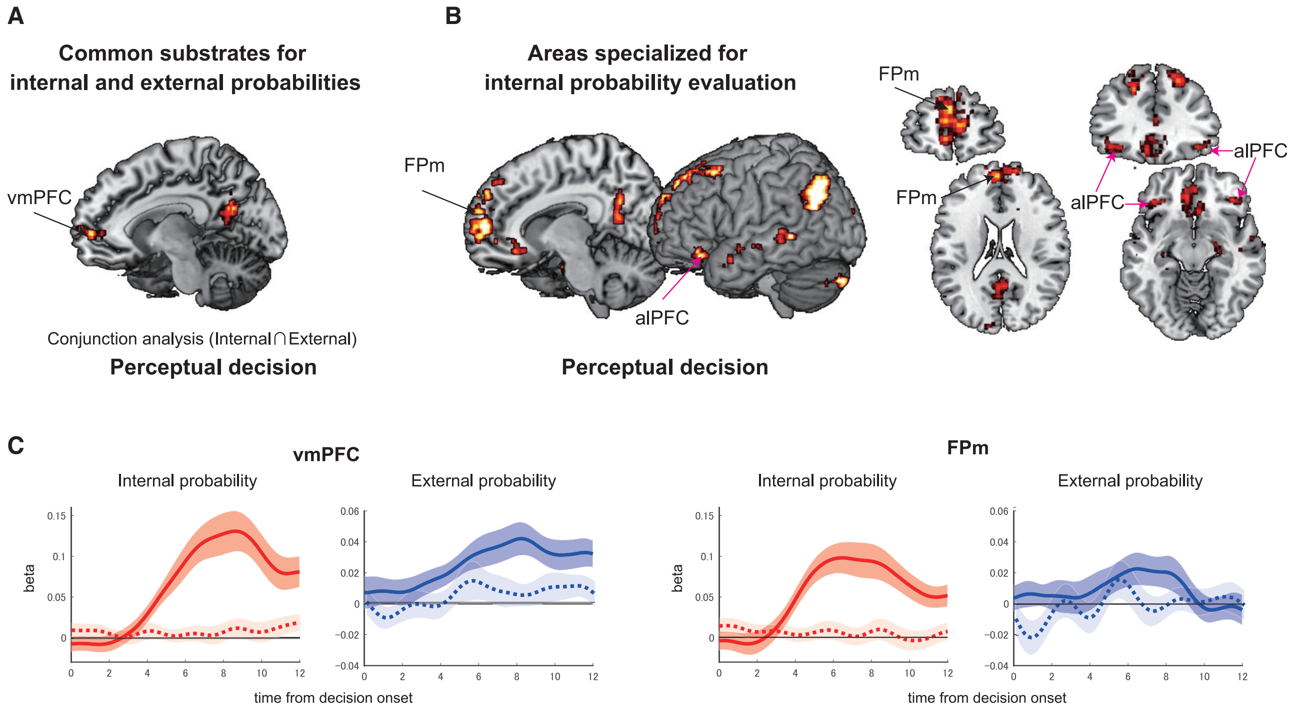

Figure 4. Perceptual decision-making: common and specific substrates encoding internal and external evidence

(A) Activity in the vmPFC and posterior cingulate cortex reflected the probability of a reward associated with the chosen action—the evidence of making the chosen action—regardless of whether it was an internal or external probability.

(B) Activities in the FPm and alPFC were related specifically to evaluation of chosen internal probability options during perceptual decisions.

(C) Evolution of regression weights across time, indexing the effect of internal probability (red at the left of each area; solid line, chosen internal probability; dotted line, unchosen internal probability) and external probability (blue at the right of each area; solid line, chosen external probability; dotted line, unchosen external probability) on neural activity are illustrated for two example areas: the vmPFC and FPm.

N = 23; whole-brain effects family-wise error cluster corrected with $z > 3.1$ and $p < 0.05$; shade indicates SEM across participants.

prospective metacognition encode the internal probability when the internal probability option is chosen and when it remains unchosen during the metacognitive judgment stage (Figure 5)? Does individual variation in activity also predict individual variation in metacognitive performance (Figure 6)? The focus of the third hypothesis is on the anterior lateral prefrontal cortex (alPFC), which has a unique role in accumulating evidence regarding internal probabilities during prospective metacognitive judgments. We consider the alPFC later, but to appreciate its specialized function, first we consider areas in which patterns of activity are consistent with the first or second hypotheses.

Common neural substrates for evaluating internal and external probabilities

To test the first hypothesis, that there are common neural substrates for internal and external probability processing, we sought brain areas in which activity arose in a similar way during evaluation of both types of probabilities during the final perceptual decision stage of each trial (Figure 1B). We employed a whole-brain parametric general linear model (GLM) (fMRI-GLM1; STAR Methods, quantification and statistical analysis; Figures S3A and S3B). First we identified activity that covaried with internal probability or external probability at the perceptual decision stage of each trial. A conjunction analysis across the two contrasts showed that activity covaried in a similar way with both probabilities in several areas ($p < 0.05$, cluster-level corrected [$z > 3.1$]; Figure 4A), including the ventromedial prefrontal cortex (vmPFC; areas 10 and 11). Adjacent regions have been linked to encoding of subjective values as a function of expected reward magnitude or probability, suggesting a domain-general role in evidence accumulation (Boorman et al., 2009; De Martino et al., 2013; Fouragnan et al., 2019; Hunt et al., 2012; Kolling et al., 2016; Papageorgiou et al., 2017; Trudel et al., 2021; Wunderlich et al., 2012). Activity in some cortical areas known to contribute to perceptual decision-making, including the frontal eye field (area 8A) and intraparietal sulcus (IPS) (Gold and Shadlen, 2007; Hanks and Summerfield, 2017; Kim and Shadlen, 1999), also covaried with external and internal probability (Figures S3C and S3D). Importantly, the vmPFC was not active as a function of internal or external evidence during the metacognitive judgment stages (Figure S3E).

Next, we carried out an analogous analysis of the metacognitive judgment phase. It identified areas with activity that varied in a similar way as a function of internal and external probability. Activity in dorsal anterior cingulate cortex (dACC) covaried as a function of the differences in evidence between the chosen and unchosen options when internal or external probability was at stake ($p < 0.05$, cluster-level corrected

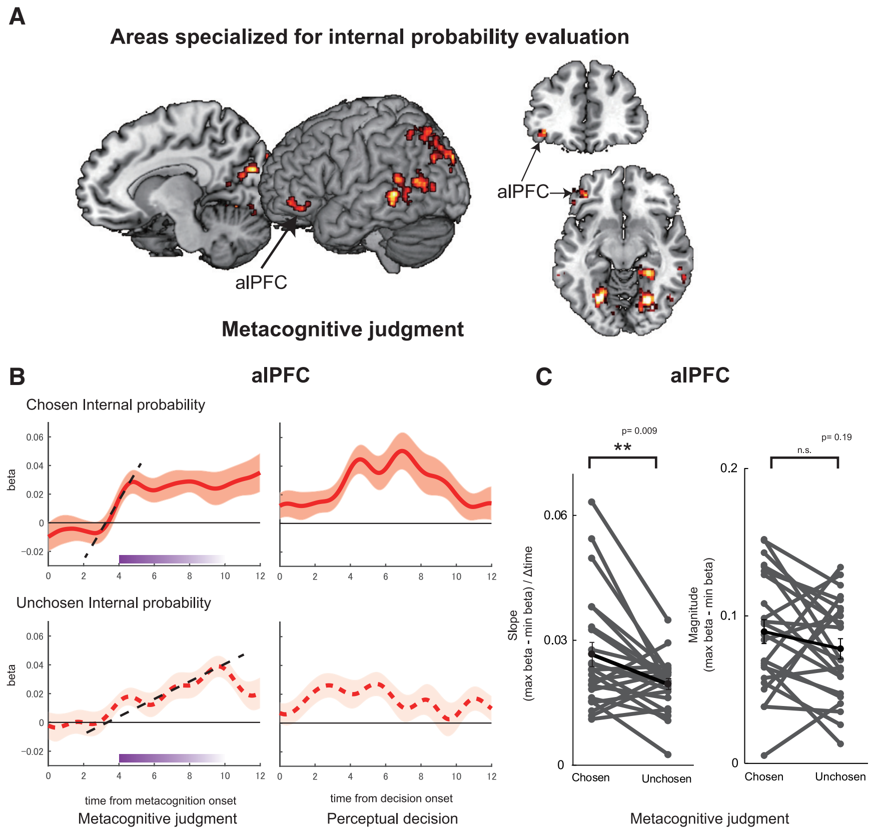

Figure 5. Specialization for internal evidence accumulation during metacognitive judgment in the alPFC

(A) Activity in the alPFC$_{47}$ was modulated significantly by the internal probability of chosen and unchosen options during the metacognitive judgment stage.

(B) Although the internal probability associated with chosen (top) and rejected (bottom) options exerted a positive influence on the alPFC$_{47}$, the modulation in relation to the option that was chosen was faster (i.e., a steeper slope). This is consistent with a faster process of accumulation of evidence concerning internal probability for an option that was ultimately chosen than for an option that was ultimately rejected. The difference was significant during the metacognitive judgment stage (left) but not during the subsequent perceptual decision stage (right). The purple line at the bottom indicates onset of the perceptual decision between 4 s and 10 s after onset of metacognitive judgment.

(C) The difference in slope for chosen and unchosen internal probability (left) was significant during metacognitive judgments, but there was no difference in peak signal (right). The tick line indicates mean across participants.

N = 23; whole-brain effects family-wise error cluster corrected with $z > 3.1$ and $p < 0.05$; shade and error bars indicate SEM across participants; **$p < 0.01$, paired t test, Bonferroni correction when required.

[$z > 3.1$]; Figure S4A). In both cases, dACC activity positively reflected reward probability (in relation to internal probability or external probability) linked to the unchosen option; if participants picked the internal option, then activity in dACC was modulated positively by evidence of the external option they had rejected, and, vice versa, it positively coded the internal probability when the external option was chosen (Figures S4B and S4C). In summary, dACC activity reflected the relative reward probability associated with the alternative choices participants did not take in the current trial but that they might take on a future occasion (Boorman et al., 2011, 2013; Fouragnan et al., 2019; Kolling et al., 2012, 2016, 2018; Meder et al., 2017). This suggests a key role of dACC in weighing up internal and external prospects of task success.

The medial frontopolar cortex is specialized for coding internal evidence during perceptual decision-making

To search for neural activity linked selectively to internal probability estimation (second hypothesis), we sought brain activity modulated more significantly by internal probability during perceptual decision-making ($p < 0.05$, cluster-level corrected [$z > 3.1$]; Figure 4B, left). The activation specific to internal probability was most prominent in the medial frontopolar area (FPm) (Neubert et al., 2014) ([x, y, z] = [−2, 56, 22]; Figure 4B, right). The activation extended to the adjacent vmPFC/medial orbitofrontal cortex (mOFC) and lateral frontopolar cortex (FPl) (Figure 4B, left). In contrast to the vmPFC (Figure 4C, left), which was modulated by chosen internal and external probabilities, the FPm was responsive only to chosen internal probability (Figure 4C, right). Neither area was modulated by unchosen probabilities. Similar effects were found in the alPFC and dorsomedial area 9; regions linked previously to metacognitive decision-making (Figure 4B, right; Fleming et al., 2010, 2012; Wittmann et al., 2016b). However, among all of these areas, only the alPFC responded differently as a function of internal probability during the preceding metacognitive judgment stage (Figure 5A), and we focus on the alPFC in the next section.

Metacognitive evaluation and matching of internal and external evidence in the alPFC

Because we found that internal probability is processed selectively in the FPm and alPFC, we tested the third hypothesis that, if a brain area critical for prospective metacognition exists, then it should encode chosen and unchosen internal probabilities because it is involved in evaluating them both regardless of whether the internal option is ultimately chosen or remains unchosen. Moreover, even when the alPFC is specialized for encoding internal probability, external probability should have some effect on its activity even if it is an effect that is different in nature, if internal probabilities are to be compared with external probability during metacognitive judgment. Activity in the left alPFC covaries with chosen and unchosen internal probabilities ([x, y, z] = [−38, 34, −10]; $p < 0.05$, cluster-level corrected [$z > 3.1$]; Figure 5A). The peak was in area 47 (Mackey and Petrides, 2010; Neubert et al., 2015; Petrides and Pandya, 2002), and so it is referred to as alPFC$_{47}$.

CellPress
OPEN ACCESS

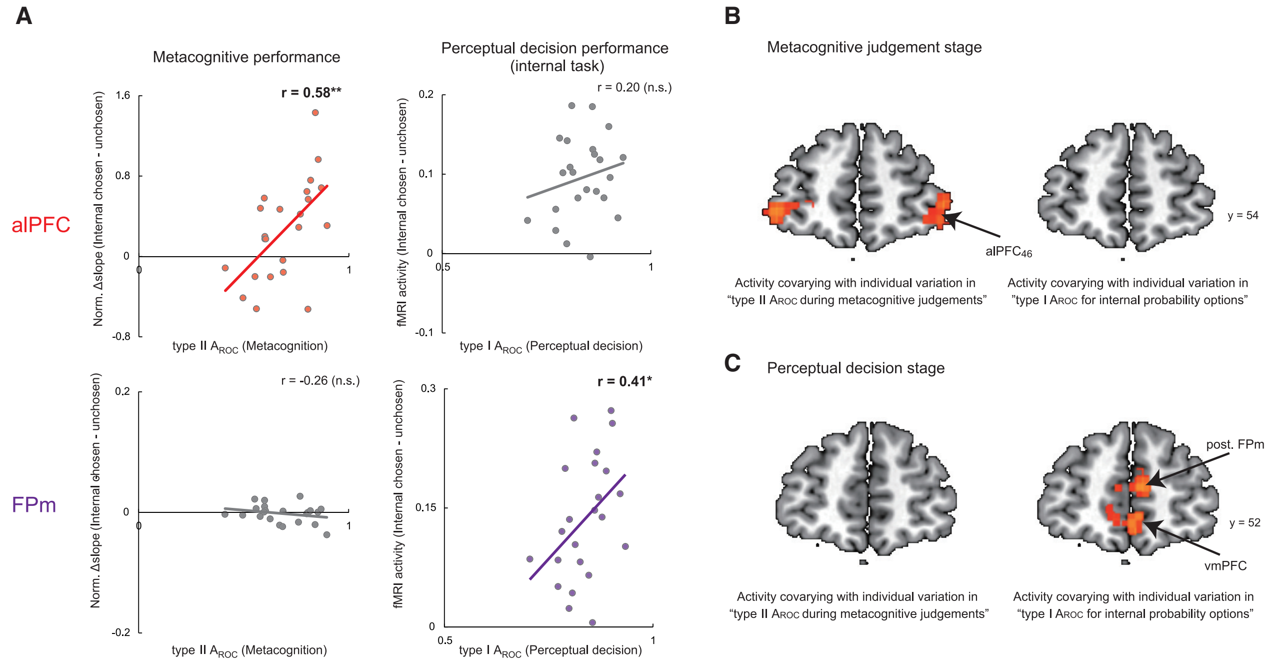

Figure 6. Individual variations in the alPFC and FPm were related to individual variations, respectively, in metacognitive and perceptual decision-making

(A) There was a correlation between type II A_{ROC} (Figure 3B, left) and alPFC$_{47}$ (Figure 5A) activity at the metacognitive judgment stage and a correlation between type I A_{ROC} (Figure 3B, right) and FPm (Figure 4B) at the perceptual decision stage.

(B) The difference in activity modulation associated with the chosen and rejected internal probability task options in the alPFC$_{46}$, but not in the FPm/vmPFC, covaried with individual variation in metacognitive judgment accuracy, as indexed by type II A_{ROC}.

(C) Individual variation in activity associated with the chosen and rejected internal probability task in the posterior FPm and vmPFC, but not in the alPFC$_{46}$, was associated with individual variation in type I A_{ROC} during perceptual decision.

N = 23; the illustration shows whole-brain effects family-wise error cluster corrected with z > 2.3 and p < 0.05 for display purposes; *p < 0.05, **p < 0.01.

Although activity in the alPFC$_{47}$ increased with internal probability when the internal option was chosen and when it was rejected, alPFC$_{47}$ activity differed in another way, in terms of its timing, as a function of whether the internal option was to be chosen or rejected (Figures 5B and S5A). During the initial metacognitive judgment stage, alPFC$_{47}$ activity increased more quickly (the slope of the signal increase was steeper) as a function of the internal probability when the option was chosen than when it was rejected (paired t test, t_{22} = 2.81, p = 0.0099; Wilcoxon's sign rank test, p = 0.011; Figure 5C, left panel). The slope differences were not explained by differences in maximum peaks; these were comparable regardless of whether the internal probability option was chosen or unchosen (paired t test between maximum beta weights for chosen versus unchosen: $t_{22\ =}$ 1.32, p = 0.19; Wilcoxon's sign rank test, p = 0.19; Figure 5C, right panel).

The importance of the alPFC$_{47}$ in metacognitive judgments was underlined by the fact that activity related to the internal probability considered in the metacognitive judgment stage continued into the perceptual decision stage of the task even when the internal option was not chosen by the participant and was now irrelevant to the perceptual decision participants made. However, during the perceptual decision, neither the peak signal nor the slope of activity change associated with internal probability differed as a function of whether the internal option probability was being considered or whether it had already been rejected (slope, t_{22} = 1.59, p = 0.12; maximum beta, t_{22} = −0.45, p = 0.65; Figure S5B). This feature of alPFC$_{47}$ during the perceptual decision contrasted with FPm, which was active in response to chosen internal probability but not rejected internal probability (dotted trace, Figure 4C).

When we examined activity near the alPFC peak, we found that it carried information significantly more strongly about internal as opposed to external evidence during metacognitive judgments (20-mm radius volume of interest analysis centered on the alPFC peak described above ([x, y, z] = [−38, 34, −10]; p < 0.05, cluster-level corrected [z > 3.1]; Figures S5C and S5D). In summary, the alPFC selectively represented internal probability. Moreover, the manner in which it coded internal probabilities associated with choices taken and rejected differed to the coding scheme in other frontal and parietal areas (Figures 4, S3C–S3E, and S6), dACC (Figures S4A and S4B), or other areas in which evidence accumulation and decision-making processes have been studied in the past.

Contrasting function of the FPm and alPFC during perceptual decision-making and prospective metacognitive judgment

We quantified the contributions of the two prefrontal areas identified by fMRI-GLM1 (Figures 4 and 5) to task performance

(Figure 6A). alPFC$_{47}$ activity (Figure 5A) was modulated by internal evidence accumulation (there were differences in effect slopes reflecting chosen and unchosen internal probability; Figure 5C), and individual variation in these effects was correlated with metacognitive performance (type II A_{ROC}) during metacognitive decisions ($r = 0.58$, $p = 0.0035$), but alPFC$_{47}$ activity related to chosen internal evidence was not correlated with variation in internal task performance (type I A_{ROC}) during perceptual decisions ($r = 0.20$, $p = 0.35$). In contrast, FPm activity (Figure 4B) exhibited complementary characteristics; variation in its activity was correlated with internal task performance during perceptual decisions ($r = 0.41$, $p = 0.048$) but not with metacognitive performance ($r = -0.26$, $p = 0.21$). Neither alPFC$_{47}$ nor FPm activity was correlated with external task performance during perceptual decisions (alPFC$_{47}$, $r = 0.24$, $p = 0.25$; FPm, $r = -0.12$, $p = 0.56$; Figure S7A). During metacognitive judgments, the correlation between individual variation in alPFC$_{47}$ effects and individual variation in metacognitive performance was significantly greater than between FPm effects and metacognitive performance (ΔFisher's $z = 2.93$, $p = 0.0033$). On the other hand, during the subsequent perceptual decision, although the correlation between individual variation in FPm effects and individual variation in perceptual decision-making was numerically greater than in the alPFC, the difference in the strength of correlations did not reach statistical significance (ΔFisher's $z = 0.73$, $p = 0.46$). The contrasting pattern therefore suggests an independent alPFC mechanism for evaluating the strength of internal evidence during second-level metacognitive judgments with a comparatively little role in first-level perceptual decisions. We confirmed this conclusion by using the M-ratio index (meta-d′/d′) (Maniscalco and Lau, 2012; STAR Methods), a metacognitive sensitivity measure that is not biased by possible interactions between type II A_{ROC} and type I A_{ROC} (Figure S7B).

We also searched across the whole brain for any activity predicting variation in the sensitivity of metacognitive judgment across participants by employing an analysis of covariance (ANCOVA) (STAR Methods, fMRI-GLM2: covariate analysis at metacognitive judgment stage and fMRI-GLM3: covariate analysis at perceptual decision stage). In the vicinity of the alPFC$_{47}$ and FPm (20-mm radius centered on either area), individual variation in activity of the alPFC close to area 46 (Petrides and Pandya, 1999) was correlated with individual variation in metacognitive accuracy (type II A_{ROC}) (Figure 6B), whereas individual variation in FPm and vmPFC (area 11 m) (Neubert et al., 2014) activity was correlated with individual variation in internal task performance (type I A_{ROC}) (Figures 6C and S7C).

Causal evidence of the contribution of the alPFC to metacognitive judgment

Finally, to evaluate the causal role of the alPFC in prospective metacognitive judgment, we disrupted alPFC$_{47}$ activity with continuous theta-burst transcranial magnetic stimulation (cTBS) and examined the effect on metacognitive performance (Figure 7A). We targeted the left alPFC$_{47}$, in which the speed of activity accumulation is different for chosen and unchosen internal probabilities (Figures 5B and S5A) and where this difference predicted metacognitive performance (Figure 6A). Behavioral data in the cTBS experiment (experiment 2; Figure 7) were collected from different participants than those participating in the fMRI experiment (experiment 1; Figures 2, 3, 4, 5, and 6).

Targeted disruption of the alPFC$_{47}$ altered patterns of preference for internal or external probability options during the metacognitive judgment (Figure 7B). The change was quantified by comparing type II and type I A_{ROC} after alPFC$_{47}$ stimulation and during the no-stimulation baseline. alPFC$_{47}$ cTBS exerted a differential influence on type II and type I A_{ROC} (two-way repeated ANOVA with the main effects of A_{ROC} and stimulation; main effect of type II/I, $F_{1,7} = 3.84$, $p = 0.090$; main effect of alPFC$_{47}$ stimulation/baseline, $F_{1,7} = 2.14$, $p = 0.18$; interaction, $F_{1,7} = 7.32$, $p = 0.030$; Figure 7C, left). Metacognitive judgment performance quantified by type II A_{ROC} was impaired significantly (main effect of alPFC$_{47}$ stimulation/baseline, $F_{1,14} = 8.70$, $p = 0.010$), whereas perceptual decision performance quantified by type I A_{ROC} did not change (main effect of alPFC$_{47}$ stimulation/baseline, $F_{1,14} = 0.33$, $p = 0.57$). In contrast, no similar effect was observed when a parallel analysis was conducted to examine the effect of stimulation applied to a control site, the vertex, in the same participants (two-way repeated ANOVA with the main effects of A_{ROC} and stimulation; main effect of type II/I, $F_{1,7} = 3.71$, $p = 0.095$; main effect of vertex stimulation/baseline, $F_{1,7} = 0.43$, $p = 0.52$; interaction, $F_{1,7} = 0.01$, $p = 0.97$; Figure 7C, right). Neither metacognitive judgment performance, quantified by type II A_{ROC}, nor perceptual decision performance, quantified by type I A_{ROC}, changed after vertex cTBS (main effect of vertex stimulation/baseline for type II A_{ROC}, $F_{1,14} = 2.97$, $p = 0.10$; for type I A_{ROC}, $F_{1,14} = 3.08$, $p = 0.10$). The difference in stimulation effects cannot be attributed to any aspect of the ordering of the tests, which were counterbalanced across participants; some participants participated in the alPFC experiment first and some in the vertex control experiment first; moreover, in each experiment, some participants underwent the stimulation condition first or the control non-stimulation condition first. The impairment of metacognitive performance by alPFC stimulation was reproduced when the M-ratio (meta-d′/d′) was considered ($t_7 = 2.81$, $p = 0.026$; Figure S8A). This testifies to the specificity of the alPFC effect even after controlling for any possible confounding influence of different levels of perceptual performance. In summary, the pattern of behavioral change suggests that alPFC$_{47}$ is essential for prospective metacognitive judgment rather than perceptual decisions (Figures S8B and S8C illustrate changes in preference for choosing the internal task option with alPFC cTBS).

If metacognitive judgment is altered, then this will change which decisions participants tackle at the subsequent perceptual decision stage. We investigated whether it led participants to tackle internal probability options for which they were unlikely to make correct decisions by comparing challenge with inevitable trials (analysis depicted in Figure 3D). First we showed that prospective judgments were again beneficial, particularly at moderate dot coherence levels (such as 0.06 and 0.12); as in the previous experiment, performance in challenge trials was higher than in inevitable trials at such moderate coherence levels (compare the gray and green lines in the right panels of Figure 7D). The difference between the gray and green lines is also illustrated by the black line in Figure S8D, which is above zero for coherence levels such as 0.06 and 0.12; $t_7 = 2.40$,

CellPress
OPEN ACCESS

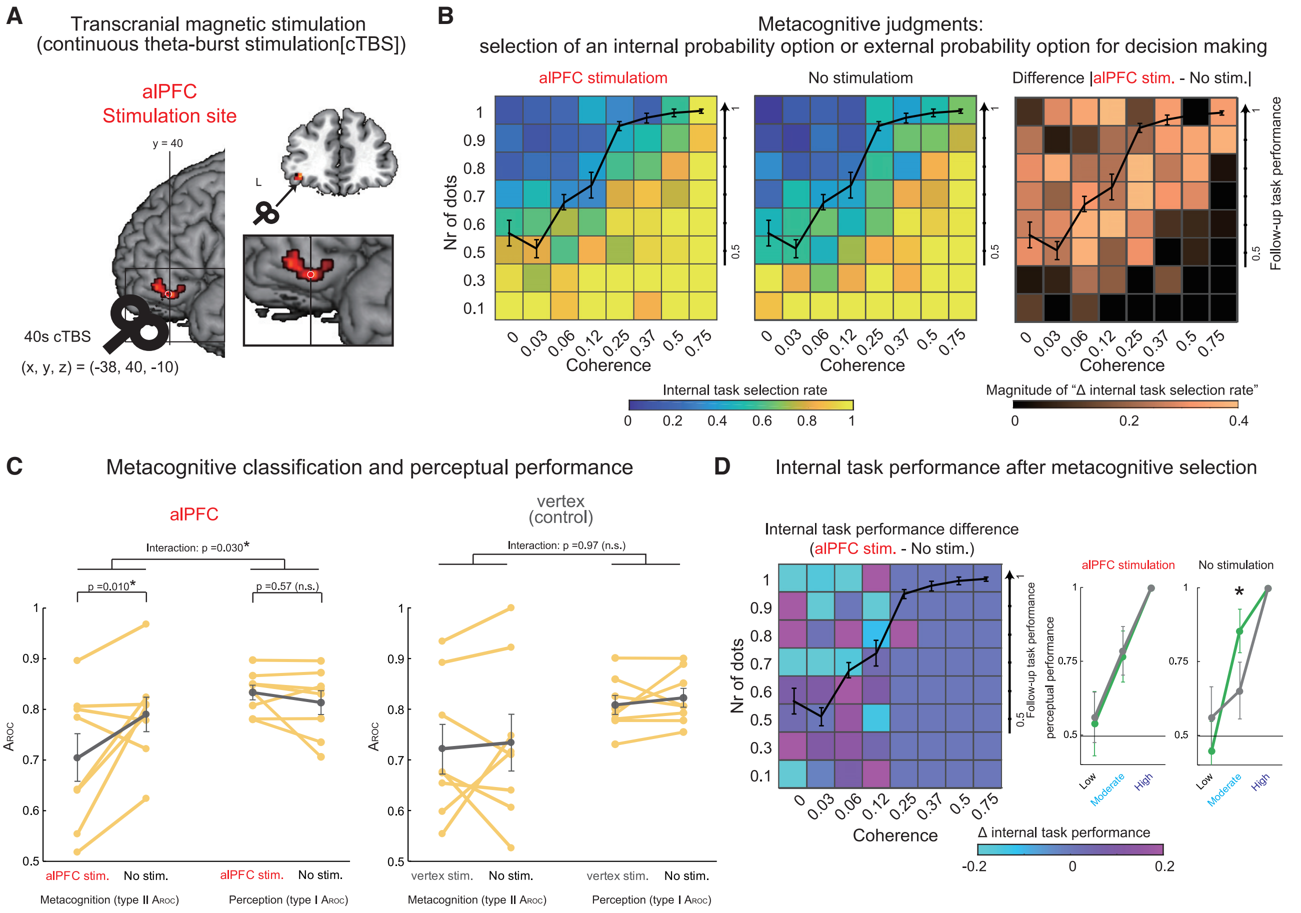

Figure 7. Modulating activity in the alPFC with TMS (cTBS) caused impairments in metacognitive judgment

(A) TMS (cTBS) was applied to the left $alPFC_{47}$ (red dot; see also Figure 5A).

(B) Preference for choosing the internal probability task in the metacognitive judgment stage after $alPFC_{47}$ stimulation (left) and no stimulation (center). The magnitude of differences between $alPFC_{47}$ stimulation and no stimulation (right) shows that differences were maximal around the black line indicating the performance level on the follow-up task (internal probability trials without any prior metacognitive judgment phase).

(C) Comparisons of metacognitive performance (type II and type I A_{ROC}) between stimulation and no stimulation. $alPFC_{47}$ stimulation significantly impaired type II A_{ROC} compared with no stimulation, whereas $alPFC_{47}$ stimulation did not impair type I A_{ROC} (left). Neither type II A_{ROC} or type I A_{ROC} were impaired when the vertex (control site) was stimulated (right). The gray line indicates the mean across participants.

(D) Left: performance change with $alPFC_{47}$ TMS compared with no stimulation. The light blue squares indicate trials that were performed worse after $alPFC_{47}$ TMS. These tended to occur at moderate coherence levels (0.06, 0.12) in challenge trials as opposed to inevitable trials (see green and gray squares, respectively, in Figure 3D). Right: perceptual performance during challenge trials (green line) was higher than during inevitable trials (gray line) for the no-TMS condition. However, this pattern no longer held when TMS was applied to the $alPFC_{47}$.

N = 8; error bars indicate SEM across participants; *$p < 0.05$, paired t test.

p = 0.046, paired t test). However, the normal benefit conferred by the opportunity to make prospective metacognitive judgments was reversed by $alPFC_{47}$ stimulation ($alPFC_{47}$ versus no stimulation for moderate coherence, $t_7 = 2.62$, $p = 0.034$; especially for coherence 0.06, $t_7 = 3.69$, $p = 0.0076$, paired t test) (Figure S8D). These observations suggest that the $alPFC_{47}$ plays an essential role in proactively utilizing prospective metacognition to optimize subsequent decision-making and obtaining rewards.

DISCUSSION

Two types of choice-outcome contingencies must be taken into account when making a decision (Figure 8). The first is the contingency between the choice and the outcome: how likely is the choice to lead to the outcome? The second contingency, however, concerns how likely it is that the choice will be made correctly by the agent making the decision. We know that learning contingencies between choices and outcomes depends on the cortex in and lateral to the lateral orbitofrontal sulcus in humans and macaques (Chau et al., 2015; Jocham et al., 2016; Neubert et al., 2015; Noonan et al., 2010, 2017; Rudebeck et al., 2017; Walton et al., 2010) and that such contingencies are represented in the medial frontal cortex during decision-making (Figure 4A). Here we show that the second type of contingency depends on the adjacent but more dorsal alPFC.

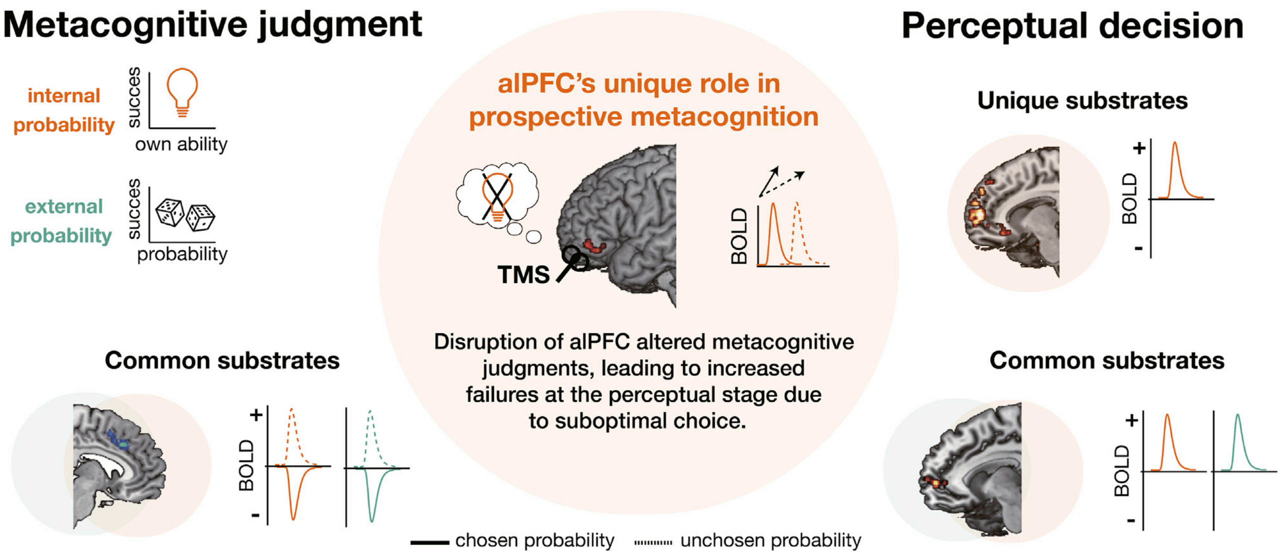

Figure 8. Summary: the alPFC accumulates evidence of internal probability for optimal prospective metacognitive judgments
A prospective metacognitive decision requires consideration of two kinds of probabilities: the internal probability—the likelihood of success given one's ability to overcome the difficulty of the task—and the external probability—the fact that the stochastic nature of the environment means that reward is not always delivered even when the choice has been performed correctly. We found common and unique substrates associated with internal probability (orange color) and external probability (green color) when making metacognitive judgments and when making perceptual decisions about the selected task. One area showed a unique profile contributing to prospective metacognition: the alPFC carried information about the internal probability associated with the chosen (solid bold line) and unchosen (dotted bold line) task in a similar way (positive sign), but evidence accumulation for the option that was chosen occurred more quickly (see neural activity during metacognitive judgment and perceptual decision: hypotheses in the results). We also showed the alPFC's casual role in prospective metacognition by applying TMS to the alPFC; this caused changes in the pattern of metacognitive judgments that, in turn, led to increased failures at the perceptual decision-making stage, particularly in moderate coherence level trials, where performance was improved by the opportunity to make a prior metacognitive judgment to tackle the trial. The alPFC activity profile (center) differed from that seen in other frontal areas that were involved during metacognitive and perceptual decisions: dACC (left; activity correlated with the internal and external probabilities associated with the chosen and unchosen options during the metacognitive judgment stage), vmPFC (right; activity correlated with the internal and external probabilities associated with the chosen options during the perceptual decision-making stage; first neural processing hypothesis), and FPm (particularly related to some aspect of internal probability evaluation during the perceptual decision-making stage; second neural processing hypothesis).

Internal probability must be evaluated prospectively; the decision maker must estimate their ability to make the choice prior to taking it. Activity in the alPFC predominantly reflected internal probability evidence, and, unlike in all other areas, it arose regardless of whether the internal probability option was taken or rejected. However, the speed with which activity related to internal probability ramped up during the metacognitive decision phase was faster when the internal probability option was ultimately chosen. By analogy with the activity patterns seen in the frontal and parietal cortex, it seems likely that alPFC activity reflects an evidence accumulation process but that it is specifically an accumulator of internal probability, guiding comparisons of internally accessed probability estimates and reward contingencies afforded by the environment. Although its activity does not increase as evidence for choosing the external task increases, its activity is reduced by external probability (Figure S6C). This may make it possible to prospectively evaluate internal probability against external probability during metacognitive judgments and to identify the best internal probability tasks to perform even when there might be almost as much evidence for tasking the external option (Figures 3D and 7D). Identifying internal probability tasks to perform in this way (in challenge trials) depends on metacognitive judgment rather than just attentional modulation (Figure S6).

The contribution of the alPFC is clearly distinct from that of other prefrontal areas, including the FPm, that carry information about internal probability, but only when participants are actually making perceptual decisions as opposed to the prior metacognitive judgments. Manipulation of alPFC activity with continuous TBS led to changes in the way metacognitive judgments were made, which, in turn, led to participants choosing to tackle perceptual decisions they failed. Few studies have assessed the causal importance of prefrontal cortical regions for metacognitive judgment. Fleming et al. (2014) demonstrated that anterior prefrontal lesions result in deficits of retrospective metacognitive assessments for perceptual decisions even in individuals with an intact bilateral alPFC. This study, together with a pioneering MRI-based study from the same group (Fleming et al., 2010), suggests that the frontopolar cortex (area 10) is essential for retrospective metacognition. Ryals et al. (2016) also demonstrated that cTBS targeted to the frontopolar area 10 (x = ±29, y = +66, z = +10 mm) changed judgments of learning and retrospective confidence judgments but cTBS targeted to the dorsolateral prefrontal area 46 (x = ±52, y = +15, z = +9 mm) did not. These observations are consistent with our finding that FPm (area 10) activity is associated with confidence in the perceptual decision task (Figures 4B, 4C, and 6). Macaque studies found that the more dorsal prefrontal areas 10, 9, and 6 are essential

CellPress
OPEN ACCESS

for retrospective confidence judgments on mnemonic decision performance (Miyamoto et al., 2017, 2018).

The present study suggests a specific role of the alPFC in prospective, metacognitive evaluation of how likely an agent is able to make a choice correctly. It has been noted previously that this region is especially active when decision-making occurs under ambiguity (Levy et al., 2010) and when it is necessary to estimate which model or approach to a decision-making problem is best to adopt (Charpentier et al., 2020; Lee et al., 2014). It is also interesting to note that it is difficult to identify a brain region with the same connectional anatomy in macaques (Neubert et al., 2015).

STAR★METHODS

Detailed methods are provided in the online version of this paper and include the following:

- KEY RESOURCES TABLE
- RESOURCE AVAILABILITY
  - Lead contact
  - Data and code availability
  - Materials availability
- EXPERIMENTAL MODEL AND SUBJECT DETAILS
  - Participants
  - Experimental Procedure
- METHOD DETAILS
  - Behavioral tasks
  - FMRI data acquisition and data processing
  - Transcranial magnetic stimulation (TMS)
- QUANTIFICATION AND STATISTICAL ANALYSIS
  - Behavioral data
  - Functional MRI data

SUPPLEMENTAL INFORMATION

Supplemental Information can be found online at https://doi.org/10.1016/j.neuron.2021.02.024.

ACKNOWLEDGMENTS

K.M. was funded by a JSPS fellowship for research abroad. N.T. was funded by a DTC ESRC studentship (ES/J500112/1). M.F.S.R. and M.K.W. were funded by a Wellcome senior investigator award (WT100973AIA). We would like to thank all members of the Rushworth lab for great discussions regarding this project.

AUTHOR CONTRIBUTIONS

K.M. and M.F.S.R. designed the research. K.M., N.T., K.K., and M.L. conducted experiments and collected data. K.M., N.T., M.K.W., and M.F.S.R. conceived behavioral and neural analyses. K.M. and N.T. conducted data analyses. N.T., A.L., and L.V. developed the setup and supervised the TMS experiments. K.M., N.T., M.K.W., and M.F.S.R. wrote the manuscript, and all authors provided expertise and feedback.

DECLARATION OF INTERESTS

The authors declare no competing interests.

INCLUSION AND DIVERSITY

We worked to ensure gender balance in the recruitment of human subjects. We worked to ensure ethnic or other types of diversity in the recruitment of human subjects. We worked to ensure that the study questionnaires were prepared in an inclusive way. While citing references scientifically relevant for this work, we also actively worked to promote gender balance in our reference list. The author list of this paper includes contributors from the location where the research was conducted who participated in the data collection, design, analysis, and/or interpretation of the work.

Received: July 30, 2020
Revised: January 13, 2021
Accepted: February 19, 2021
Published: March 16, 2021

REFERENCES

Bennur, S., and Gold, J.I. (2011). Distinct representations of a perceptual decision and the associated oculomotor plan in the monkey lateral intraparietal area. J. Neurosci. *31*, 913–921.

Boorman, E.D., Behrens, T.E., Woolrich, M.W., and Rushworth, M.F. (2009). How green is the grass on the other side? Frontopolar cortex and the evidence in favor of alternative courses of action. Neuron *62*, 733–743.

Boorman, E.D., Behrens, T.E., and Rushworth, M.F. (2011). Counterfactual choice and learning in a neural network centered on human lateral frontopolar cortex. PLoS Biol. *9*, e1001093.

Boorman, E.D., Rushworth, M.F., and Behrens, T.E. (2013). Ventromedial prefrontal and anterior cingulate cortex adopt choice and default reference frames during sequential multi-alternative choice. J. Neurosci. *33*, 2242–2253.

Charpentier, C.J., Iigaya, K., and O'Doherty, J.P. (2020). A Neuro-computational Account of Arbitration between Choice Imitation and Goal Emulation during Human Observational Learning. Neuron *106*, 687–699.e7.

Chau, B.K., Sallet, J., Papageorgiou, G.K., Noonan, M.P., Bell, A.H., Walton, M.E., and Rushworth, M.F. (2015). Contrasting Roles for Orbitofrontal Cortex and Amygdala in Credit Assignment and Learning in Macaques. Neuron *87*, 1106–1118.

De Martino, B., Fleming, S.M., Garrett, N., and Dolan, R.J. (2013). Confidence in value-based choice. Nat. Neurosci. *16*, 105–110.

Fleming, S.M., Weil, R.S., Nagy, Z., Dolan, R.J., and Rees, G. (2010). Relating introspective accuracy to individual differences in brain structure. Science *329*, 1541–1543.

Fleming, S.M., Huijgen, J., and Dolan, R.J. (2012). Prefrontal contributions to metacognition in perceptual decision making. J. Neurosci. *32*, 6117–6125.

Fleming, S.M., Ryu, J., Golfinos, J.G., and Blackmon, K.E. (2014). Domain-specific impairment in metacognitive accuracy following anterior prefrontal lesions. Brain *137*, 2811–2822.

Fouragnan, E.F., Chau, B.K.H., Folloni, D., Kolling, N., Verhagen, L., Klein-Flügge, M., Tankelevitch, L., Papageorgiou, G.K., Aubry, J.F., Sallet, J., and Rushworth, M.F.S. (2019). The macaque anterior cingulate cortex translates counterfactual choice value into actual behavioral change. Nat. Neurosci. *22*, 797–808.

Gold, J.I., and Shadlen, M.N. (2007). The neural basis of decision making. Annu. Rev. Neurosci. *30*, 535–574.

Hanks, T.D., and Summerfield, C. (2017). Perceptual Decision Making in Rodents, Monkeys, and Humans. Neuron *93*, 15–31.

Hunt, L.T., Kolling, N., Soltani, A., Woolrich, M.W., Rushworth, M.F., and Behrens, T.E. (2012). Mechanisms underlying cortical activity during value-guided choice. Nat. Neurosci. *15*, 470–476, S1–S3.

Jocham, G., Brodersen, K.H., Constantinescu, A.O., Kahn, M.C., Ianni, A.M., Walton, M.E., Rushworth, M.F., and Behrens, T.E. (2016). Reward-Guided Learning with and without Causal Attribution. Neuron *90*, 177–190.

Johnen, V.M., Neubert, F.X., Buch, E.R., Verhagen, L., O'Reilly, J.X., Mars, R.B., and Rushworth, M.F. (2015). Causal manipulation of functional connectivity in a specific neural pathway during behaviour and at rest. eLife *4*, e04585.

Kahneman, D., and Tversky, A. (1979). Prospect Theory: An Analysis of Decision under Risk. Econometrica *47*, 263–291.

Kiani, R., and Shadlen, M.N. (2009). Representation of confidence associated with a decision by neurons in the parietal cortex. Science *324*, 759–764.

Kim, J.-N., and Shadlen, M.N. (1999). Neural correlates of a decision in the dorsolateral prefrontal cortex of the macaque. Nat. Neurosci. *2*, 176–185.

Kolling, N., Behrens, T.E., Mars, R.B., and Rushworth, M.F. (2012). Neural mechanisms of foraging. Science *336*, 95–98.

Kolling, N., Wittmann, M.K., Behrens, T.E., Boorman, E.D., Mars, R.B., and Rushworth, M.F. (2016). Value, search, persistence and model updating in anterior cingulate cortex. Nat. Neurosci. *19*, 1280–1285.

Kolling, N., Scholl, J., Chekroud, A., Trier, H.A., and Rushworth, M.F.S. (2018). Prospection, Perseverance, and Insight in Sequential Behavior. Neuron *99*, 1069–1082.e7.

Lee, S.W., Shimojo, S., and O'Doherty, J.P. (2014). Neural computations underlying arbitration between model-based and model-free learning. Neuron *81*, 687–699.

Levy, I., Snell, J., Nelson, A.J., Rustichini, A., and Glimcher, P.W. (2010). Neural representation of subjective value under risk and ambiguity. J. Neurophysiol. *103*, 1036–1047.

Mackey, S., and Petrides, M. (2010). Quantitative demonstration of comparable architectonic areas within the ventromedial and lateral orbital frontal cortex in the human and the macaque monkey brains. Eur. J. Neurosci. *32*, 1940–1950.

Maniscalco, B., and Lau, H. (2012). A signal detection theoretic approach for estimating metacognitive sensitivity from confidence ratings. Conscious. Cogn. *21*, 422–430.

Meder, D., Kolling, N., Verhagen, L., Wittmann, M.K., Scholl, J., Madsen, K.H., Hulme, O.J., Behrens, T.E.J., and Rushworth, M.F.S. (2017). Simultaneous representation of a spectrum of dynamically changing value estimates during decision making. Nat. Commun. *8*, 1942.

Miyamoto, K., Osada, T., Setsuie, R., Takeda, M., Tamura, K., Adachi, Y., and Miyashita, Y. (2017). Causal neural network of metamemory for retrospection in primates. Science *355*, 188–193.

Miyamoto, K., Setsuie, R., Osada, T., and Miyashita, Y. (2018). Reversible Silencing of the Frontopolar Cortex Selectively Impairs Metacognitive Judgment on Non-experience in Primates. Neuron *97*, 980–989.e6.

Murray, E.A., and Rudebeck, P.H. (2018). Specializations for reward-guided decision-making in the primate ventral prefrontal cortex. Nat. Rev. Neurosci. *19*, 404–417.

Neubert, F.X., Mars, R.B., Thomas, A.G., Sallet, J., and Rushworth, M.F. (2014). Comparison of human ventral frontal cortex areas for cognitive control and language with areas in monkey frontal cortex. Neuron *81*, 700–713.

Neubert, F.X., Mars, R.B., Sallet, J., and Rushworth, M.F. (2015). Connectivity reveals relationship of brain areas for reward-guided learning and decision making in human and monkey frontal cortex. Proc. Natl. Acad. Sci. USA *112*, E2695–E2704.

Nichols, T., Brett, M., Andersson, J., Wager, T., and Poline, J.B. (2005). Valid conjunction inference with the minimum statistic. Neuroimage *25*, 653–660.

Noonan, M.P., Walton, M.E., Behrens, T.E., Sallet, J., Buckley, M.J., and Rushworth, M.F. (2010). Separate value comparison and learning mechanisms in macaque medial and lateral orbitofrontal cortex. Proc. Natl. Acad. Sci. USA *107*, 20547–20552.

Noonan, M.P., Chau, B.K.H., Rushworth, M.F.S., and Fellows, L.K. (2017). Contrasting Effects of Medial and Lateral Orbitofrontal Cortex Lesions on Credit Assignment and Decision-Making in Humans. J. Neurosci. *37*, 7023–7035.

Papageorgiou, G.K., Sallet, J., Wittmann, M.K., Chau, B.K.H., Schüffelgen, U., Buckley, M.J., and Rushworth, M.F.S. (2017). Inverted activity patterns in ventromedial prefrontal cortex during value-guided decision-making in a less-is-more task. Nat. Commun. *8*, 1886.

Petrides, M., and Pandya, D.N. (1999). Dorsolateral prefrontal cortex: comparative cytoarchitectonic analysis in the human and the macaque brain and corticocortical connection patterns. Eur. J. Neurosci. *11*, 1011–1036.

Petrides, M., and Pandya, D.N. (2002). Comparative cytoarchitectonic analysis of the human and the macaque ventrolateral prefrontal cortex and corticocortical connection patterns in the monkey. Eur. J. Neurosci. *16*, 291–310.

Platt, M.L., and Huettel, S.A. (2008). Risky business: the neuroeconomics of decision making under uncertainty. Nat. Neurosci. *11*, 398–403.

Preuschoff, K., Bossaerts, P., and Quartz, S.R. (2006). Neural differentiation of expected reward and risk in human subcortical structures. Neuron *51*, 381–390.

Rossi, S., Hallett, M., Rossini, P.M., and Pascual-Leone, A.; Safety of TMS Consensus Group (2009). Safety, ethical considerations, and application guidelines for the use of transcranial magnetic stimulation in clinical practice and research. Clin. Neurophysiol. *120*, 2008–2039.

Rossini, P.M., Burke, D., Chen, R., Cohen, L.G., Daskalakis, Z., Di Iorio, R., Di Lazzaro, V., Ferreri, F., Fitzgerald, P.B., George, M.S., et al. (2015). Non-invasive electrical and magnetic stimulation of the brain, spinal cord, roots and peripheral nerves: Basic principles and procedures for routine clinical and research application. An updated report from an I.F.C.N. Committee. Clin. Neurophysiol. *126*, 1071–1107.

Rudebeck, P.H., Saunders, R.C., Lundgren, D.A., and Murray, E.A. (2017). Specialized Representations of Value in the Orbital and Ventrolateral Prefrontal Cortex: Desirability versus Availability of Outcomes. Neuron *95*, 1208–1220.e5.

Rushworth, M.F., and Behrens, T.E. (2008). Choice, uncertainty and value in prefrontal and cingulate cortex. Nat. Neurosci. *11*, 389–397.

Ryals, A.J., Rogers, L.M., Gross, E.Z., Polnaszek, K.L., and Voss, J.L. (2016). Associative Recognition Memory Awareness Improved by Theta-Burst Stimulation of Frontopolar Cortex. Cereb. Cortex *26*, 1200–1210.

Smith, S.M., Jenkinson, M., Woolrich, M.W., Beckmann, C.F., Behrens, T.E., Johansen-Berg, H., Bannister, P.R., De Luca, M., Drobnjak, I., Flitney, D.E., et al. (2004). Advances in functional and structural MR image analysis and implementation as FSL. Neuroimage *23* (*Suppl 1*), S208–S219.

Tobler, P.N., Christopoulos, G.I., O'Doherty, J.P., Dolan, R.J., and Schultz, W. (2009). Risk-dependent reward value signal in human prefrontal cortex. Proc. Natl. Acad. Sci. USA *106*, 7185–7190.

Trudel, N., Scholl, J., Klein-Flugge, M.C., Fouragnan, E., Tankelevitch, L., Wittmann, M.K., and Rushworth, M.F.S. (2021). Polarity of uncertainty representation during exploration and exploitation in ventromedial prefrontal cortex. Nat. Hum. Behav. *5*, 83–98.

Walton, M.E., Behrens, T.E., Buckley, M.J., Rudebeck, P.H., and Rushworth, M.F. (2010). Separable learning systems in the macaque brain and the role of orbitofrontal cortex in contingent learning. Neuron *65*, 927–939.

Wittmann, M.K., Kolling, N., Akaishi, R., Chau, B.K., Brown, J.W., Nelissen, N., and Rushworth, M.F. (2016a). Predictive decision making driven by multiple time-linked reward representations in the anterior cingulate cortex. Nat. Commun. *7*, 12327.

Wittmann, M.K., Kolling, N., Faber, N.S., Scholl, J., Nelissen, N., and Rushworth, M.F. (2016b). Self-Other Mergence in the Frontal Cortex during Cooperation and Competition. Neuron *91*, 482–493.

Wunderlich, K., Dayan, P., and Dolan, R.J. (2012). Mapping value based planning and extensively trained choice in the human brain. Nat. Neurosci. *15*, 786–791.

CellPress
OPEN ACCESS

STAR★METHODS

KEY RESOURCES TABLE

REAGENT or RESOURCE	SOURCE	IDENTIFIER
Software and algorithms		
Presentation	Neurobehavioral systems	RRID: SCR_002521
MATLAB R2019a	MathWorks	RRID: SCR_001622
FSL	FMRIB, Oxford	RRID: SCR_002823
Brainsight	Rogue Research	RRID: SCR_009539
Spike2 Software	Cambridge Electronic Design Limited	RRID: SCR_000903
Others		
Magstim Rapid2 stimulator (TMS)	Magstim	https://www.magstim.com
D440 Isolated EMG amplifier	Digitimer	https://www.digitimer.com/
Hum Bug 50/60 Hz Noise Eliminator	Quest Scientific	https://www.digitimer.com/
CED power1401	Cambridge Electronic Design Limited	RRID: SCR_017282

RESOURCE AVAILABILITY

Lead contact

Further information and requests for resources and reagents should be directed to and will be fulfilled by the Lead Contact, Kentaro Miyamoto (kentaro.miyamoto@psy.ox.ac.uk).

Data and code availability

The data and code that support the findings of this study will be shared via Oxford University Research Archive (https://ora.ox.ac.uk/). They are also available from the corresponding author upon reasonable request.

Materials availability

Materials are available from the corresponding author upon reasonable request..

EXPERIMENTAL MODEL AND SUBJECT DETAILS

Participants

Twenty-six participants took part in the functional MRI experiment (Experiment 1). Participants were excluded because they exhibited excessive motion during the scan (N = 2) or because of premature termination of an experimental session (N = 1) (final sample: 23 participants; 15 female; age (mean ± SD), 28.2 ± 6.7). Ten participants took part in the TMS experiment (Experiment 2). Two participants were excluded because of premature termination of an experimental session (final sample: 8 participants; 5 female; age (mean ± SD), 25.8 ± 4.4). Because of COVID-19, UK national and university-mandated COVID-19-related social distancing requirements, meant that we had to stop further data collection. The study was approved by the Central Research Ethics Committee (Experiment 1: MSD-IDREC-R51506/RE002, Experiment 2: R65502/RE001) at the University of Oxford. All participants gave informed consent.

Experimental Procedure

We conducted two experiments. The first experiment assessed the neural correlates of metacognitive and perceptual decisions with fMRI (Experiment 1), while the second experiment probed the causal contribution of alPFC in prospective metacognition using transcranial magnetic stimulation (TMS) (Experiment 2). Experiments used a different sample of participants.

Experiment 1

Participants took part in one behavioral task session (Session 1) and two magnetic resonance imaging (MRI) sessions (Sessions 2 and 3) on separate days. Each session lasted approximately 1.5 hours, including one hour of scanning for Session 2 and 3. Participants received £10 per hour and a bonus based on task performance (accumulated across sessions: £5–£7 per session). In the behavioral session (Session 1), participants first practiced the internal and external probability tasks in an alternate order, and thereby learned the association between task (external versus internal) and reward probabilities (35 min). Next, they learned how to perform the main metacognition task (20 min). In Sessions 2 and 3, participants were first reminded about the task and performed twenty practice trials

outside the scanner. Each fMRI scanning session included 195 trials and lasted for 45–50 min. To acquire reliable and robust data we repeated two sessions with a different stimulus set. After each scanning session, participants performed 195 trials of a follow-up task that lasted for 10 min outside the scanner (see section 'Behavioral tasks' for details). The follow-up task did not contain a metacognitive decision stage and only internal probability trials were presented. The task utilized the identical RDK stimuli used for the internal probability trials in the fMRI session and hence also comprised 195 trials. The inclusion of the follow-up task made it possible to assess, on average, how well participants performed internal probability tasks of different coherence levels. This made it possible to understand how each coherence level was linked to a particular probability of being performed correctly by each participant.

Experiment 2

The second experiment included four sessions: a behavioral task session (Session 1; 2.5 hours), a structural MRI session (Session 2; 30 minutes) and two continuous theta burst (cTBS) transcranial magnetic stimulation sessions (Sessions 3 and 4; each 2 hours). The first session was similar to the behavioral session in Experiment 1 during which participants were instructed about the task and learnt the difference between internal and external probability tasks. Additionally, in Session 1 we assessed participants' motor thresholds which determined the intensity of cTBS stimulation that was used in later cTBS sessions (see section 'Transcranial magnetic stimulation (TMS)' for more details). To predict participants' tolerance and comfort with the stimulation protocol in Sessions 3 and 4, we first applied a milder stimulation protocol, 'a taster session' during Session 1. The taster session included a stimulation protocol of a 10 s train of cTBS with the stimulator output set to 20%. Session 2 served to acquire structural MRI scans that would guide the neuronavigated localization of the TMS target areas in the subsequent two sessions. Session 3 and 4 consisted each of two blocks: a stimulation block and a no-stimulation block. For each block, participants performed a shortened version of the experimental task used in Experiment 1 (Session 2 and 3). Each block lasted for 30 min (225 trials). However, because stimulation effects decrease rapidly across a period of 30 minutes, we focused on the initial 140 trials, which lasted for a duration of 20 min. that is 2/3 of the trials, after the termination of 40 s train of cTBS. Stimulation was applied before one block ("TMS block"), but not the other ("control block") within each session 3 and 4. The stimulation order within session was counterbalanced across participants. The difference between Session 3 and 4 was their stimulation site: the stimulation site was either centered on aIPFC [MNI x/y/z- coordinate: −38, 40, −10] or vertex [MNI x/y/z- coordinate: 0, −34, 72], with cTBS being applied immediately before the start of the "TMS block." Further counterbalancing meant that some participants performed aIPFC sessions first and some performed vertex sessions first. As a result of the various types of counterbalancing, participants performed sessions in the following orders (2 participants: Session 3, aIPFC; Session 4, vertex; TMS block before control block. 2 participants: Session 3, vertex; Session 4, aIPFC; TMS block before control block. 2 participants: Session 3, aIPFC; Session 4, vertex; TMS block after control block. 2 participants: Session 3, vertex; Session 4, aIPFC; TMS block after control block). As in Experiment 1, participants were asked to perform the follow-up task including only internal probability options. The follow-up task included 225 follow-up task trials and lasted for 12 min. To make sure that these trials were not affected by cTBS stimulation the follow-up task was performed after the "control block." The participants took at least a 30 min break from the end of the "TMS block" to the start of the "control block" to decrease the possibility of any remaining effects of TMS.

METHOD DETAILS

Behavioral tasks

Experiment 1 and 2 used the same behavioral task. The main metacognition task comprised two stages: each trial comprised a metacognitive judgment followed by a perceptual decision and a final outcome phase (Figure 1B). In the metacognitive judgment stage, participants had to choose one of the two RDK stimuli that were presented simultaneously. One RDK represented an external probability task, the other represented an internal probability task. Either stimulus could appear with the same frequency on the left or on the right of the screen. The internal probability task contained a full number of dots (number of dots = 100; external probability of reward indicated by the number of dots was always 1), but the movements of the dots were ambiguous (0%, 3%, 6%, 12%, 25%, 37%, 50%, or 75% denote the different coherence levels). The other stimulus represented the external probability task, containing a smaller number of dots (10, 30, 50, 60, 70, 80, 90, or 100 indicating external probabilities of between 0.1–1.0 of reward) but all dots moved in the same direction (100% coherence) which was always easily discernible by every participant. However, note that the internal probability task always comprised the full number of dots and the external probability task always utilized 100% coherence – meaning that always only one of the two dimensions varied per task type while the other one was fixed. All the combination of 8 internal probability tasks and 8 external probability tasks were offered during the metacognitive judgment stage. In the Metacognition stage, each RDK stimulus was moving upward or downward for 1.5 s. After disappearance of the stimuli, participants chose the task they want to perform in the subsequent perceptual decision stage by pressing a button with their right hand. After a stimulus onset asynchrony (SOA) (Experiment 1, 2.5–8.5 s [Poisson distribution, mean of 3 s]; Experiment 2, 1 s; note that we did not have to control for the BOLD response in the second experiment and therefore SOAs are shorter, moreover given the limited duration of cTBS effects it was important to collect trials more quickly in Experiment 2), participants moved into a perceptual decision stage where the same stimulus that they chose in the metacognitive judgment stage appeared again for 1.5 s. This time however, the direction of dot motion was rotated by ± 90 degree. For example, if they selected the external probability option in the first stage and the stimulus was moving upward they could not know until the second stage perceptual decision whether the stimulus would be moving leftward or rightward. After disappearance of stimuli, participants were asked to answer if the RDK stimulus is moving leftward or rightward by pressing a button. The rotation of the stimulus was introduced to prevent participants from making a perceptual decision about motion direction

CellPress
OPEN ACCESS

during the metacognitive judgment phase of the trial instead of during the subsequent perceptual decision phase of the trial. However, we wanted the participants to estimate and compare the utility of choosing either the internal or external probability options to make an optimal metacognitive judgment. In the experiment, we rotated the direction of the stimulus chosen in the metacognitive judgment phase of the trial either clockwise or anticlockwise randomly when it appeared at the perceptual decision phase of every trial. Therefore, participants could not predict the motion direction in the perceptual decision stage from that in the metacognitive judgment stage (Bennur and Gold, 2011). After an SOA (Experiment 1, 2.5–8.5 s; Experiment 2, 1 s), outcome feedback appeared for 1 s. If participants judged the motion direction correctly, a reward ('tick' symbol on the center of screen indicated success) was given according to the external probability indicated by the chosen RDK stimulus while, otherwise, no reward ('X' symbol on the center of screen indicated failure) was given. When they misjudged the motion direction, no reward was given irrespective of the probability. A yellow bar which indicates the total number of 'correct' outcomes also appeared on the bottom of screen during the feedback period. Based on the number of 'correct' outcomes, participants received a monetary bonus reward after the experiment. After 1 s of inter-trial interval (ITI), the next trial started. As a further counterbalancing procedure, in Experiment 1, for approximately half of the participant sample (n = 10), the configuration of stimuli locations during the metacognitive task was different; in the metacognitive judgment stage, two RDK stimuli appeared at the top and bottom of the screen and they moved either rightward or leftward; in the perceptual decision stage, the chosen RDK stimulus remained on the same location but the direction of dot motion was rotated by ± 90 degree; thus, they answered whether the RDK stimulus was moving upward or downward. We confirmed that behavioral performance was comparable between the two subgroups of participants: LR subgroup (n = 13; RDK moved leftward or rightward during perceptual decisions) and UD subgroup (n = 10; RDK moved upward or downward during perceptual decisions) (type II A_{ROC}: LR, 0.72 ± 0.033 [mean ± SEM]; UD, 0.64 ± 0.044; LR versus UD, t_{21} = 1.47, p = 0.15. type I A_{ROC}: LR, 0.84 ± 0.015; UD, 0.82 ± 0.018; LR versus UD, t_{21} = 0.95, p = 0.34). During the task, participants were asked to fixate on the center of the screen. Eye positions were monitored in Experiment 1 with an eye tracker (Eyelink 1000, SR Research). We used eye tracking data to confirm that all participants engaged in performing the task during fMRI scanning. The data were not analyzed in the presented study.

The follow-up task comprised only the perceptual decision stage (Figure 1B). We used the same set of RDK stimuli for the internal probability task as in the previous metacognition task and hence the number of trials is identical for both tasks. RDK were moving upward or downward (for UD subgroup in Experiment 1 [n = 10], they were moving rightward or leftward). Participants were asked to judge the motion direction by pressing a button.

FMRI data acquisition and data processing

Imaging data in Experiment 1 were acquired with a Siemens Prisma 3T MRI using a multiband T2*-weighted echo planar imaging sequence with acceleration factor of two and a 32-channel head-coil. Slices were acquired with an oblique angle of 30 deg to the PC-AC line to reduce signal dropout in frontal pole. Other acquisition parameters included 2.4 × 2.4 × 2.4 mm voxel size, TE = 30 ms, TR = 1230 ms, 60° flip angle, a 240 mm field of view and 60 slices per volume. For each session, a fieldmap (2.4 × 2.4 × 2.4mm) was acquired to reduce spatial distortions. Bias correction was applied directly to the scan. A structural scan was obtained with slice thickness = 1 mm; TR = 1900 ms, TE = 3.97 ms and 1 × 1 × 1 mm voxel size. Imaging data were analyzed using FMRIB's Software Library (FSL) (Smith et al., 2004). Preprocessing stages included motion correction, correction for spatial distortion by applying the fieldmap, brain extraction, high-pass filtering and spatial smoothing using full-width half maximum of 5 mm. Images were co-registered to an individuals' high-resolution structural image and then nonlinearly registered to the MNI template using 12 degrees of freedom. In Experiment 2, we obtained a structural scan using the same protocol with a lager field of view covering the nose tip and both ears, which serve as the landmarks for frameless stereotactic neuronavigation (see the next 'Transcranial magnetic stimulation (TMS)' section).

Transcranial magnetic stimulation (TMS)

TMS was applied using a Magstim Rapid stimulator which was connected to a 50 mm figure-8 coil (Johnen et al., 2015). In Session 1 of Experiment 2, we assessed participants active motor threshold (AMT) for the left M1 'hotspot', which is the scalp location where TMS evoked the largest MEP amplitude in right first dorsal interosseous (FDI) (Rossini et al., 2015) (mean ± SD: 39.5% ± 5.3% stimulator output). Electromyographic (EMG) activity in right FDI was recorded with bipolar surface Ag-AgCl electrode montages. Responses were bandpass filtered between 10 and 1000 Hz, with additional 50 Hz notch filtering, sampled at 5000 Hz, and recorded using a D440 Isolated EMG amplifier (Digitimer), a Hum Bug 50/60 Hz Noise Eliminator (Quest Scientific), a CEDmicro1401 Mk.II A/D converter, and PC running Spike2 (Cambridge Electronic Design).

The region of interest was left alPFC (Session 3 or 4) with MNI x/y/z-peak coordinates (−38, 40, −10), which was identified by the previous fMRI experiment (Experiment 1; see Figure 5A). We used the same coordinate for left alPFC stimulation. To stimulate vertex, the coil was placed over MNI x/y/z-peak coordinates (0, −34, 72). No neural activity with any relation to either internal or external probability was found at this vertex location suggesting that it was an appropriate control site. The location was projected onto the high-resolution, T1-weighted MRI brain scan of each participant using frameless stereotactic neuronavigation (Brainsight; Rogue Research). We used a standard continuous theta-burst stimulation (cTBS) protocol to stimulate alPFC and vertex: 600 pulses were administered in bursts of three pulses at 5 Hz (total stimulation duration was 40 s). TMS coils were held in place tangentially to the skull

by an experimenter during stimulation. For each participant, stimulation intensity was determined by 80% of the AMT (Rossi et al., 2009). The use of such a low subthreshold intensity (80% AMT) had the advantage of ensured decreased spread of stimulation away from the targeted site and enabled us to focus on the alPFC site.

QUANTIFICATION AND STATISTICAL ANALYSIS

Behavioral data

To evaluate performance during the metacognitive judgment stage (Figures 3B, 6, and 7C), we employed an analysis based on signal detection theory (Maniscalco and Lau, 2012). Specifically, we classified the metacognitive judgment trials with coherences of 0.03, 0.06, 0.12, and 0.25 into those trials in which it was optimal for participants to choose the internal task and into those trials in which it would be optimal to choose the external task. For each participant, if the external probability of reward offered by the external task option was higher than the probability of reward that would be expected given the baseline level of perceptual performance of the internal task option (obtained during the follow-up task), then such trials were categorized as external task optimal trials. If not, they were categorized as the internal task optimal trials. Based on the proportion of trials in which they chose the internal task option when the internal option was optimal (Hit trials) and when the external option was optimal (False alarm [FA] trials), we calculated the area under the ROC curve (type II A_{ROC}). We first plotted points indicating the proportion of 'Hit' trials (Hit rate; y axis) and the proportion of 'FA' trials (FA rate; x axis) separately for trials with coherence levels = 0.03, 0.06, 0.12, and 0.25 (see Figure S2A for a representative participant: the four dots on the line represent the proportion of Hit over FA trials for each of these four coherence levels). Participants chose the internal task option as the coherence of the internal task option increased (see also Figure 3A). We then connected these points and defined the area under the curve as type-II A_{ROC}. The higher the proportion of hit trials compared to false alarm trials, the more closely type-II A_{ROC} approaches 1. Chance level of type-II A_{ROC} is 0.5.

We used a similar approach to evaluate perceptual performance (Figures 3B, 6, and 7C): we used type-I A_{ROC} based on the proportion of trials in which participants correctly judged the motion direction as 'left' [or 'down'] when the dots were moving leftward [downward] (Hit trials) and that they misjudged the motion direction as 'left' ['down'] when the dots were moving rightward [upward] (FA trials). We calculated type I A_{ROC} with the formula below:

$$Z(type\ I\ A_{ROC}) = \frac{Z(Hit\ rate) - Z(FA\ rate)}{\sqrt{2}}$$

The higher the proportion of hit trials compared to false alarm trials, the more closely type I A_{ROC} approaches 1. Chance level of type I A_{ROC} is also 0.5.

To evaluate metacognitive performance by controlling the differences in perceptual performance, we calculated the metacognitive efficiency score measured by M ratio below:

$$M\ ratio = \frac{meta - d'}{d'} = \frac{\sqrt{2}\cdot Z(type\ II\ A_{ROC})}{\sqrt{2}\cdot Z(type\ I\ A_{ROC})}$$

To evaluate the effects of internal and external probability on task selection based on metacognitive judgment and the following perceptual decision performance, we employed logistic multiple regression analyses as shown below (Figures 3A, 3C, S2D, and S2E).

$$\ln\left(\frac{y(n)}{1-y(n)}\right) = \alpha + \beta_{int}x_{int}(n) + \beta_{ext}x_{ext}(n) + \sum_{k=1}^{3}\Big(\beta_{o\_int}o_{int}(n-k) + \beta_{o\_ext}o_{ext}(n-k) + \beta_{int}x_{int}(n-k) + \beta_{ext}x_{ext}(n-k)$$
$$+ \beta_{int\cdot o\_int}o_{int}(n-k)\cdot x_{int}(n-k) + \beta_{ext\cdot o\_ext}o_{ext}(n-k)\cdot x_{ext}(n-k)\Big)$$

Dependent variable y(n) denotes the task chosen during the metacognitive judgment stage (internal task = 1; external task = 0) (Figures 3A and S2D) or performance during the perceptual task stage (correct/rewarded = 1; incorrect/rewarded = 0) at the trial #n (Figures 3C and S2E). Independent variables $x_{int}(n)$ and $x_{ext}(n)$ denote the internal probability and external probability at trial #n, respectively. Internal probability corresponds to the coherence of the motion in the internal task option but linearly transformed from its original exponential scale. External probability is equal to the probability of reward offered by the external task option. To capture the effects during previous trials, we included the outcomes of internal and external probability tasks during the past three encounters; $o_{int}(n)$ and $o_{ext}(n)$ denote the outcome (correct/rewarded = 1; incorrect/rewarded = 0) for internal and external task performances on trial #n, respectively. We also included the previous external and internal probabilities, as well as the interaction of previous outcomes and probabilities separated by external and internal task (sum over k = 1 to k = 3). All the independent variables are normalized (mean of zero and standard deviation of one) within each session before including them into the analysis.

To evaluate the effect of the decision variable (DV; the difference between the expected reward probability of choosing the internal task option versus the external option) on the chosen task during the metacognitive judgment stage, we applied a logistic regression analysis as follows (Figures S2B and S8B).

CellPress
OPEN ACCESS

$$\ln\left(\frac{y(n)}{1-y(n)}\right) = \alpha + \beta DV(n)$$

DV(n) is defined by subtraction, at trial #n, of the external reward probability, associated with the external option, from the internal reward probability (based on the reward frequency recorded for each coherence level in the follow-up task performed by each participant) associated with the internal option.

We also calculated subjective utility functions for internal [or, external] probability based on subjective probability (*w(p)*; the proportion of trials on which the internal [or, external] option was chosen in the metacognitive judgment stage) and objective probability (*p*; the proportion of the trials that internal [or, external] probability option should have been chosen if participants behaved optimally; i.e., if they always picked the better probability option in the metacognitive judgment stage) (Figures S1C–S1F). The data are fitted by typical subjective utility functions based on Prospect Theory (Kahneman and Tversky, 1979) as follows, where skewness is defined by a single parameter γ:

$$w(p) = \frac{p^{\gamma}}{\left(p^{\gamma} + (1-p)^{\gamma}\right)^{\frac{1}{\gamma}}}$$

It is noted that smaller gamma indicates larger distortion and, if the participant has a perfect undistorted utility function, gamma comes close to 1 (log gamma comes close to 0). In both internal and external probabilities, as previously observed and consistent with the predictions of Prospect Theory (Kahneman and Tversky, 1979), there is subjective overestimation of lower probabilities and subjective underestimation of higher probabilities. Comparison of skewness (gamma) suggests that there is nothing fundamentally different about the way in which observations of real 'objective' frequencies of success in internal and external tasks are translated into subjective estimates of success in the two cases ($t_{22} = 1.23$, $p = 0.23$; Figure S1D). We also confirmed another fundamental aspect of the subjective expectations described in Prospect Theory, risk aversion, operated similarly in both the internal and external probability domains (overall reward rates: internal option, 75.7% ± 1.0%; external option, 73.6% ± 1.4%; $p = 0.27$. outcome variances: internal option, 0.42 ± 0.006; external option, 0.43 ± 0.007; $p = 0.36$) (Platt and Huettel, 2008; Preuschoff et al., 2006; Rushworth and Behrens, 2008).

Functional MRI data

Whole-brain analysis

We used FSL FEAT for first-level analysis. First, data were pre-whitened with FSL FILM to account for temporal autocorrelations. Temporal derivatives were included into the model. We used three fMRI general linear models (fMRI-GLM1, 2, 3) to analyze fMRI data across the whole brain. Results were calculated using FSL's FLAME 1 with a cluster-correction threshold of $z > 3.1$ and $p < 0.05$, two-tailed.

To analyze BOLD changes across participants, a second-level analysis was applied in a two-step approach: two functional MRI sessions (Sessions 4 and 5) in Experiment 1 were first averaged within subject (fixed-effect analysis) and then sessions were analyzed across participants (FLAME1). We used two covariate fMRI analyses (fMRI-GLM2 and fMRI-GLM3) during which we associated a covariate with a particular regressors in the second level (FLAME 1).

All whole brain GLMs shared the following features: we included all three phases of a trial (metacognitive judgment, perceptual decision, and outcome) into the fMRI-GLMs. Each phase included a constant regressor, which was the onset of each phase with a fixed duration of 1.5 s for metacognitive judgment and perceptual decision and a duration of 1 s for the outcome phase. Parametric regressors were modeled as stick functions (i.e., duration of zero) time-locked to the relevant phase onset as below. All parametric regressors were normalized before inclusion into the analysis. In addition, all GLMs contained one regressor time-locked to all button presses, modeled as a stick function, at the first-level fixed-effect analysis stage.

fMRI-GLM1. First, we tested for neural correlates of internal and external probabilities during the metacognitive judgment stage (Figures 5 and S4–S6). We included the following regressors, along with the constant regressor coding the phase of metacognitive judgment in each trial #n, to do this:

Chosen Internal probability,
Chosen External probability,
Unchosen Internal probability,
Unchosen External probability,
Outcome of chosen Internal task at trial #(n-1) [1 (correct) or 0 (incorrect)],
Outcome of chosen External task at trial #(n-1) [1 (rewarded) or 0 (unrewarded)].

All regressors were normalized before inclusion into the analysis (mean of zero and standard deviation of one). If participants chose the internal task on trial #n, then the internal probability of the internal task option and the external probability of the external task option were coded as chosen internal probability and unchosen external probability, respectively. These variables were time-locked to the metacognitive judgment stage when participants chose the internal task. Chosen external probability and unchosen internal probability were not defined for those trials. If participants chose the external task on trial #n, the external probability of the external

task option and the internal probability of the internal task option were coded as chosen external probability and unchosen internal probability, respectively. These variables were time-locked to the metacognitive judgment stage when participants chose the external task. Both the chosen internal probability and unchosen external probability were not defined for those trials. Similarly, the outcome variable for the last trials #(n-1) was defined for the respective chosen task: for example, if participants chose the internal task during the last trial, then the external outcome was not defined. To identify neural activity that reflected the differences in chosen and unchosen probabilities, we calculated the difference between the sum of chosen and unchosen probability differences '(chosen - unchosen Internal probability) + (chosen - unchosen External probability)' (Figure S4A). To identify neural activity that reflected internal probability or external probability irrespective of whether the task was chosen or unchosen, we calculated the following two contrasts: 'chosen + unchosen Internal probability' (Figure 5A) and 'chosen + unchosen External probability', respectively. We also derived the difference of these contrasts: '(chosen + unchosen Internal probability) - (chosen + unchosen External probability)' (Figures S5C and S5D). We also conducted a conjunction analysis to identify activity that reflected both 'chosen + unchosen Internal probability' and 'chosen + unchosen External probability' with $z > 3.1$ and $p < 0.05$ (Figure S4D).

To identify activity related to making decisions about the directions of stimuli during the perceptual decision stage (Figures 4 and S3), we used the following regressors for the perceptual decision stage:

Chosen Internal probability,
Chosen External probability,
Unchosen Internal probability,
Unchosen External probability.

All regressors were normalized before inclusion into the analysis. The same parametric predictors as those coded in the metacognitive judgment stages were used. If participants chose the internal task on trial #n, then the internal probability of the internal task option and the external probability of the external task option were coded as chosen internal probability and unchosen external probability, respectively. These variables were time-locked to the perceptual decision stage when participants chose the internal task. Chosen external probability and unchosen internal probability were not defined for those trials. If participants chose the external task on trial #n, the external probability of the external task option and the internal probability of the internal task option were coded as chosen external probability and unchosen internal probability, respectively. These variables were time-locked to the perceptual decision stage when participants chose the external task. Both the chosen internal probability and unchosen external probability were not defined for those trials. We confirmed, however, that the predictors in the metacognitive judgment stage and perceptual decision stage were independent of each other (maximum r value was 0.2; see Figures S3A and S3B for correlation between regressors and trial stages), and therefore were able to identify separate portions of the variance in neural activity. The correlation across stages was minimized because there was a temporal jitter between the onsets of the two stages. The duration of the temporal jitter was drawn from a Poisson distribution with the range of 4 s to 10 s and a mean of 4.5 s (as the duration of each stimulus presentation was fixated to 1.5 s, SOA between the two stages was in the range of 2.5 s to 8.5 s). During these intervals, a fixation cross was shown on the screen. To identify neural activity reflecting internal probability or reflecting external probability, we calculated the contrasts of 'chosen Internal probability' (Figure 4B) and 'chosen External probability', respectively. We also conducted a conjunction analysis (Nichols et al., 2005) to identify activity that reflected both 'chosen Internal probability' and 'chosen External probability' with $z > 3.1$ and $p < 0.05$ (Figure 4A).

In order to capture activity related to the outcome of each decision, the outcome phase included the following regressors:

Outcome of chosen Internal task [1 (correct) or 0 (incorrect) time-locked to internal task outcomes],
Outcome of chosen External task [1 (rewarded) or 0 (unrewarded) time-locked to external task outcomes].

The outcome variable was defined for the task chosen: for example, if participants chose the internal task, then the external outcome was not defined.

fMRI-GLM2: covariate analysis at metacognitive judgment stage. Next, we were interested whether signals associated with the contrasts 'chosen - unchosen internal probability' during the metacognitive judgment stage covaried with individual Type II A_{ROC} values (Figures 6B and 6C, left panels). We included Type II A_{ROC} values as covariates at the third stage of group analysis when averaging across participants (FLAME 1). We used the same fMRI analysis described in fMRI-GLM1, but now included additionally both 'type II A_{ROC} (metacognitive judgment performance)' and 'type I A_{ROC} (perceptual decision performance)' as covariates. We calculated A_{ROC} of each participant for each session and then averaged across sessions; the averaged value was included as covariate.

fMRI-GLM3: covariate analysis at perceptual decision stage. Next, we were interested whether signals associated with the contrasts 'chosen - unchosen internal probability' during the perceptual decision stage covaried with individual Type I A_{ROC} (Figures 6B and 6C, right panels). We included individual values as covariates at the third stage of group analysis when averaging across participants (FLAME 1). We used the same fMRI analysis described in fMRI-GLM1, but now included additionally both 'type II A_{ROC} (metacognitive judgment performance)' and 'type I A_{ROC} (Perceptual decision performance)' as covariates.

Region of interest (ROI) analyses

We calculated ROIs with a radius of three voxels that were centered on the peak voxel of significant clusters derived from whole brain fMRI-GLM1. The selected ROI was transformed from MNI space to subject space and the pre-processed BOLD time courses were

CellPress
OPEN ACCESS

extracted for each participant's session. Time courses were averaged across volumes, then normalized and oversampled by a factor of 20 for visualization. ROI-GLMs were applied to each time point to derive beta weights per time point for each regressor. For analyses across conditions, we used the same principle as applied to the whole-brain fMRI-GLM1: first, we averaged the time course within a subject across two fMRI sessions, then we averaged across the group. For all ROI analyses, regressors were normalized (mean of zero and standard deviation of one). For all time course analyses, we used the same parametric predictors described in the whole-brain fMRI analysis conducted with fMRI-GLM1 for the phases of metacognitive judgment stage (Figures 5B, S3E, S4B, S4E, S5A, S5D, and S6C), perceptual decision stage (Figures 4C, S3D, S6B, and S6D), and outcome phase. We also time-locked the time courses to the same phase onsets as described in fMRI-GLM1.

Analysis for the slope of time course of neural beta weight

For each participant, we extracted a time course from alPFC time-locked to the metacognitive stage and included two parametric regressors: chosen Internal probability and unchosen Internal probability (we will refer to this analysis as ROI-GLM1). Note, that the chosen and unchosen internal probability variables are time-locked to trials where participants chose the internal or external task, respectively. The time courses either started from the onset of the metacognitive judgment stage or the onset of perceptual decision stage (respectively, Figures 5B, left, and 5B, right). The coordinates of alPFC ROIs were determined by the bilateral peak-coordinate of fMRI-GLM1 associated with the contrast 'chosen + unchosen Internal probability' in the metacognitive judgment stage. We averaged the time course of the left and right alPFC for each participant. Next, we assessed the time point at which the beta weight was at its minimum [t_{min}, β_{min}] (note that 0 s < t_{min} < 4 s; perceptual decision stage did not start for sure within this range [see purple bar in Figure 5B]) and the time point at which the beta weight was at its maximum [t_{max}, β_{max}] (note that 0 s < t_{max} < 11 s and $t_{min} < t_{max}$). Then, we calculated the slope for each fMRI session with the following formula:

$$slope^{alPFC}_{chosen\ or\ unchosen} = \frac{\beta_{max} - \beta_{min}}{t_{max} - t_{min}}$$

We averaged the slope of the two fMRI sessions within each participant, separately for chosen Internal probability ($slope^{alPFC}_{chosen}$) and for unchosen Internal probability ($slope^{alPFC}_{unchosen}$). Then we compared these slopes across participants in the metacognitive judgment stage (Figure 5C) and perceptual decision stage (Figure S5B). We also calculated the correlation between the difference in slopes ($slope^{alPFC}_{chosen} - slope^{alPFC}_{unchosen}$) at the metacognitive judgment stage and the metacognitive performance index (type II A_{ROC}) across participants (Figure 6A, upper left panel). Slopes in effect sizes albeit in other brain areas, reflecting other decision variables, and manifesting in distinct ways have previously been linked to neural evidence accumulation and individual differences in the representation of evidence strength (Wittmann et al., 2016a).

Similarly, we extracted β_{min} and β_{max} from the FPm ROI time course and calculate the difference as follows:

$$magnitude^{FPm}_{chosen\ or\ unchosen} = \beta_{max} - \beta_{min}$$

The coordinates for FPm were determined by the peak detected by the contrast 'chosen Internal probability' in the perceptual decision stage in whole brain fMRI-GLM1. We calculated the correlation between the difference in beta value ($maginitude^{FPm}_{chosen} - magnitude^{FPm}_{unchosen}$) at the perceptual decision stage and the perceptual decision performance index (type I A_{ROC}) across participants (Figure 6A, lower right panel).

Neuron, Volume *109*

Supplemental information

Identification and disruption of a neural mechanism for accumulating prospective metacognitive information prior to decision-making

Kentaro Miyamoto, Nadescha Trudel, Kevin Kamermans, Michele C. Lim, Alberto Lazari, Lennart Verhagen, Marco K. Wittmann, and Matthew F.S. Rushworth

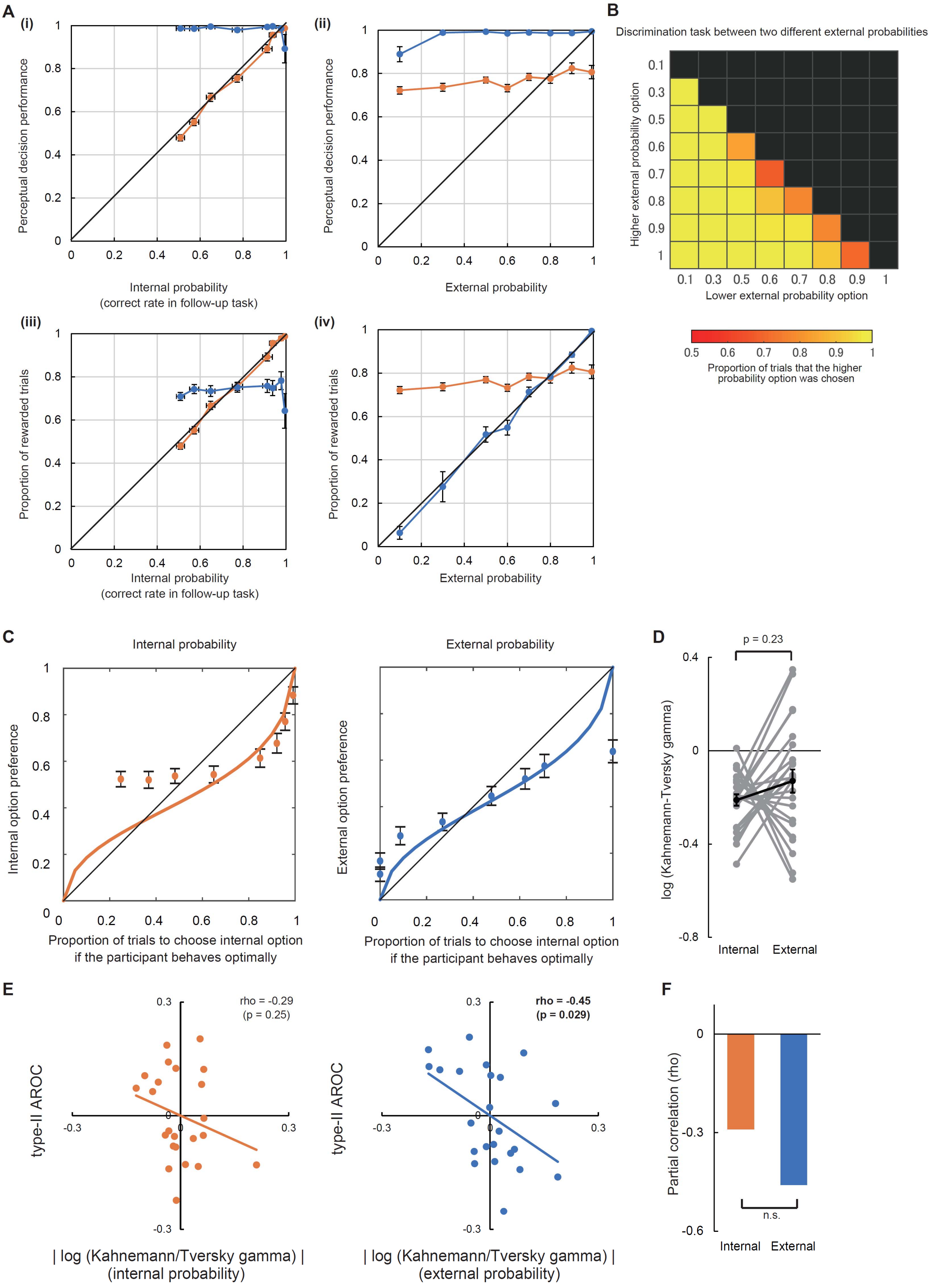
A
(i)
Perceptual decision performance
Internal probability
(correct rate in follow-up task)
(ii)
External probability
B
Discrimination task between two different external probabilities
Higher external probability option
Lower external probability option
Proportion of trials that the higher probability option was chosen
(iii)
Proportion of rewarded trials
(iv)
C
Internal probability
External probability
Internal option preference
External option preference
Proportion of trials to choose internal option if the participant behaves optimally
D
p = 0.23
log (Kahnemann-Tversky gamma)
Internal
External
E
type-II AROC
rho = -0.29
(p = 0.25)
rho = -0.45
(p = 0.029)
| log (Kahnemann/Tversky gamma) |
(internal probability)
(external probability)
F
Partial correlation (rho)
n.s.

Supplemental Figure 1. Comparative subjective utility functions for internal and external probabilities (related to Figure 2)

(A) (i) Proportion of correct trials (perceptual decision performance in the main task) in the second decision stage as a function of internal probability (based on follow-up task performance at each coherence level) offered in the first metacognitive judgment stage. The trials on which the internal probability option was chosen (orange) and on which the alternative, the external probability option was chosen (blue) are summarized independently. Note that the probability of correctly discerning the motion direction tightly scaled with the internal probability estimated from the follow-up task. By contrast, discerning the correct motion direction was trivially easy when opting for the external probability task and hence performance was at ceiling independent of what had been offered as the internal probability task. **(ii)** Proportion of correct trials in the second decision stage as a function of external probability. The trials on which the external probability option was chosen (blue) and unchosen (orange, i.e., internal probability option was chosen) are summarized independently. Note, again, that the quantity plotted on the y-axis related to the correct estimation of the dot direction, and that, after choosing the internal task, reward would always deterministically ensue after a correct estimation, whereas after choosing the external task and estimating the dot direction correctly, reward would only ensue with a given probability, i.e. with the external probability. **(iii– iv)** Same convention with as (i–ii) but summarized for proportion of rewarded trials instead of correct trials. Note that here, when comparing (iii) and (iv), it becomes apparent that the actual payoff from choosing each task tightly scales with internal and external probability, respectively, while the value of the alternative option is held constant. Together, this figure illustrates how the overall payoff was thus finely balanced between tasks, but that they were critically different with respect to how difficult it was to perform the tasks correctly in order to arrive at this payoff. On the top (i–ii) and bottom (iii–iv) panels, the lines for the trials on which the internal probability option was chosen (orange) are exactly the same because external probability was always set at 100% for internal probability options. **(B)** Performance on an additional control task in which participants compared two different levels of external probability. The colour within each cell indicates the probability with which participants chose the optimal external task. The optimal external task is simply the external task with a higher probability of reward, i.e., higher number of dots. The participants (n=4) could reliably choose the option with higher external probability (i.e. larger number of dots) (92.5±1.5

[mean±s.e.m.]%). (**C**) Subjective utility functions for internal (left) [or, external (right)] probability. Subjective probability (y-axis) is the proportion of trials that on which the internal [or, external] option was chosen in the metacognitive judgment stage. Objective probability (x-axis) is the proportion of the trials that the internal [or, external] probability option should have been chosen if participants behaved optimally (i.e., if they always picked the better probability option in the metacognitive judgment stage). Each dot is a given level of internal/external probability and increases from left to right (Note that the two dots on the y axis in the right panel indicate the data for 10% [lower] and 30% [higher] external probability, respectively). The data are fitted by typical subjective utility functions based on Prospect Theory (Kahneman and Tversky, 1979), where skewness is defined by a single parameter: gamma. (**D**) Comparative gamma between internal and external probabilities ($p = 0.23$) suggests that there is nothing fundamentally different about the way in which objective probabilities are translated into subjective estimates. It is noted that smaller gamma indicates larger distortion and, if the more the participant has a perfect undistorted utility function, the closer gamma comes close to 1 (log gamma comes close to 0). **(E)** Multiple regression analysis reveals that if a participant has a gamma closer to 1 for either internal (left) or external (right) probability then this predicts higher type-II A_{ROC} in the same participant. **(F)** Comparable sizes of gamma for both internal and external probabilities suggests that both probabilities contribute similarly to type-II A_{ROC}.

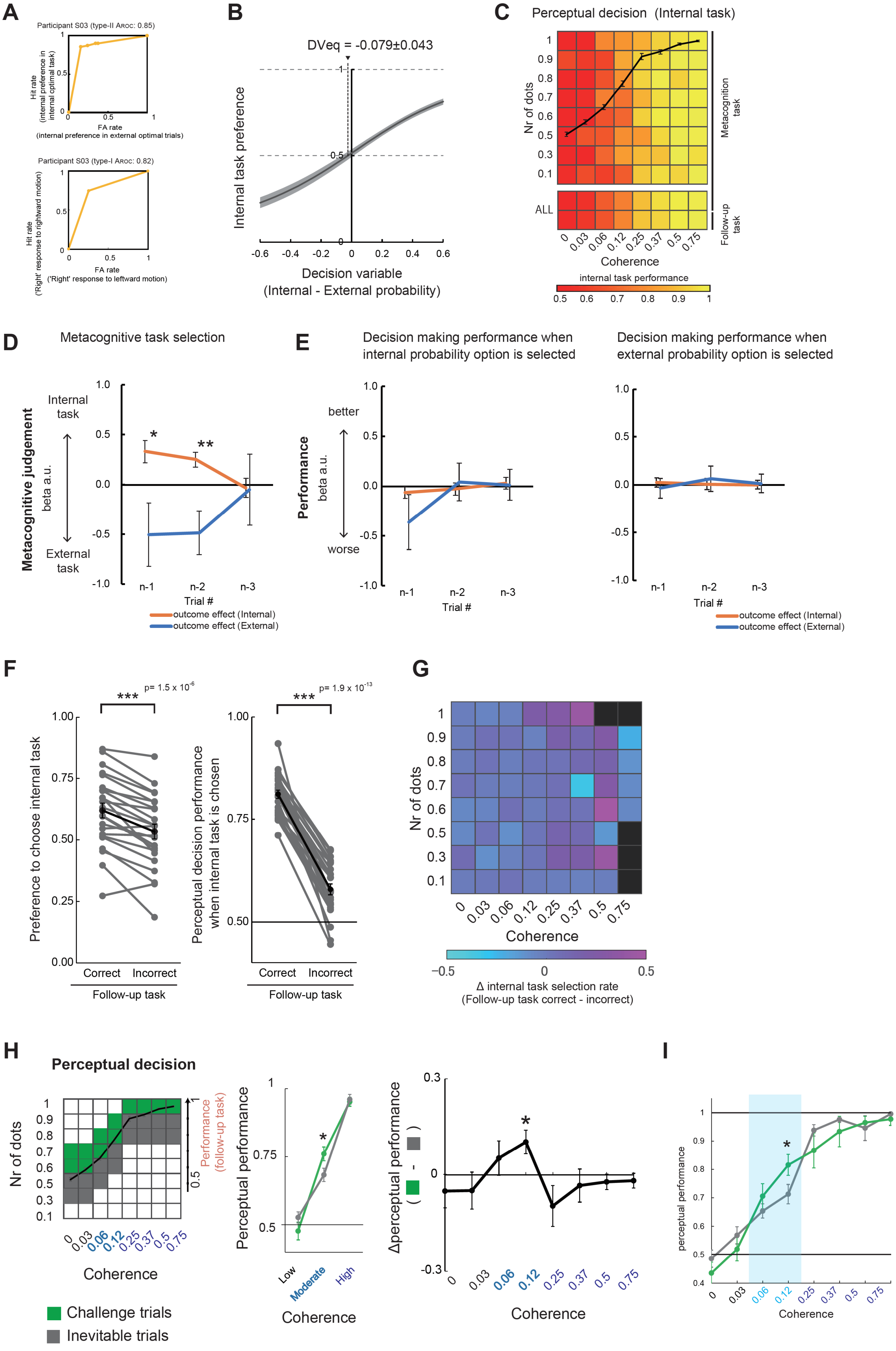
A
Participant S03 (type-II AROC: 0.85)
Hit rate (internal preference in internal optimal task)
FA rate (internal preference in external optimal trials)
Participant S03 (type-I AROC: 0.82)
Hit rate ('Right' response to rightward motion)
FA rate ('Right' response to leftward motion)
B
DVeq = -0.079±0.043
Internal task preference
Decision variable
(Internal - External probability)
C
Perceptual decision (Internal task)
Nr of dots
Metacognition task
ALL
Follow-up task
Coherence
internal task performance
D
Metacognitive task selection
Metacognitive judgement
beta a.u.
Internal task
External task
Trial #
outcome effect (Internal)
outcome effect (External)
E
Decision making performance when internal probability option is selected
Decision making performance when external probability option is selected
Performance
beta a.u.
better
worse
Trial #
outcome effect (Internal)
outcome effect (External)
F
p= 1.5 x 10^-6
p= 1.9 x 10^-13
Preference to choose internal task
Perceptual decision performance when internal task is chosen
Correct
Incorrect
Follow-up task
G
Nr of dots
Coherence
Δ internal task selection rate
(Follow-up task correct - incorrect)
H
Perceptual decision
Nr of dots
Performance (follow-up task)
Coherence
Challenge trials
Inevitable trials
Perceptual performance
Low
Moderate
High
Δperceptual performance
I
perceptual performance
Coherence

Supplemental Figure 2. Behavioral performance during prospective metacognition task (related to Figure 3).

(A) The type-II and type-I ROC curves of a representative participant (indicated with a black circle in the upper plot). The dots on the line represent the proportion of Hit over FA trials for specific coherence levels (see STAR Methods). **(B)** Preference for choosing the internal probability task as a function of decision variable (DV – the difference between the internal and external probabilities). **(C)** Performance of the internal probability task during the perceptual decision stage in the main metacognition task (where each perceptual decision was preceded by a metacognitive judgment). Performance in the follow-up task (which lacked any metacognitive judgment phase) is indicated by the black line in the square plot and color-coded in the rectangular plot below. Average performance across all probability levels is shown for the metacognitive judgment stage (upper bar) and follow up-task (lower bar) at the bottom of the panel. **(D–E)** There was a significant influence of outcome (reward/no-reward) received during the past three trials on the choice made in the metacognitive judgment stage (D). There was no influence of outcome (reward/no-reward) received during the past three trials on the performance of the internal task (E, left) and external task (E, right). Metacognitive judgments were also influenced by the outcomes of decisions made on previous trials. Whether or not reward was received for taking an internal probability option and for taking an external probability option had impacts on subsequent metacognitive judgments. The impacts were in opposite directions (two-way repeated ANOVA with the main effects of evidence type and time constant; main effect of internal/external evidence, $F_{1,22}$=7.65, p=0.011; main effect of time constant, $F_{2,44}$=0.06, p=0.94; interaction, $F_{2,44}$=2.46, p=0.096). The receipt of reward outcomes when internal probability options had been chosen recently increased the likelihood of selecting the internal probability in a subsequent metacognitive decision (one-way repeated ANOVA with the main effects of time constant, $F_{2,44}$=6.27, p=0.0040; paired t-test: $trial_{n-1}$ > $trial_{n-3}$, p=0.001, $trial_{n-2}$ > $trial_{n-3}$, p=0.011 with Bonferroni correction. $trial_{n-1}$, t_{22}=2.95, p=0.022; $trial_{n-2}$, t_{22}=3.36, p=0.0085, t-test against zero with Bonferroni correction; note that participants chose internal probability options on approximately 50% of trials). However, external probability options chosen more recently did not change the likelihood of selecting the external probability option significantly (one-way repeated ANOVA with the main effects of time constant, $F_{2,44}$=0.81, p=0.45; $trial_{n-1}$, t_{22}=-1.58, p=0.38; $trial_{n-2}$, t_{22}=-2.21, p=0.11, t-test against zero with Bonferroni correction). This difference also suggested that there are

independent mechanisms for evaluating and learning about internal and external probabilities. **(F)** Preference, in the metacognitive judgment stage of the main task, for choosing to perform the internal task trials as a function of whether the same trial would be performed correctly in the follow-up task. Left panel: First, we categorized metacognitive trials into correct and incorrect trials according to performance on the follow-up task. Second, we calculated the proportion of occasions the participant chose the internal vs external probability task option in the correct trial group and the incorrect trial group. Here, we plot the proportion of internal task choices when follow-up task performance was correct (left side of the x-axis) or incorrect trials (right side on the x-axis). Participants more often chose the internal probability option in the metacognitive judgment phase in the main experiment when they would subsequently classify motion direction correctly in the follow-up task (the average across participants, indicated as dots, is above 50% which represents a preference for the internal task) (t_{22}=6.49, p=1.5×10^{-6}) Right panel: The performance of the chosen internal probability task was also better in the perceptual decision stage in the main experiment for options that participants would subsequently classify correctly when they encountered them in the follow-up task (t_{22}=15.7, p=1.9×10^{-13}). **(G)** The difference in preference for choosing the internal probability option for options that were classified correctly in the follow-up task as opposed to those that were not classified correctly in the follow-up task. **(H)** It was confirmed that perceptual decision performance on the internal task at moderate coherence levels (0.06, 0.12) was higher when the internal task had been paired with an external probability option that was, on average, slightly more likely to yield reward (Challenge trials) than it was when the internal task was paired with an external probability option slightly less likely to yield reward on average (Inevitable trials) by defining Challenge and Inevitable trials as the trials two cells above and below the curve (two-way ANOVA, trial-type [Challenge, Inevitable] × coherence level [Low, Moderate, High]. Interaction: p=0.0166. Simple main effect for Challenge vs. Inevitable trials at moderate coherence: p=0.027). Again, this suggests that participants are able to estimate their likelihood of success if they perform the internal probability task. The black line in the square plot indicates performance in the follow-up task. **(I)** Perceptual decision performance on the internal task for Challenge trials (green) and Inevitable trials (grey) for each coherence level. For the two levels of moderate coherence (0.06 and 0.12) (blue shade), a two-way ANOVA (coherence [0.06 or 0.12] × trial type [Challenge or Inevitable]) revealed a significant main effect of Challenge/Inevitable trial type (p = 0.023) and a main effect of coherence (p = 0.037)

without any interaction ($p = 0.45$). (N=23 participants; *$p<0.05$, **$p<0.01$, ***$p<0.001$, t-test against chance-level, Bonferroni correction if required; error bars are SEM across participants).

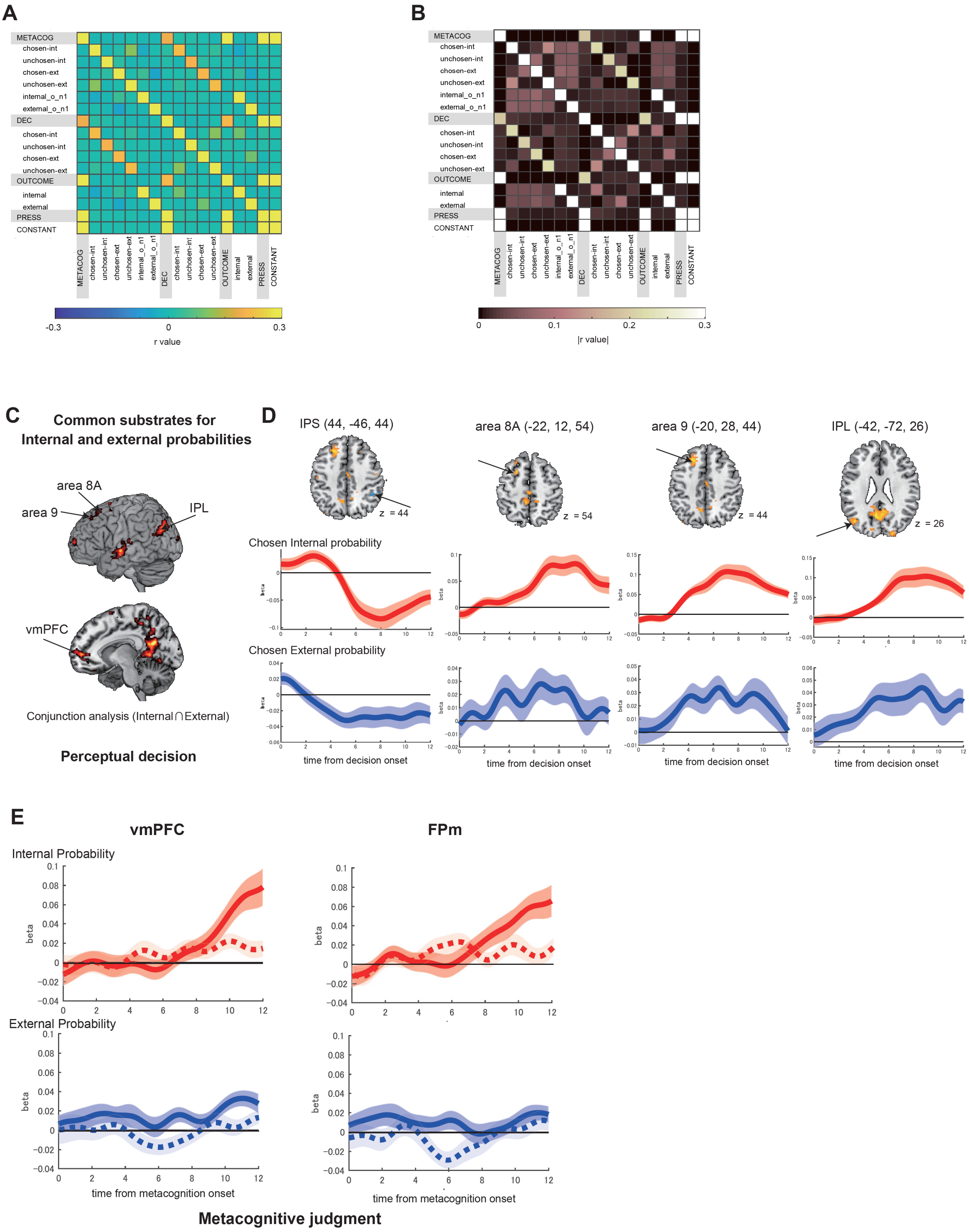

Supplemental Figure 3. Perceptual decision-making: common substrates for internal and external evidence accumulation in well-known areas for motion vision (related to Figure 4)

(A–B) Correlation between regressors included in the whole-brain fMRI and ROI analyses. Predictors included in fMRI-GLM1 and ROI-GLM1 (we included the same regressors in both analyses) between the metacognitive judgment stage and perceptual decision stage had a reasonable low correlation (maximum r value of 0.2). Although we used the same variables in both stages, the correlation between regressors was decreased by including a temporal jitter (see STAR Methods for details). The mean of the correlation coefficients (A) and the mean of the absolute correlation coefficients (B) are shown. **(C)** Activity in several frontal and parietal areas reflected the probability of reward associated with the chosen action – the evidence for making the chosen action – regardless of whether that was derived from consideration of internal or external probabilities at a liberal statistical criterion for display purpose (whole-brain effects family-wise error cluster corrected with $z>2.3$ and $p<0.05$). **(D)** The evolution of the regression weights across time indexing the impact of chosen internal probability (red at top) and chosen external probability (blue at bottom) on neural activity are illustrated for four example areas (IPS, area 8A, area 9, IPL). In each case the effects are seen after a short delay reflecting the hemodynamic response function. **(E)** The evolution of the regression weights across time in the metacognitive judgment stage indexing the impact of internal probability (red at upper panel of each area. solid line: chosen internal probability. dotted line: unchosen internal probability) and chosen external probability (blue at lower panel of each area. solid line: chosen external probability. dotted line: unchosen external probability) on neural activity are illustrated for two example areas: vmPFC and FPm.

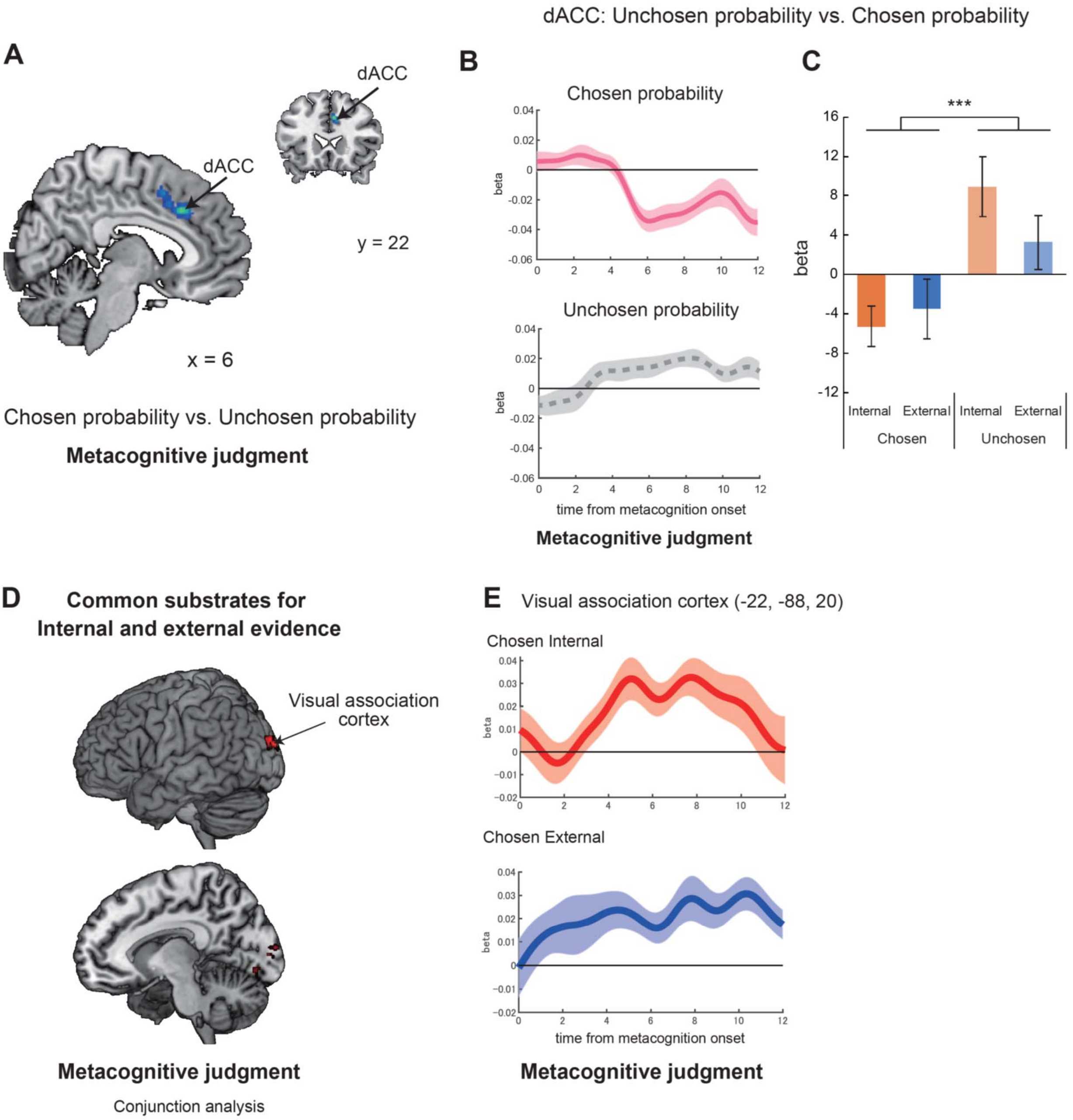

Supplemental Figure 4. Metacognitive judgment: common substrates for internal and external evidence accumulation in the anterior cingulate cortex and visual association cortex (related to Figure 5).

(A) Activity in dACC reflected both internal and external probabilities during the initial metacognitive decision on each trial. **(B)** Activity in dACC was positively modulated by probabilities associated with the option that was rejected during the metacognitive judgment, while its activity was negatively modulated by probabilities associated with the option that was taken. Similar patterns of activity change in dACC have been linked previously to the

evidence for taking an alternative or counterfactual choice as opposed to the choice actually made (Fouragnan et al., 2019; Kolling et al., 2018; Kolling et al., 2016). **(C)** This pattern of activity increase and decrease in association, respectively, with choices rejected and choices taken was apparent during the assessment of both internal and external probabilities during metacognitive decisions (two-way repeated ANOVA with the main effects of evidence and chosen task; main effect of internal/external probability, $F_{1,22}$=1.06, p=0.31; main effect of chosen/unchosen task, $F_{1,22}$=23.0, p=0.001; interaction, $F_{1,22}$=1.10, p=0.30). **(D)** Visual association cortex is the only area where both the internal and external probabilities were coded in a similar manner during the metacognitive judgment stage. **(E)** The evolution of the regression weights across time indexing the impact of internal probability (red at top) and external probability (blue at bottom) on neural activity in visual association cortex. (N=23, whole-brain effects family-wise error cluster corrected with z>3.1 and p<0.05; shade and error bars indicate SEM across participants; *** p<0.001, main effect of ANOVA).

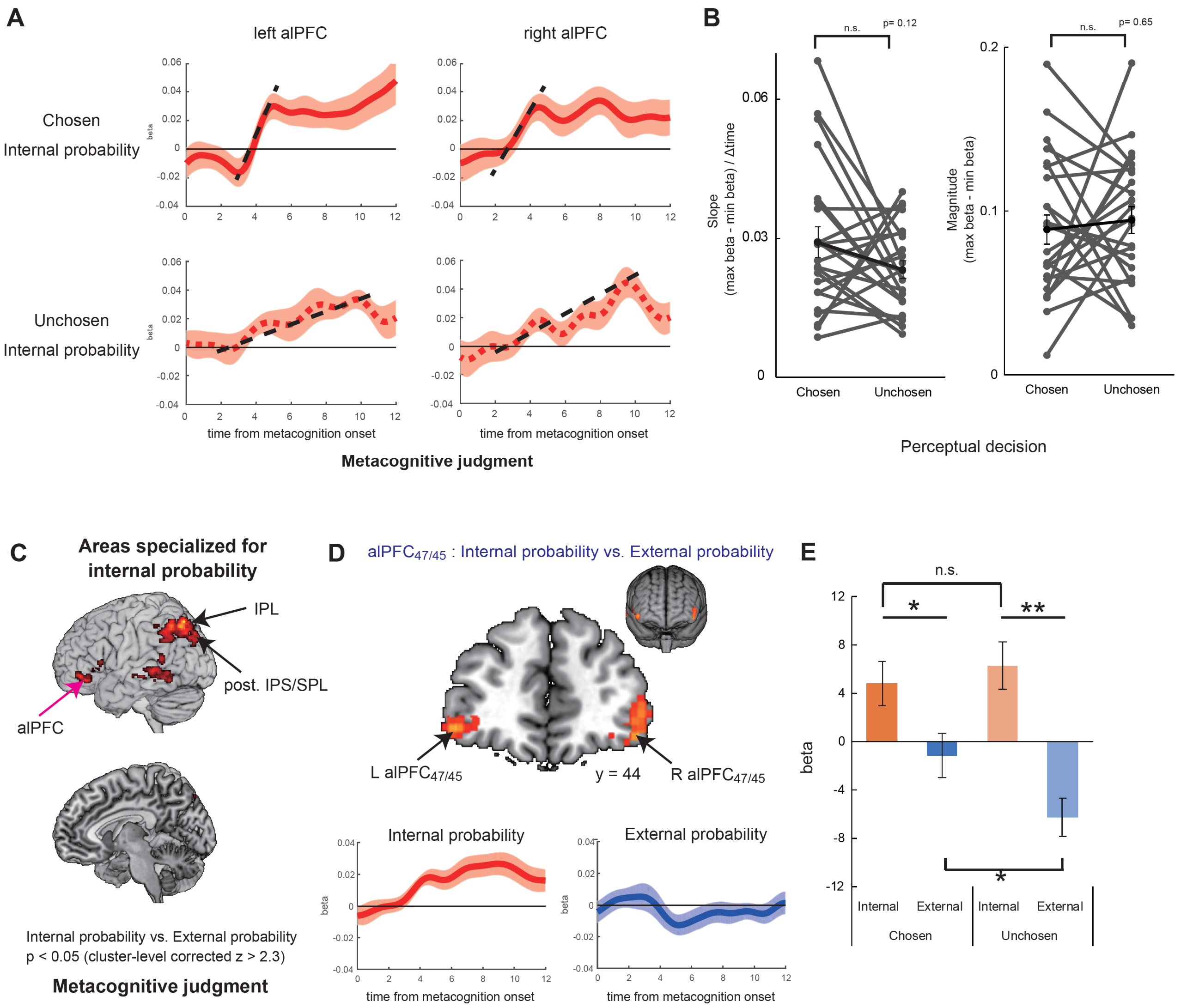

Supplemental Figure 5. Evidence accumulation of internal probability but not of external probability during metacognitive judgment in alPFC (related to Figure 5).

(A) Internal probability associated with both chosen (top) and rejected (bottom) options exerted a positive influence in a similar manner on both left and right alPFC$_{47}$. **(B)** Unlike in metacognitive judgments, there was no significant difference in slope (left) or the size of the peak signal (right) for activity related to the chosen and unchosen internal probability task options during perceptual decisions (see also Figure 5C). **(C)** Activity in alPFC$_{47/45}$ was significantly more strongly modulated by the internal probability than the external probability of chosen and unchosen options during the metacognitive judgment stage, illustrated at a liberal statistical criterion for display purpose (whole-brain effects family-wise error cluster corrected with $z>2.3$ and $p<0.05$). There was a clear region of difference within

alPFC that peaked adjacently ([x, y, z]=[-48, 44, -8], [48, 44, -12]) lying on the boundary between areas 45 and 47/12 (which we therefore refer as $alPFC_{47/45}$). **(D)** The $alPFC_{47/45}$ modulation in relation to internal as opposed to external probability is also evident in the time courses of the effects. **(E)** Unlike in other areas linked to decision making in fMRI investigations, activity modulation in $alPFC_{47/45}$ was positively modulated by the internal probability associated with both the chosen and the unchosen option. While the external probability associated with a chosen option had no impact on $alPFC_{47/45}$ activity, the external probability associated with an option that was rejected led to a decrease in $alPFC_{47/45}$ activity. This was apparent when the activity was analyzed with a two-way repeated measures ANOVA with factors of evidence type (internal/external probability) and option chosen (chosen or unchosen task); there was a significant main effect of internal/external probability ($F_{1,22}$=18.26, p=0.0003) but no main effect of chosen/unchosen task ($F_{1,22}$=0.56, p=0.46) or interaction ($F_{1,22}$= 3.30, p=0.082). (N=23, shade indicates SEM across participants).

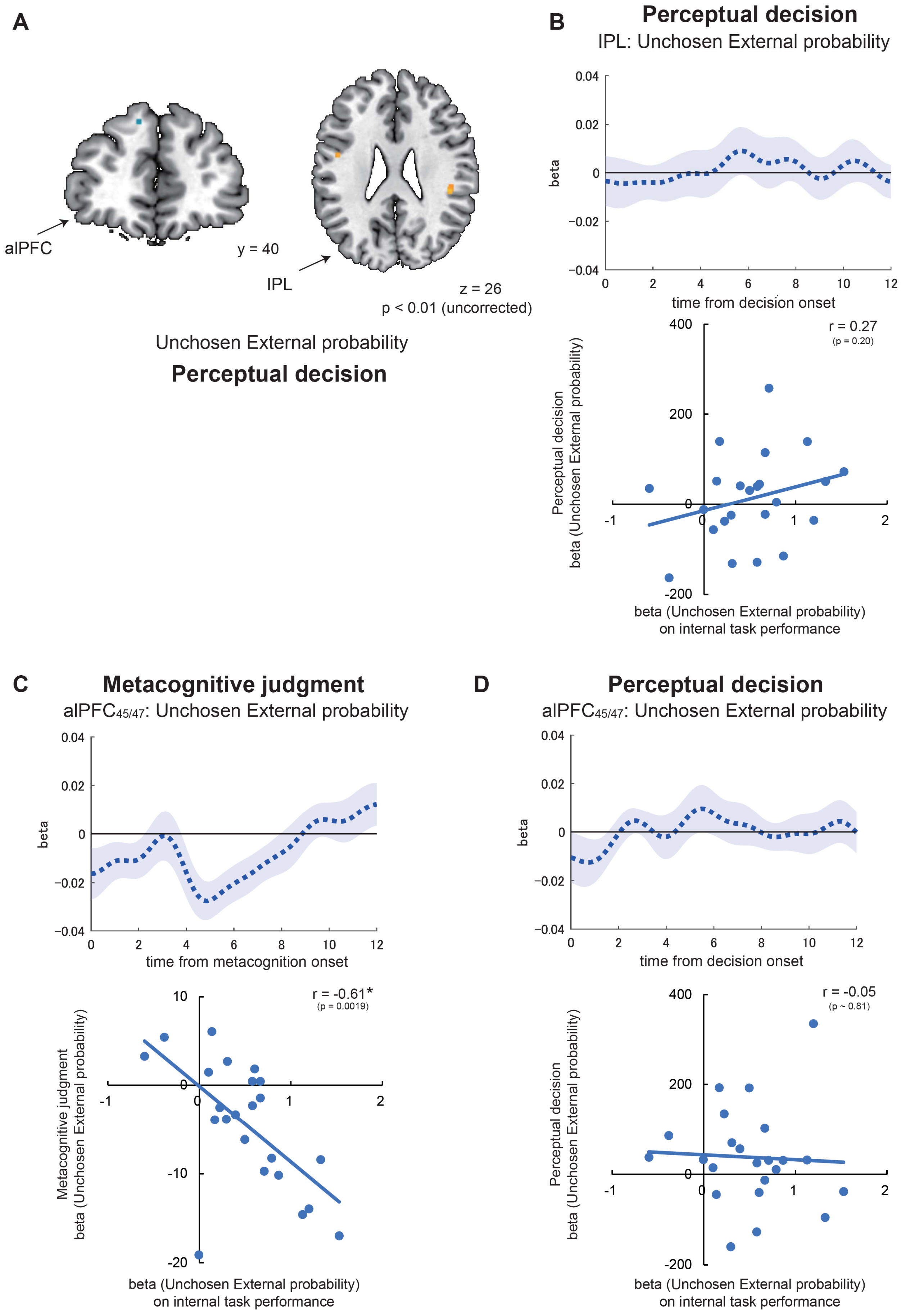

A
aIPFC
y = 40
IPL
z = 26
p < 0.01 (uncorrected)
Unchosen External probability
Perceptual decision
B
Perceptual decision
IPL: Unchosen External probability
beta
time from decision onset
Perceptual decision
beta (Unchosen External probability)
r = 0.27
(p = 0.20)
beta (Unchosen External probability)
on internal task performance
C
Metacognitive judgment
aIPFC45/47: Unchosen External probability
beta
time from metacognition onset
Metacognitive judgment
beta (Unchosen External probability)
r = -0.61*
(p = 0.0019)
beta (Unchosen External probability)
on internal task performance
D
Perceptual decision
aIPFC45/47: Unchosen External probability
beta
time from decision onset
Perceptual decision
beta (Unchosen External probability)
r = -0.05
(p ~ 0.81)
beta (Unchosen External probability)
on internal task performance

Supplemental Figure 6. Activity in alPFC during metacognitive judgment is not explained by attentional modulation as a function of the stakes of reward indicated by external probability (related to Figure 5)

(A) Activity in alPFC (Figure 5A) and IPL (attention area; Figure S3D) were not related to unchosen external probability options, which reflects the reward stakes, at the time of perceptual decision even at a very liberal statistical criterion ($p<0.01$, uncorrected). **(B)** (top) The evolution of the regression weights across time indexing the impact of unchosen external probability during perceptual decision at IPL. (bottom) The beta of IPL activity was not correlated with the influence of external probability on perceptual decision performance on the internal task (Figure 3C, left, blue) across participants ($r=0.27$, $p=0.20$). **(C)** (top) The evolution of the regression weights across time indexing the impact of unchosen external probability during metacognitive judgment at $alPFC_{47/45}$. (bottom) The beta of $alPFC_{47/45}$ activity was significantly correlated with the influence of external probability on perceptual decision performance on the internal task across participants ($r=-0.61$, $p=0.0019$). Note that the betas of BOLD and performance improvement are entirely independent. The former were measured during the metacognitive judgment stage but the latter were assessed during the perceptual decision stage. These observations suggest that alPFC also reflects external probability even if it is only in order to compare it with internal probability during prospective metacognitive judgment. **(D)** (top) The evolution of the regression weights across time indexing the impact of unchosen external probability during perceptual decision at $alPFC_{47/45}$. (bottom) The beta of $alPFC_{47/45}$ activity measured during metacognitive judgments was not significantly correlated with the influence of external probability on subsequent perceptual decisions involving the internal task across participants ($r=-0.05$, $p=0.81$).

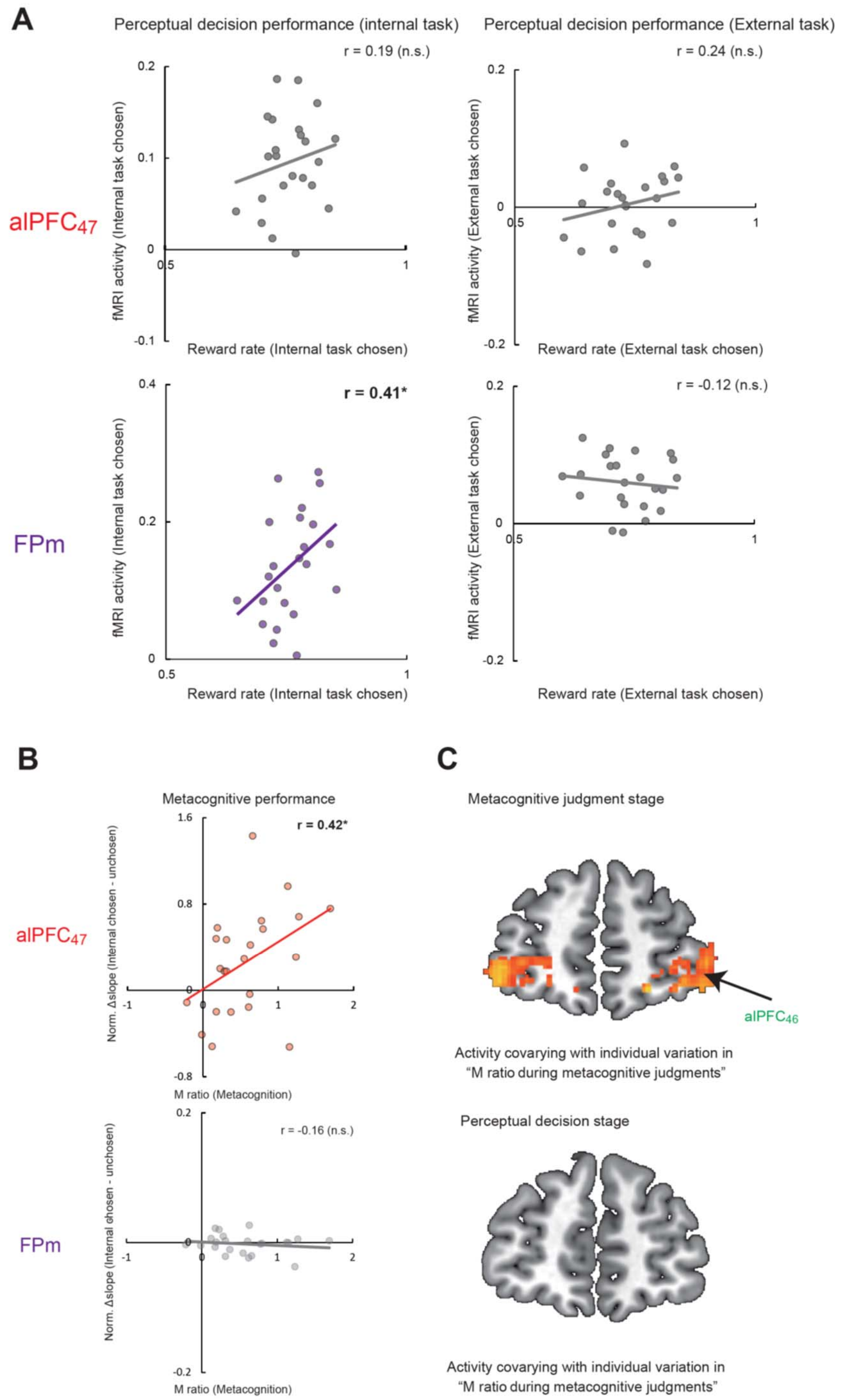

Supplemental Figure 7. Individual variation in FPm activity patterns was related to individual variation in perceptual decision making patterns in the internal probability task but not in the external probability task (related to Figure 6).

(A) FPm activity was significantly correlated with internal task performance during perceptual decision making but not with external task performance. alPFC$_{47}$ activity was correlated with neither internal nor external probability task

performance at the perceptual decision stage. **(B)** Because type-II A_{ROC} can be affected by type-I A_{ROC}, we also examined the neural correlates of metacognitive performance using the M-ratio index (meta-d' / d') (Maniscalco and Lau, 2012). M-ratio (meta-d' / d') was correlated with $alPFC_{47}$ activity ($r=0.43$, $p=0.04$) but not with FPm activity ($r=-0.17$, $p=0.43$). Once again, there was a significant difference in correlation coefficients indexing alPFC activity effects or FPm effects and individual variation in metacognitive performance (ΔFisher's $z=1.96$, $p=0.048$). **(C)** The difference in activity modulation associated with the chosen and rejected internal probability task options in $alPFC_{46}$ covaried with individual variation in metacognitive judgment accuracy as indexed by M-ratio during metacognitive judgment (top) but not during perceptual decision (bottom). (N=23; illustration shows whole-brain effects family-wise error cluster corrected with $z>2.3$ and $p<0.05$ for display purpose; $*p<0.05$).

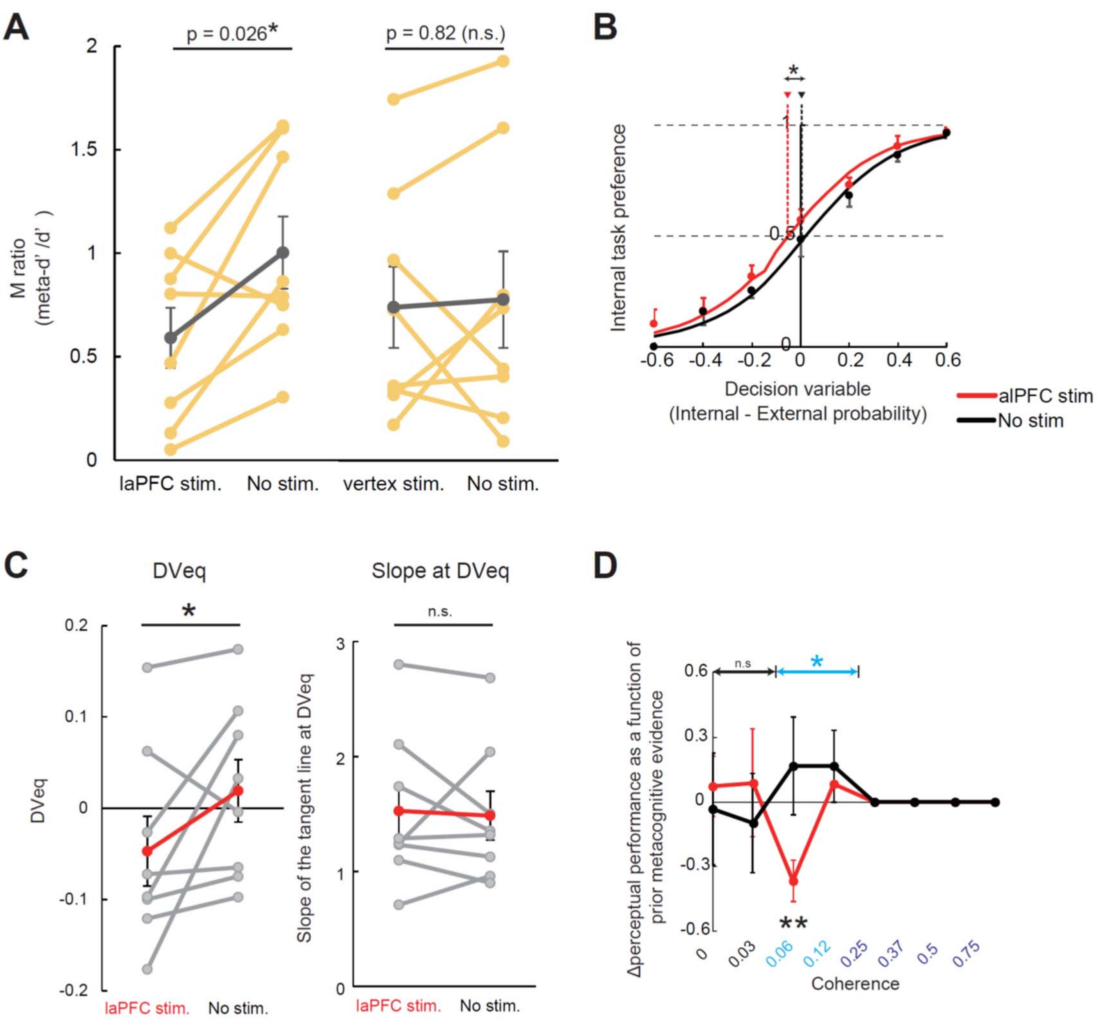

Supplemental Figure 8. Changes of preference in metacognitive choice after alPFC cTBS (related to Figure 7).

(A) Comparisons of M-ratio (meta-d'/d') between stimulation and no-stimulation for alPFC stimulation (left) and vertex stimulation (right). **(B)** Preference for choosing the internal probability task as a function of decision variable (DV). We tested whether participants' preferences for internal probability options during metacognitive judgments changed as a function of the difference between external and internal probability; this is the key decision variable (DV) that should guide metacognitive judgments (see also Figure S2B). $alPFC_{47}$ stimulation, compared to no-stimulation induced a significant bias towards choosing the internal task (change in the equilibrium point [DVeq], i.e. that is where the difference between internal and external probability is zero and the probability of choosing the internal task is 50%). **(C)** The equilibrium point (DVeq), at which the participants chose internal and external

probability options with equal frequency, significantly decreased when alPFC was stimulated; participants chose the internal option more often suggesting they overestimated their likely perceptual performance on internal options in the second perceptual decision stage (alPFC$_{47}$ stimulation, DVeq=-0.047±0.038; no stimulation, DVeq=0.019±0.034; signed-rank test between DVeq: p=0.040) (left panel). However, the sensitivity of metacognitive judgment to DV did not change (right panel; alPFC$_{47}$ stimulation, slope of the tangent line at DVeq=1.52±0.053; no stimulation, slope at DVeq=1.48±0.21; signed-rank test between DVeq: p=0.64). Red line indicates mean across participants. **(D)** Comparisons of perceptual performance improvement for Challenge trials against Inevitable trials between alPFC$_{47}$ stimulation (red) and no-stimulation (black). (N=8; error bars indicate SEM across participants; *p<0.05).
